# Supplementary material for: Human assumed central sensitisation (HACS) in patients with chronic low back pain radiating to the leg (CLaSSICO study)
Source: BMJ Open. 2022 Jan 13;12(1):e052703. doi: 10.1136/bmjopen-2021-052703 (PMC8762136; doi:10.1136/bmjopen-2021-052703)
Supplement: Supplementary data [file bmjopen-2021-052703supp002.pdf]

Appendix B: Intervention procedures

dSNRB

Diagnostic selective nerve root blocks (dSNRB) are performed in a fluoroscopy suite with monitoring equipment. At a minimum, pulse and blood pressure should be monitored every 1 to 5 minutes, depending on the procedure. Assisting personnel include nurses trained in advanced cardiac life support and a radiology technician. All procedures are performed with fluoroscopic guidance. Patient monitoring does not end after injecting medication. Post-procedure assessment is necessary because side effects or complications such as allergic reaction, vasovagal response, cardiac or respiratory depression, or high spinal block can occur. To ascertain whether the diagnostic selective nerve root block response was positive or negative, patients should perform activities or assume positions that invariably incite their pain. If a change in preprocedure and postprocedure verbal numeric rating scale rating of a minimum of 50% pain relief occurs, then the dSNRB result is considered positive.
For a lumbar dSNRB, the patient is placed in a prone position on the fluoroscopy table. The back is prepared and draped in a sterile manner. The level is located by establishing the lowest lumbar segment. The C-arm is rotated in the oblique position to allow access to the intervertebral foramen. Care must be maintained not to start too laterally to avoid renal or peritoneal puncture. A skin wheal is raised with 1% xylocaine. A 22-gauge, 6 or 10 cm with 5mm nonisolated tip needle is directed toward the foramen in the oblique position. Once the needle has penetrated the muscle adjacent to the spine, the needle then is advanced slowly in the AP plane to the 6-o'clock position of the pedicle and just inferior to the pedicle. The needle's position is in the safe triangle formed by the pedicle, the foramen's outer line, and the spinal nerve. Care is taken not to pierce the spinal nerve. Needle position is checked in the AP and lateral planes. One millilitre of non-ionic contrast agent is injected to outline the spinal nerve and dorsal root ganglion. The contrast pattern is evaluated to ensure that there is no vascular pattern, subarachnoid flow, or epidural spread to adjacent levels. One millilitre of 0.75% 0.3ml with Visipaque 320 mg l/ml 0.3ml (total 0.6ml) is infused.

Reference

Gauci, C. A. (2011). Manual of RF Techniques-a Practical Manual of Radiofrequency Procedures in Chronic Pain Management(3th Edition), CoMedical, 2011. Ridderkerk, the Netherlands.

Huston, C. W., & Slipman, C. W. (2002). Diagnostic selective nerve root blocks: indications and usefulness*. Physical medicine and rehabilitation clinics of North America*, 13(3), 545-565.

Perdok, J.M., Wolff, A.P., Stellema, R., Groen, G.J. (2017) 'Wortelblokkades'. Local protocol University Medical Center Groningen.

tSNRB

Therapeutic selective nerve root block (tSNRB) is similar to the dSNRB. The tSNRB is performed under sterile conditioned with an image intensifier. The target point was "safe triangle," i.e., above the exiting nerve root and below the corresponding pedicle. The skin is anaesthetised with 2-3 ml mepivacaine 2%. A spinal needle (22G) was inserted paramedian through the skin and muscles in a cranio-medial direction until a bony contact was encountered. This method allows advancing the needle in a safe triangle without contact with the nerve root. After verifying a correct needle positioning under biplanar image intensifier control, non-ionic contrast was injected until a radiculogram is obtained. Subsequently, 1ml 0.75% Bupivacaine and 1ml (40mg) Triamcinolon and 320mg I/ml Visipaque is injected. It should be stressed that this was a periradicular and not an intraneural injection. This technique is for the nerve roots L3-L5; for S1, a different technique is required. First, the image intensifier is positioned perpendicular to the foramen S1. A spinal needle is inserted perpendicular to the surface of the sacrum into the foramen. An image intensifier checks the correct needle positioning in two planes. After obtaining a correct periradiculargram, 1ml 0.75% Bupivacaine and 1ml (40mg) Triamcinolon and 320mg I/ml Visipaque is injected. The patients have clinical surveillance on the ward for about 30 minutes to account for unexpected side effects.

Reference

Bogduk N, Aprill C, Derby R, Selective nerve root blocks. In: Wilson DJ, ed. Interventional Radiology of the Muskuloskeletal System. London: Edward Arnold; 1995. p. 122–32.

Gauci, C. A. (2011). Manual of RF Techniques-a Practical Manual of Radiofrequency Procedures in Chronic Pain Management(3th Edition), CoMedical, 2011. Ridderkerk, the Netherlands.

Perdok, J.M., Wolff, A.P., Stellema, R., Groen, G.J. (2017) 'Wortelblokkades'. Local protocol University Medical Center Groningen.

Narozny, M., Zanetti, M., & Boos, N. (2001). Therapeutic efficacy of selective nerve root blocks in the treatment of lumbar radicular leg pain. *Swiss medical weekly*, *131*(0506).

pRF

Pulsed radiofrequency (pRF) is in a procedure similar to the tSNRB. The pRF is performed under sterile conditioned with an image intensifier. The target point was "safe triangle," i.e., above the exiting nerve root and below the corresponding pedicle. The skin is anaesthetised with 2-3 ml mepivacaine 2%. A spinal needle (22G) was inserted paramedian through the skin and muscles in a cranio-medial direction until a bony contact was encountered. This method allows advancing the needle in a safe triangle without contact with the nerve root. After verifying a correct needle positioning under biplanar image intensifier control, non-ionic contrast was injected until a radiculogram is obtained.

The tissue temperature is held at or below 42˚C on average. Radiofrequency is delivered in short, high-intensity bursts so that the radiofrequency electric field strength is increased without gross heating. The clinical objective is a neural modification by electric and thermal fields. This technique is for the nerve roots L3-L5; for S1, a different technique is required. First, the image intensifier is positioned perpendicular to the foramen S1. A spinal needle is inserted perpendicular to the surface of the sacrum into the foramen. An image intensifier checks the correct needle positioning in two planes. After obtaining a correct periradiculargram, the tissue temperature is held at or below 42˚C on average. Radiofrequency is delivered in short, high-intensity bursts so that the radiofrequency electric field strength is increased without gross heating. The patients have clinical surveillance on the ward for about 30 minutes to account for any unexpected side effects

Reference

Ewertowska, E., Mercadal, B., Muñoz, V., Ivorra, A., Trujillo, M., & Berjano, E. (2018). Effect of applied voltage, duration and repetition frequency of RF pulses for pain relief on temperature spikes and electrical field: a computer modelling study. *International Journal of Hyperthermia*, *34*(1), 112-121.

Gauci, C. A. (2011). Manual of RF Techniques-a Practical Manual of Radiofrequency Procedures in Chronic Pain Management(3th Edition), CoMedical, 2011. Ridderkerk, the Netherlands.

Perdok, J.M., Wolff, A.P., Stellema, R., Groen, G.J. (2017) ‘Wortelblokkades’. Local protocol University Medical Center Groningen.
