# Supplementary figures and images for: Human assumed central sensitisation (HACS) in patients with chronic low back pain radiating to the leg (CLaSSICO study)
Source: BMJ Open. 2022 Jan 13;12(1):e052703. doi: 10.1136/bmjopen-2021-052703 (PMC8762136; doi:10.1136/bmjopen-2021-052703)

**Appendix C:** Images drawing the most painful spot on the leg.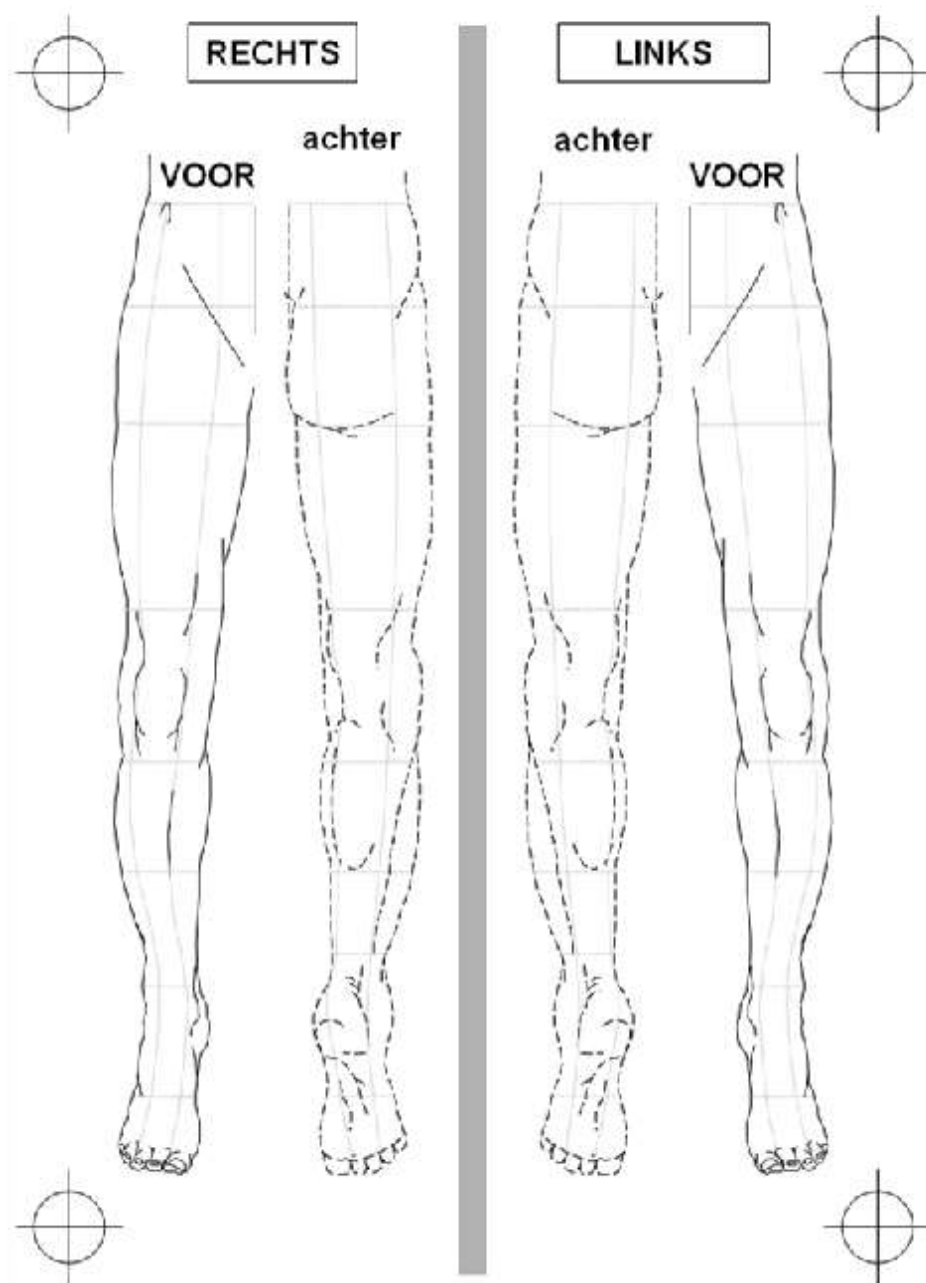

Supplement: Supplementary data [file bmjopen-2021-052703supp003.pdf]
