# Supplementary material for: Human assumed central sensitisation (HACS) in patients with chronic low back pain radiating to the leg (CLaSSICO study)
Source: BMJ Open. 2022 Jan 13;12(1):e052703. doi: 10.1136/bmjopen-2021-052703 (PMC8762136; doi:10.1136/bmjopen-2021-052703)
Supplement: Supplementary data [file bmjopen-2021-052703supp001.pdf]

APPENDICES

Appendix A: Informed consent materials:

A model consent form and other related documentation is, in Dutch, given to participants and authorised surrogates

Bijlage B:

Toestemmingsformulier proefpersoon

Onderzoek naar de gevoeligheid van het centrale zenuwstelsel bij proefpersonen met chronische lage rugpijn uitstralend naar het been.

(Centrale sensitisatie in proefpersonen met chronische lage rugpijn uitstralend naar het been)

- Ik heb de informatiebrief gelezen. Ook kon ik vragen stellen. Mijn vragen zijn voldoende beantwoord. Ik had genoeg tijd om te beslissen of ik meedoe.
- Ik weet dat meedoen vrijwillig is. Ook weet ik dat ik op ieder moment kan beslissen om toch niet mee te doen of te stoppen met het onderzoek. Daarvoor hoef ik geen reden te geven.
- Ik weet dat sommige mensen mijn persoonsgegevens en/of gecodeerde onderzoeksgegevens kunnen inzien. Die mensen staan vermeld in deze informatiebrief.
- Ik geef toestemming om mijn gegevens nog 15 jaar na dit onderzoek te bewaren.
- Ik wil meedoen aan dit onderzoek.

Naam proefpersoon:

Handtekening: Datum : __ / __ / __

Ik verklaar dat ik deze proefpersoon volledig heb geïnformeerd over het genoemde onderzoek.

Als er tijdens het onderzoek informatie bekend wordt die de toestemming van de proefpersoon zou kunnen beïnvloeden, dan breng ik hem/haar daarvan tijdig op de hoogte.

Naam onderzoeker (of diens vertegenwoordiger):

Handtekening: Datum: __ / __ / __

<indien van toepassing>

Aanvullende informatie is gegeven door:

Naam:

Functie:

Handtekening: Datum: __ / __ / __

----

* Doorhalen wat niet van toepassing is.

De proefpersoon krijgt een volledige informatiebrief mee, samen met een kopie van het getekende toestemmingsformulier.

Schuttert I, *et al. BMJ Open* 2022; 12:e052703. doi: 10.1136/bmjopen-2021-052703
