# Supplementary material for: Human assumed central sensitisation (HACS) in patients with chronic low back pain radiating to the leg (CLaSSICO study)
Source: BMJ Open. 2022 Jan 13;12(1):e052703. doi: 10.1136/bmjopen-2021-052703 (PMC8762136; doi:10.1136/bmjopen-2021-052703)
Supplement: Author's manuscript [file bmjopen-2021-052703.draft_revisions.pdf]

BMJ Open is committed to open peer review. As part of this commitment we make the peer review history of every article we publish publicly available.

When an article is published we post the peer reviewers' comments and the authors' responses online. We also post the versions of the paper that were used during peer review. These are the versions that the peer review comments apply to.

The versions of the paper that follow are the versions that were submitted during the peer review process. They are not the versions of record or the final published versions. They should not be cited or distributed as the published version of this manuscript.

BMJ Open is an open access journal and the full, final, typeset and author-corrected version of record of the manuscript is available on our site with no access controls, subscription charges or pay-per-view fees (http://bmjopen.bmj.com).

If you have any questions on BMJ Open's open peer review process please email info.bmjopen@bmj.com

BMJ Open

Human Assumed Central Sensitisation (HACS) in Patients with Chronic Low Back Pain Radiating to the Leg (CLaSSICO study):Protocol for a Prospective Observational Study.

Journal:	*BMJ Open*
Manuscript ID	bmjopen-2021-052703
Article Type:	Protocol
Date Submitted by the Author:	26-Apr-2021
Complete List of Authors:	Schuttert, Ingrid; UMCG, Department of Anesthesiology, Pain Center<br>Timmerman, Hans; UMCG, Department of Anesthesiology, Pain Center<br>Groen, Gerbrand; UMCG, Department of Anesthesiology, Pain Center<br>Petersen, Kristian; Aalborg Universitet, Department of Health Science and Technology<br>Arendt-Nielsen, Lars; Aalborg Universitet, Department of Health Science and Technology<br>Wolff, Andre; Universitair Medisch Centrum Groningen, Department of Anesthesiology, Pain Center
Keywords:	Pain management < ANAESTHETICS, Back pain < ORTHOPAEDIC & TRAUMA SURGERY, Neurological pain < NEUROLOGY, PAIN MANAGEMENT

SCHOLARONE™
Manuscripts

Human Assumed Central Sensitisation (HACS) in Patients with Chronic Low Back Pain Radiating to the Leg (CLaSSICO study):

Protocol for a Prospective Observational Study.

AUTHORS

Ingrid Schuttert*, MSc, Department of Anesthesiology, Pain Center, University of Groningen, University Medical Center Groningen (UMCG), Groningen, the Netherlands. OrcID: 0000-0003-2164-4721

Hans Timmerman, PhD, Department of Anesthesiology, Pain Center, University of Groningen, University Medical Center Groningen (UMCG), Groningen, the Netherlands. OrcID: 0000-0002-6082-9043

Gerbrand J. Groen, MD, PhD, Department of Anesthesiology, Pain Center, University of Groningen, University Medical Center Groningen (UMCG), Groningen, the Netherlands. OrcID: 0000-0002-7199-0107

Kristian K. Petersen, PhD, SMI, Center for Neuroplasticity and Pain, Department of Health Science and Technology, Faculty of Medicine, Aalborg University, Aalborg, Denmark. ORCID: 0000-0003-4506-000X

Lars Arendt-Nielsen, PhD, SMI, Center for Neuroplasticity and Pain, Department of Health Science and Technology, Faculty of Medicine, Aalborg University, Aalborg, Denmark. ORCID: https://orcid.org/0000-0003-0892-1579

André P. Wolff, MD, PhD, Department of Anesthesiology, Pain Center, University of Groningen, University Medical Center Groningen (UMCG), Groningen, the Netherlands. OrcID: 0000-0001-6240-3903

* Corresponding author for public and scientific queries:

Ingrid Schuttert
Department of Anesthesiology, UMCG Pain Center, University Medical Center Groningen (UMCG), PO Box 30001, 9751 ND Haren, the Netherlands.
Email: i.schuttert@umcg.nl
Tel: +31503612140

Word count: 4147(Word count, excluding title page, abstract, references, figures and tables)

For peer review only

DECLARATIONS

Ethics approval and consent to participate

The medical and ethical review board Committee on Research Involving Human Subjects of the University Medical Center Groningen, Groningen, the Netherlands, has approved the study "Central sensitisation in patients with chronic low back pain radiating to the leg", dossier number NL60439.042.17 on November 1, 2017. Due to COVID-19, an amendment was submitted and approved on August 11, 2020, to prolong the participants' inclusion date due to March 1, 2022. The participants will receive written and oral information about the study and all assessments. The participants will receive written and oral information about the study and assessments. Before the start of the study, written informed consent will be provided by all participants (see appendix A).

Consent for publication

Not applicable.

Availability of data and materials

Due to the data's sensitivity and the restrictions from informed consent, the data will not be stored in a public repository. The data and meta-data will be stored at a repository at the UMCG, ensuring the data's security and backup. UMCG pursues a FAIR data policy for research conducted in the UMCG. To make the data findable and accessible for others, we will include a description of the UMCG data catalogue data: https://www.groningendatacatalogus.nl/. The data's metadata will be made available for researchers inside and outside the institute via the catalogue. This catalogue is in sync with relevant (inter)national catalogues, such as BBMRI and NARCIS. A data access committee has been put in place to review requests and assure the accessibility of the data. This access committee can be reached via the corresponding author (Ingrid Schuttert, e-mail: i.schuttert@umcg.nl or André Wolff, e-mail: a.p.wolff@umcg.nl). The results will be published in peer-reviewed scientific journals and submitted for presentation at relevant national and international scientific meetings/conferences.

Acknowledgements

Not applicable.

Patient consent for publication

Not required.

For peer review only - http://bmjopen.bmj.com/site/about/guidelines.xhtml

Keywords

Chronic Low Back Pain; Sensitisation; Study protocol; Quantitative sensory testing (QST); Selective nerve root block (SNRB); Pulsed radiofrequency (PRF), Human Assumed Central Sensitisation (HACS).

For peer review only

For peer review only - http://bmjopen.bmj.com/site/about/guidelines.xhtml

ABBREVIATIONS

ANOVA:	ANalysis Of VAriance
BBMRI:	Biobanking and Biomolecular Resources Research Infrastructure
CaU:	Care as Usual
ClaSSICO:	Central sensitisation in patients with chronic low back pain radiating to the leg
CLBPr:	Chronic Low Back Pain radiating to the leg;
COVID-19:	coronavirus disease 2019
CPM:	Conditioned Pain Modulation;
CRF:	Case report file
CS:	Central Sensitisation
CSI:	Central Sensitisation Inventory;
Cuff PPT:	Pressure Pain Threshold by cuff algometer;
Cuff TS:	Temporal Summation by cuff algometer;
DFNS:	Deutscher Forschungsverbund Neuropathischer Schmerz (German Research Network on Neuropathic Pain)
DMA:	Dynamic Mechanical Allodynia
dSNRB:	Diagnostic Selective Nerve Root Block
EEG:	Electroencephalogram
fMRI:	Functional magnetic resonance imaging
HACS	Human Assumed Central Sensitisation
IQR:	Interquartile range
MDT:	Mechanical Detection Threshold
METc:	Medisch ethische toetsingscommissie (Medical ethical review board)
MPT:	Mechanical Pain Threshold

For peer review only - http://bmjopen.bmj.com/site/about/guidelines.xhtml

NARCIS:	National Academic Research and Collaborations Information System
NRS:	Numerical Rating Scale
Pain Drawing:	Drawing of the pain in standard leg images.
PCS:	Pain Catastrophising Scale
PDI:	Pain Disability Inde
PPT:	Pressure Pain Threshold
pRF:	Pulsed Radiofrequency;
PVAQ:	Pain Vigilance and Awareness Questionnaire
QST:	Quantitative Sensory Testing;
RAND-36:	Rand 36-Item Health Survey
SBST:	STarT Back Screening Tool
SMAPC:	Specialised multidisciplinary academic pain center
SNRB:	Selective Nerve Root Block
SPSS:	Statistical Package for Social Sciences
tSNRB:	Therapeutic Selective Nerve Root Block
UMCG:	University Medical Center Groningen
VAS:	Visual Analogue Scale
WAI:	Work Ability Index
WUR:	Wind-up Response

For peer review only

For peer review only - http://bmjopen.bmj.com/site/about/guidelines.xhtml

ABSTRACT

Introduction: Patients with chronic low back pain radiating to the leg (CLBPr) are sometimes referred to a specialised pain clinic for a precise diagnosis based, for example, on a diagnostic selective nerve root block (dSNRB). Possible interventions are therapeutic selective nerve root block (tSNRB) or pulsed radiofrequency (pRF). Central pain sensitisation is not directly assessable in humans and therefore the term 'human assumed central sensitisation' (HACS) is proposed. The possible existence and degree of sensitisation associated with pain mechanisms assumed present in the human central nervous system, its role in the chronification of pain and its interaction with diagnostic and therapeutic interventions are largely unknown in patients with CLBPr. The aim of quantitative sensory testing (QST) is to estimate quantitatively the presence of HACS and accumulating evidence suggest that a subset of patients with CLBP have facilitated responses to a range of QST tests. The aims of this study are to assess HACS in patients with CLBPr, to determine associations with the effect of SNRBs and compare outcomes of HACS in patients to healthy volunteers.

Methods and analysis: A prospective observational study including fifty patients with CLBPr. Measurements are performed before diagnostic and therapeutic nerve root block interventions and at four weeks follow-up. Data from patients will be compared with those of fifty sex- and age-matched healthy volunteers. The primary study parameters are the outcomes of QST and the central sensitisation inventory. Statistical analyses to be performed will be (repeated measures) analysis of variance and correlation assessments.

Ethics and dissemination: The Medical Research Ethics Committee of the University Medical Center Groningen, Groningen, the Netherlands, approved this study (dossier NL60439.042.17). The results will be disseminated via publications in peer-reviewed journals and at conferences.

Trial registration number: NTR NL6765

For peer review only - http://bmjopen.bmj.com/site/about/guidelines.xhtml

Article Summary

STRENGTHS AND LIMITATIONS OF THIS STUDY

- Strength: A longitudinal study to follow patients during diagnostic and therapeutic nerve root blocks and four weeks after the intervention to increase insights in the intervention's effect.
- Strength: comparison with sex- and age-matched healthy controls.
- Strength: Multimodal assessment of peripheral and HACS via objective and subjective measures.
- Limitation: The order of the QST measures is not randomised for each patient and each visit and is only randomised per visit.

For peer review only

For peer review only - http://bmjopen.bmj.com/site/about/guidelines.xhtml

INTRODUCTION

Background:

The mean incidence and prevalence of chronic low back pain radiating to the leg (CLBPr) are 9.4 and 17.2 per 1000 person-years respectively.[1] If the diagnosis of CLBPr is unclear despite extensive neurological, orthopaedic and radiological examination, patients are sometimes referred to a specialised multidisciplinary pain clinic, where a diagnostic selective nerve root block (dSNRB) might provide a more precise diagnosis.[2 3] This block, achieved by injection of a local anaesthetic, can help determine the predominant segmental level of the pain.[4-9] A positive dSNRB is generally followed by an injection of local anaesthetics and corticosteroids, i.e. a therapeutic SNRB (tSNRB) or a pulsed radiofrequency treatment (pRF) of the spinal nerve root, its dorsal ganglion or a combination thereof.

Growing evidence suggests that central pain mechanisms are facilitated in a subset of patients with CLBPr.[10 11] Facilitation of peripheral and central pain mechanisms have been suggested to be associated with pain progression, poor recovery, poor response to pharmacological interventions and inadequate response to low back surgery, indicating the clinical need of these assessments.[12-14] Preclinical data suggests that nerve fibres can malfunction and respond as nociceptors, resulting in pain from light stimuli, possibly caused by peripheral and central sensitisation (CS).[15] Dysfunction of the somatosensory system may clinically lead to positive sensory symptoms (e.g. allodynia, aftersensations, enhanced temporal summation) and negative sensory symptoms (e.g. hypoesthesia).[11 16-19] Because mechanisms related to CS can currently only be assessed in animals,[11] proxies are used in humans to assess clinical signs that may be associated with the presence of central pain mechanisms. We use the term 'human assumed central sensitisation' (HACS) because there is no gold standard to estimate the presence of CS in humans at present. Moreover, there is no clear definition, method or (clinical) guideline applicable to diagnose HACS.

The role of HACS in the development of CLBPr and in the chronification of pain and the interaction with diagnostic and therapeutical interventions is not clear.[11] Several methods are thought to assist in the clinical diagnosis of HACS.[11] With Quantitative Sensory Testing (QST) we measure the responses to calibrated graded innocuous or noxious stimuli [20] and evaluate the presence of HACS in patients with CLBPr.[21] Mehta et al. showed that following pRF treatment patients with CLBPr have increased pressure pain thresholds (PPT) and increased conditioned pain modulation (CPM), suggesting a normalization of sensory pain profile.[21] The presence of HACS might also be evaluated with the Central Sensitisation

For peer review only - http://bmjopen.bmj.com/site/about/guidelines.xhtml

Inventory (CSI),[22] but there are no clinical associations established between the CSI and experimental measures.[11]

Objectives:

The primary objective of this study is to determine HACS changes caused by therapeutic selective nerve root block or pulsed radiofrequency in patients with chronic low back pain radiating to one leg. Secondary objectives are (1) to assess HACS in patients with chronic low back pain radiating to one leg, (2) to determine the change in HACS caused by diagnostic selective nerve root block in patients with chronic low back pain radiating to one leg, and (3) to compare the differences in HACS assessment results between patients (before and after selective nerve root blocks) and healthy volunteers.

Trial design:

A prospective longitudinal observational study about HACS in patients with CLBPr who undergo SNRBs compared to sex- and age-matched healthy volunteers.

For peer review only

METHODS

This study was registered prospectively in the Netherlands Trial Register: Trial NL6765 (first posted January 10, 2018). The trial information in this protocol is in line with the WHO Trial Registration Data Set [23] and written following the SPIRIT 2013 Guideline.[24]

Participants, Interventions and outcomes

Study setting:

Patient recruitment, treatment, and measurements take place in the specialised multidisciplinary academic pain center (SMAPC), University Medical Center Groningen (UMCG), Groningen, the Netherlands.

Patient and public involvement:

Patients were not involved in the development of the research question and the determination of the outcome measures. During recruitment, patients are asked to suggest healthy volunteers whose age and gender match their own to participate in the study.

Eligibility criteria:

Fifty consecutive adult patients of both sexes with chronic low back pain radiating in the dermatomes L3 to S2 and fifty sex- and age-matched healthy volunteers will be included. The inclusion and exclusion criteria are summarised in table 1 (Table 1). The inclusion criteria for the healthy volunteers are shown in table 2 (Table 2).

Patients can retain their ongoing pain medication or discontinue it if they stop feeling pain during the study. Other changes in medication use are not allowed during participation. Moreover, all other pain interventions (e.g. physical therapy, transcutaneous electrical nerve stimulation) during their participation in this study are prohibited. Healthy volunteers are matched with the patients on sex and age (plus or minus three years of age). As mentioned, patients are asked to suggest suitable healthy volunteers. If necessary healthy volunteers are also recruited via flyers placed near to the UMCG facilities. Healthy volunteers do not undergo SNRBs. Patients and healthy volunteers may withdraw from the study at any time for any reason if they wish to do so and without any consequences. The investigator can decide to withdraw a participant from the study for urgent medical reasons.

For peer review only - http://bmjopen.bmj.com/site/about/guidelines.xhtml

Interventions:

The intervention provided in this study is performed in the same way as the standard intervention provided in the SMAPC and follows the guideline for SNRB (see appendix B for the detailed procedure). Table 3 shows which intervention is used in each visit (Table 3).

Participant timeline:

Patients and healthy volunteers receive a verbal and written explanation of the study procedures and are given sufficient time to consider their participation. After written informed consent is provided, a meeting is scheduled. The flowchart of the study is shown in figure 1 (Figure 1). The schedule for the patient and the healthy volunteer assessments are shown in table 3 (Table 3). Visit 1b, and 1c are only performed for the patients when more than one dSNRB is necessary. After the dSNRB, both numerical rating scale (NRS) for both pains in the back and the leg are assessed. In case of a pain reduction of ≥ 50%, the patient is considered to have a "positive result", and the therapeutic intervention is planned. The responsible physician will decide which intervention (tSNRB or pRF treatment) each patient will receive (which is part of care as usual (CaU)). If a pain reduction is < 50%, the patient is considered to have a "negative result". With a negative result, the patient will undergo another dSNRB at a different spinal level (3 dSNRBs are the maximum within this study). Diagnostic and therapeutic SNRBs and pRF will be performed according to the standard procedures of the SMAPC. Healthy volunteers are not asked to draw their pain area and do not receive any diagnostic or therapeutic intervention. For healthy volunteers, only baseline assessment is performed.

Recruitment:

At the SMAPC, on average, two new patients with CLBPr are seen per week. If two patients per month agree to join the study, the sample will be completed within four years. Because patients must visit the SMAPC once more than during usual CaU care (visit 3), they will be compensated for this visit when it is impossible to schedule it with another regular hospital visit. Patients will receive compensation for extra travel costs (€0.19/km). Healthy volunteers will receive 10 euros for their visit plus compensation for travel costs (€0.19/km).

Duration

Each visit will last no longer than two and a half hours, consisting of 1 hour measurements (patients and healthy volunteers), 0.5 hour for the treatment and 0.5-1 hour for the assessment of the effect of the treatment (patients). Interventions are performed one week apart, and visit 3 will be four weeks after the therapeutic intervention. Depending on the

For peer review only - http://bmjopen.bmj.com/site/about/guidelines.xhtml

number of dSNRBs performed, the total duration of this study for the patient will take between five to seven weeks (3-5 visits). Healthy volunteers will undergo only one visit with an assessment of 1 hour.

The first patient was included on June 5, 2018. the planned end date for the inclusion of participants is March 1, 2022.

Outcomes:

All measurements are performed for study-specific reasons. The primary outcomes are QST measures and the CSI. Secondary outcomes are Pain Catastrophising Scale (PCS), Pain Vigilance and Awareness Questionnaire (PVAQ), 36-Item Short-Form Health Survey (SF-36), Pain Disability Index (PDI), Work Ability Index (WAI), STarT Back Screening Tool (SBST), Numeric Rating Scale (NRS) and drawing in standard leg images (pain drawing) (Table 3).

Descriptive data:

Descriptive items include: age (years), sex (male /female), weight (kg), height (cm), education, comorbidities, medication and ethnic background.

Questionnaires:

One or two days before each visit, the patient receives an e-mail with a link and is asked to fill out the questionnaires (Table 3). The questionnaires (provided in Dutch) are sent through RoQua (UMCG, Groningen, The Netherlands), a questionnaire program built into the electronic patient's file. The whole set of questionnaires is sent for visit 1a and 3, and it takes about 20 minutes to complete. For visit 1b-1c and 2, only the CSI is sent, and take about 5 minutes to complete. Patients who cannot fill out the questionnaires online will receive them on paper at the beginning of the visit.

Central Sensitisation Inventory (CSI)[22 25]*:* The CSI contains 25 statements related to current health symptoms. Each item is measured on a 5-point Likert scale from 0 (never) to 4 (always). A cut-off score of 40 is being used[22 25]. The original CSI (Cronbach's alpha = 0.879, test-retest reliability = 0.817)[25] as well as the CSI-Dutch language version (Dlv) (Cronbach's alpha = 0.91, test-retest reliability = 0.88 and 0.91)[22] shows a good internal consistency.

Pain Catastrophizing Scale (PCS)[26]*:* The PCS assesses thoughts and feelings about pain. For each of 13 statements the participant is asked to answer on a scale from 0 (totally not) to 4 (always). The original PCS (Cronbach's alpha = 0.93[27], test-retest reliability = 0.75[26]) as

For peer review only - http://bmjopen.bmj.com/site/about/guidelines.xhtml

well as the PCS-Dlv (Cronbach's alpha between 0.85 and 0.91)[28-30] show good internal consistency.

Pain Vigilance and Awareness Questionnaire (PVAQ)[31 32]: PVAQ measures awareness of pain. The participant can assign a number from 0 (never) to 5 (continuously) for 16 statements. The original PVAQ shows good internal consistency (Cronbach's alpha=0.86[31]) and adequate test-retest reliability (r=0.80[31]). The PVAQ-Dlv also shows good internal consistency (Cronbach's alpha = 0.87[32]).

RAND 36-item Health Survey (RAND-36):[33 34] The RAND-36 is a participant-reported survey of health-related quality of life. It consists of 8 sections: vitality, physical functioning, bodily pain, general health perceptions, physical role functioning, emotional role functioning, social role functioning and mental health. The RAND-36-Dlv has shown to be valid and reliable.[33]

Pain Disability Index (PDI):[35 36] The PDI is used to measure the degree to which aspects of the patient's life is disrupted by chronic pain. It lists seven life activity categories (family/home responsibilities, recreation, social activity, occupation, sexual behaviour, self-care and life-support activities). The original PDI shows good internal consistency (Cronbach's alpha=0.871[37]), and the test-retest reliability is good (ICC=0.91[38]). The PDI-Dlv showed good internal consistency with patients with chronic low back pain (Cronbach's alpha= 0.85[36]) and sufficient test-retest reliability (ICC=0.78[36])

Work Ability Index (WAI):[39] The WAI is used to assess workability during health examinations. The index is determined based on the answers to a series of questions that consider the demands of work, the worker's health status and resources. The original WAI has been validated (Cronbach's alpha = 0.72).[40 41] The WAI-Dlv showed sufficient internal consistency (Cronbach's alpha = 0.72[41]) and an acceptable test-retest reliability.[42]

STarT Back Screening Tool (SBST):[43 44] The SBST is a 9-item patient self-reporting questionnaire validated for triage of patients with non-specific low back pain in primary care. It has shown to be helpful in tertiary care.[45] The SBST identifies modifiable prognostic factors from the health domains of pain, activity limitation and psychosocial factors, which are risk factors for persistent non-specific low back pain. The SBST classifies patients into three groups: low, medium or high risk of poor prognosis based on the symptom complexity. When assessed by general practitioners, this classification can assist in making decisions towards appropriate evidence-based treatment pathways. The SBST-Dlv showed good validity and an excellent to fair reproducibility.[44]

Numeric Rating Scale (NRS) for Pain: A pain rating scale where patients are asked to rate their pain at two locations (leg and back), giving a number between 0 and 10 (0 = no pain/10

For peer review only - http://bmjopen.bmj.com/site/about/guidelines.xhtml

= maximum pain imaginable). In the QST measurements, an NRS is also used with a scale from 0 to 100 10 (0 = no pain/100 = maximum pain imaginable).

Drawing in standard leg images (Pain Drawing): The patient is asked to draw, on an image with standard legs (see appendix C), the area where they experience the most pain. When more locations are drawn, an arrow will indicate the most painful spot.

Quantitative Sensory Testing (QST):

QST is a psychophysical method that measures responses to calibrated graded innocuous or noxious stimuli in addition to bedside clinical examination of the somatosensory system.[19 46] The measurements are based on the QST battery developed by the German Research Network on Neuropathic Pain (DFNS).[10 15] This protocol is extended with cuff algometry (NociTech, Aalborg, Denmark). QST is used for the analyses of HACS. The participants will be comfortable sitting and lying on a bed during all assessments when evaluating the stimuli. The sequences' order is counterbalanced and documented in the case report form (Table 4). The measurements take about 45-60 minutes.

Mechanical Detection Threshold (MDT):

MDT is assessed via von Frey-filaments (OptiHair2, MRC Systems GmbH, Heidelberg, Germany). When the 16mN von Frey filament's touch is felt, the von Frey filament's intensity is reduced until no touch is felt. The intensity then will be increased until the touch is felt again. This test will be repeated at least three times to detect the MDT. The first von Frey filament that cannot be felt is documented.[47-49]

Dynamic mechanical Allodynia (DMA):

DMA is tested using dynamic innocuous stimulus (soft brush; Sense Lab Brush no. 5, Somedic, Sösdala, Sweden). The innocuous stimulus is administered once and after that three times in a 1-2 cm wiping motion on the skin. The participant is asked to say if something was felt and to rate the NRS-pain from 0 to 100.[50-52]

Mechanical Pain Threshold (MPT):

This test is performed with weighted pinprick stimuli (MRC Systems GmbH, Heidelberg, Germany) using the method of limits. Five threshold determinations are made, each with a series of ascending and descending stimulus intensities. The first pinprick that is considered painful is documented.[51 53-55]

Wind-up Ratio (WUR):

For peer review only - http://bmjopen.bmj.com/site/about/guidelines.xhtml

In this test, the NRS-pain score (0-100) experienced for a single pinprick stimuli (256N) is compared to an applied series of repetitive (ten times with intervals of 1 second) pinprick stimuli of the same intensity (256N). The wind-up ratio is calculated by dividing the pain intensity rating for the series of stimuli by the pain intensity rating for the single stimulus.

Pressure Pain Threshold (PPT):

Using a handheld pressure algometer (Wagner FDX 10, Greenwich, USA), the threshold for pressure-induced pain is assessed by slowly increasing stimulus intensities (5 N/s). The threshold is determined when the pressure becomes painful/more than just the feeling of pressure when the participants say 'now'. The NRS-pain (0-100) and the applied force (N) at that moment are documented.

Cuff algometry (Cuff)

During cuff algometry, the participants will be instructed to continuously evaluate the pain using an electronic visual analogue scale (0 = no pain, 10 = worst imaginable pain).

Cuff pain tolerance threshold:

The cuff pressure will be increased by one kPa/s on the dominant leg. The participant will be instructed to rate the pain intensity continuously on the electronic VAS until the tolerance level is reached, and the participant pushes the stop button. When the participant pushes this button, the pressure is released immediately. The pain tolerance threshold (PTT) is defined as when the participant presses the stop button. A similar assessment will be performed on the non-dominant leg after the cuff temporal summation.

Cuff temporal summation:

A total of 10 repeated mechanical cuff pressure stimuli at the intensity of PTT will be delivered at 0.5 Hz (1-second stimulus duration and 1-second interval between stimuli) to the lower leg. A constant pressure of 1 kPa will be applied between the individual pressure stimuli sets to avoid movement of the cuff. The participants will continuously rate the pain intensity on the electronic VAS during the 10 repeated stimuli.

Conditioned Pain Modulation (CPM):

PPT will be repeated on the deltoid muscle and the rectus femoris, both on the painful dermatome's contralateral side. The participant will be asked to put the dominant hand up to the wrist with spread fingers in a bucket filled with ice and cold water (conditioning stimulus). The amount of time (at a maximum of three minutes for safety reasons) that it takes until the

For peer review only - http://bmjopen.bmj.com/site/about/guidelines.xhtml

For peer review only

participant removes the immersed hand out of the bucket will be noted. Immediately after, PPT in the deltoid muscle and the rectus femoris both on the contralateral side of the painful dermatome will be repeated.[17 18 20]

Measurement locations:

Measures will be taken in six different points in total (Table 5). The QST measures will be performed on locations A-D. The CPM will be assessed at location D and E. The cuff measures will be performed at location F.

Sample size:

The total sample size is 100 participants (50 patients and 50 healthy volunteers for comparison), which is consistent with the sample sizes of several studies that have used QST. For example, Graven-Nielsen et al.[56] applied a QST battery in 48 patients and 21 pain-free healthy controls to determine the importance of ongoing tissue pathology in maintaining enhanced central pain processing.[56] Starkweather et al.[57] included 44 patients and 20 healthy controls and used QST to assess HACS in patients with low back pain.[57] With a power of 80% and a type I error of 5%, the number of participants was calculated (G*power for Windows, Version 3.1.9.7, Heinrich Heine Universität, Dusseldorf, Germany) to be 50 per group, resulting in a medium effect size of 0.57. This medium effect size is hypothesised because multiple QST measurements are tested with independent variables such as different interventions and demographic variables.

Data collection, management, and analysis

Data collection and management:

The primary data (raw) will be collected on paper and entered into an electronic data capture: OpenClinica (OpenClinica, LLC, Waltham, USA, version 3.14). The questionnaires will be assessed via RoQua (RoQua, UMCG, Groningen, The Netherlands) as implemented in the electronic patient files (EPIC, Epic Systems Corporation, Verona, USA, version August 2020). For the healthy volunteers' dummy numbers (for the hospital information system) will be created to enable them to access and fill in the questionnaires. The data in Open Clinica and RoQua will be combined into SPSS (version 27, IBM, Armonk, USA) database.

The raw data on paper, in OpenClinica and the data of RoQua, will be anonymised and coded. The data will be stored at the UMCG research drive.

Data will be handled confidentially. A participant identification code list will be used to link the data to the participant. The principal investigator will safeguard the key to the code.

For peer review only - http://bmjopen.bmj.com/site/about/guidelines.xhtml

Data will be accessible for the team of investigators, the Medical Ethical Review Board and the health care Inspection. Data will be saved for 15 years. Data will be used for publication, but no participant will be traceable. The handling of personal data will comply with the Dutch Personal Data Protection Act.

Statistical methods:

Data records from the different tests and questionnaires will be collected and merged into one database. SPSS software version 27.0 or higher (IBM, Armonk, USA) will be used to perform the analyses. Descriptive statistics data will be presented as means ± standard deviation (SD) when normally distributed and as median and interquartile range (IQR) (25-75) when not normally distributed. For the primary objective, an ANOVA will be performed (1. patients + tSNRB, 2. patients + pRF, 3. healthy volunteers), side (left/right), and site (4 sites tested). For secondary objective 1, Pearson's correlation analysis will be performed between the different HACS indicators. Repeated-measures Analysis of variance (ANOVA) will be used to compare the values after each intervention (dSNRB, tSNRB, pRF) with the baseline assessment. For secondary objective 2, an ANOVA will be performed (1. patients + tSNRB, 2. patients + pRF, 3. healthy volunteers), side (left/right), and site (4 sites tested). For secondary objective 3, the difference between patients (baseline vs follow-up) and healthy volunteers (only baseline) is calculated for the questionnaires and QST measurements. The calculated differences will be assessed using an unpaired sample t-test.

Monitoring

Data monitoring:

The monitoring will be carried out by research support staff based on GCP monitoring principles. The departmental research office will monitor the trial regularly. This typically includes the following checks:

1) The data collected are consistent with adherence to the trial protocol; 2) Case report files (CRFs) are completed by authorised persons; 3) No key data are missing; 4) The data appears to be valid (i.e. range and outlier checks); 5) Review of recruitment rates, withdrawals and losses to follow-up.

Harms:

Experienced specialised pain staff members perform all interventions in the care as usual setting. Study-related additional harms may consist of QST-related pain during and a short time after the measurements. No other harm is expected.

For peer review only - http://bmjopen.bmj.com/site/about/guidelines.xhtml

Protocol amendments:

The following amendments were made before the inclusion of the fifth patient. Regarding the Inclusion criteria, the age limit of 65 was excluded. The visual analogue scale (VAS) was changed to NRS because NRS is used in CaU. Before the first healthy volunteer's inclusion, the number of visits was reduced from three to one. The latest protocol, version 5, is from 06-07-2020. Due to the COVID-19 pandemic, the study was put on hold, and an amendment (version 5) was submitted and approved on August 11, 2020, to prolong the initial inclusion date for the participants to March 1, 2022.

Declaration of interest:

Not applicable

IPD sharing statement

The de-identified individual clinical trial participant-level data (IPD) will be shared. All individual participant data that underlie the results reported in this article will be shared after de-identification (text, tables, figures, and appendices). The study protocol will also be available. The data will be available beginning nine months following article publication and for a maximum period of 15 years. To make the data findable and accessible for others, we will include a description of the UMCG data catalogue data: https://www.groningendatacatalogus.nl/. Researchers who provide a methodologically sound proposal can access the data with a signed data access agreement.

For peer review only

For peer review only - http://bmjopen.bmj.com/site/about/guidelines.xhtml

DISCUSSION

In this study, the effects of SNRBs on signs associated with the assessment of HACS are evaluated in patients with CLBPr by the use of questionnaires and QST compared to sex- and age-matched healthy controls. This longitudinal study follows the CaU situation in the diagnostic phase with dSNRB's until four weeks after the therapeutic intervention (tSNRB or pRF). Measurement is performed before each intervention. The follow-up of 4 weeks is based on the optimum time at which the therapeutic effect is thought to reach its maximum.[58] This study will give an insight into the relationship between diagnostic and therapeutic SNRBs and pRF effects on HACS.

This study primarily focuses on signs that may be related to HACS because they are expected to be the underlying factors in the chronification of low back pain. A recent study found changes in PPT and CPM[21], which suggests that these factors are modifiable. In this latter study, less extensive pain measurements were used, and no sex- and age-matched healthy volunteers were included compared to our study protocol.

The QST measures in this study are based on the DFNS battery[59] and have to be performed on four locations, but without the time consuming thermal testing[60] because the QST battery should be concise and not an extensive burden to the patient. Therefore, a QST protocol limited to 60 minutes has been chosen. In addition, the current study applies a range of deep pressure stimuli (PPT), since pressure stimuli have been found as the most appropriate QST test to separate healthy subjects and patients with musculoskeletal pain.[61]

The study's advantage is that patients can keep their ongoing pain medication or stop it when the pain disappears. However, they are not allowed to change the doses or the type of medication. A limitation is that the order of the QST measures is not randomised for each patient and each visit. This might lead to order effects but this is done to minimize the carry-over effect of the CPM paradigm.

The results of this study will give insight into the effects of selective nerve root blocks on HACS, which are supposed to play a role in the presentation and complaints of patients with CLBPr. If a selective nerve root block affects HACS, it can help determine patients' treatment and give insight into HACS working mechanisms.

For peer review only - http://bmjopen.bmj.com/site/about/guidelines.xhtml

TRIAL STATUS

This trial is ongoing. The planned end date for the recruitment of patients and healthy volunteers is March 1, 2022

Authors Contributions

HT, GG, KP, LAN and AW designed the trial protocol. GG and AW secured funding for the study. IS, HT and AW drafted the manuscript. GG, LAN and KP contributed to the manuscript. All authors read and approved the final manuscript. IS, HT and AW are involved in data collection during the study. All authors are involved in the data analysis and manuscript preparations after the study is completed.

Competing interests statement

The authors declare that they have no competing interests.

Funding

The University of Groningen provided funding, University Medical Center Groningen, the Graduate Sçhool of Medicine and Department of Anesthesiology, Pain Center, Groningen, the Netherlands and the Ministry of Education of Brazil - Science without Borders programme (PhD scholarship to Ms Tharcila Chaves – project number 9140/13-1), Rio de Janeiro, Brazil. The funding bodies had no role in the study design and collection, analysis and interpretation of data, and writing of the manuscript.

For peer review only

For peer review only - http://bmjopen.bmj.com/site/about/guidelines.xhtml

REFERENCES

1. Spijker-Huiges A, Groenhof F, Winters JC, et al. Radiating low back pain in general practice: incidence, prevalence, diagnosis, and long-term clinical course of illness. *Scand J Prim Health Care* 2015;33(1):27-32. doi: 10.3109/02813432.2015.1006462 [published Online First: 2015/02/20]
2. Bogduk N, McGuirk B. Medical management of acute and chronic low back pain: an evidence-based approach. Amsterdam2002.
3. Wolff AP, Groen GJ, Wilder-Smith OH. Diagnosis of chronic radiating lower back pain without overt focal neurologic deficits: what is the value of segmental nerve blocks? *Therapy* 2005;2(4):577-85.
4. Wolff AP, Groen GJ, Crul BJ. Diagnostic lumbosacral segmental nerve blocks with local anesthetics: a prospective double-blind study on the variability and interpretation of segmental effects. *Reg Anesth Pain Med* 2001;26(2):147-55. doi: 10.1053/rapm.2001.21436 [published Online First: 2001/03/17]
5. Stolker RJ, Vervest ACM, Groen GJ. The management of chronic spinal pain by blockades: a review. *Pain* 1994;58(1):1-20.
6. Tajima T, Furamochi K, Kuramochi E. Selective Lumbosacral Radiculography and Block. *Spine* 1980;5(1):68-77.
7. Krempen JF, Smith BS. Nerve-root injection: a method for evaluating the etiology of sciatica. *JBJS* 1974;56(7):1435-44.
8. Herron LD. Selective nerve root block in patient selection for lumbar surgery: surgical results. *Journal of spinal disorders* 1989;2(2):75-79.
9. Dooley J, McBroom R, Taguchi T, et al. Nerve root infiltration in the diagnosis of radicular pain. *Spine* 1988;13(1):79-83.
10. Pelletier R, Higgins J, Bourbonnais D. Is neuroplasticity in the central nervous system the missing link to our understanding of chronic musculoskeletal disorders? *BMC Musculoskelet Disord* 2015;16:25. doi: 10.1186/s12891-015-0480-y [published Online First: 2015/04/19]
11. Arendt-Nielsen L, Morlion B, Perrot S, et al. Assessment and manifestation of central sensitisation across different chronic pain conditions. *Eur J Pain* 2018;22(2):216-41. doi: 10.1002/ejp.1140 [published Online First: 2017/11/07]
12. Müller M, Curatolo M, Limacher A, et al. Predicting transition from acute to chronic low back pain with quantitative sensory tests—a prospective cohort study in the primary care setting. *European journal of pain* 2019;23(5):894-907.
13. van Helmond N, Aarts HM, Timmerman H, et al. Is preoperative quantitative sensory testing related to persistent postsurgical pain? A systematic literature review. *Anesthesia & Analgesia* 2020;131(4):1146-55.
14. Petersen KK, Vaegter HB, Stubhaug A, et al. The predictive value of quantitative sensory testing: a systematic review on chronic postoperative pain and the analgesic effect of pharmacological therapies in patients with chronic pain. *Pain* 2021;162(1):31-44.
15. Bridges D, Thompson SW, Rice AS. Mechanisms of neuropathic pain. *Br J Anaesth* 2001;87(1):12-26. doi: 10.1093/bja/87.1.12 [published Online First: 2001/07/20]
16. Finco G, Evangelista M, Sardo S. Basic guide to chronic pain assessment: from neurophysiology to bedside. *Minerva Anestesiologica* 2020;86(12):1321-30.
17. Woolf CJ. Central sensitization: implications for the diagnosis and treatment of pain. *Pain* 2011;152(3 Suppl):S2-15. doi: 10.1016/j.pain.2010.09.030 [published Online First: 2010/10/22]
18. von Hehn CA, Baron R, Woolf CJ. Deconstructing the neuropathic pain phenotype to reveal neural mechanisms. *Neuron* 2012;73(4):638-52. doi: 10.1016/j.neuron.2012.02.008 [published Online First: 2012/03/01]

For peer review only - http://bmjopen.bmj.com/site/about/guidelines.xhtml

19. Konopka KH, Harbers M, Houghton A, et al. Bilateral sensory abnormalities in patients with unilateral neuropathic pain; a quantitative sensory testing (QST) study. *PLoS One* 2012;7(5):e37524. doi: 10.1371/journal.pone.0037524 [published Online First: 2012/05/26]
20. Rolke R, Magerl W, Campbell KA, et al. Quantitative sensory testing: a comprehensive protocol for clinical trials. *Eur J Pain* 2006;10(1):77-88. doi: 10.1016/j.ejpain.2005.02.003 [published Online First: 2005/11/18]
21. Mehta V, Snidvongs S, Ghai B, et al. Characterization of peripheral and central sensitization after dorsal root ganglion intervention in patients with unilateral lumbosacral radicular pain: a prospective pilot study. *Br J Anaesth* 2017;118(6):924-31. doi: 10.1093/bja/aex089 [published Online First: 2017/06/03]
22. Kregel J, Vuijk PJ, Descheemaeker F, et al. The Dutch Central Sensitization Inventory (CSI): Factor Analysis, Discriminative Power, and Test-Retest Reliability. *Clin J Pain* 2016;32(7):624-30. doi: 10.1097/AJP.0000000000000306 [published Online First: 2015/09/30]
23. Platform WHOICTR. WHO Trial Registration Data Set (Version 1.3) [Available from: http://awareness.who.int/ictrp/network/trds_v1.3/en/ accessed 5 January 2021.
24. Chan A-W, Tetzlaff JM, Altman DG, et al. SPIRIT 2013 statement: defining standard protocol items for clinical trials. *Annals of internal medicine* 2013;158(3):200-07.
25. Mayer TG, Neblett R, Cohen H, et al. The development and psychometric validation of the central sensitization inventory. *Pain Practice* 2012;12(4):276-85.
26. Sullivan MJ, Bishop SR, Pivik J. The pain catastrophizing scale: development and validation. *Psychological assessment* 1995;7(4):524.
27. Osman A, Barrios FX, Kopper BA, et al. Factor structure, reliability, and validity of the Pain Catastrophizing Scale. *Journal of behavioral medicine* 1997;20(6):589-605.
28. Crombez G, Eccleston C, Baeyens F, et al. When somatic information threatens, catastrophic thinking enhances attentional interference. *Pain* 1998;75(2-3):187-98.
29. Crombez G, Vlaeyen JW, Heuts PH, et al. Pain-related fear is more disabling than pain itself: evidence on the role of pain-related fear in chronic back pain disability. *Pain* 1999;80(1-2):329-39.
30. Van Damme S, Crombez G, Bijttebier P, et al. A confirmatory factor analysis of the Pain Catastrophizing Scale: invariant factor structure across clinical and non-clinical populations. *Pain* 2002;96(3):319-24.
31. McCracken LM. “Attention” to pain in persons with chronic pain: a behavioral approach. *Behavior therapy* 1997;28(2):271-84.
32. Roelofs J, Peters ML, McCracken L, et al. The pain vigilance and awareness questionnaire (PVAQ): further psychometric evaluation in fibromyalgia and other chronic pain syndromes. *Pain* 2003;101(3):299-306.
33. Van der Zee K, Sanderman R. RAND-36. *Groningen: Northern Centre for Health Care Research, University of Groningen, the Netherlands* 1993;28(6)
34. Hays RD, Sherbourne CD, Mazel RM. The rand 36-item health survey 1.0. *Health economics* 1993;2(3):217-27.
35. Pollard CA. Preliminary validity study of the pain disability index. *Perceptual and motor skills* 1984
36. Soer R, Köke AJ, Vroomen PC, et al. Extensive validation of the pain disability index in 3 groups of patients with musculoskeletal pain. *Spine* 2013;38(9):E562-E68.
37. Tait RC, Pollard CA, Margolis RB, et al. The Pain Disability Index: psychometric and validity data. *Arch Phys Med Rehabil* 1987;68(7):438-41.
38. Grönblad M, Hupli M, Wennerstrand P, et al. Intercorrelation and test-retest reliability of the Pain Disability Index (PDI) and the Oswestry Disability Questionnaire (ODQ) and their correlation with pain intensity in low back pain patients. *The Clinical journal of pain* 1993;9(3):189-95.
39. Ilmarinen J. The work ability index (WAI). *Occupational medicine* 2007;57(2):160-60.
40. Tuomi K, Ilmarinen J, Jahkola A, et al. Work ability index: Finnish Institute of Occupational Health Helsinki 1998.

For peer review only - http://bmjopen.bmj.com/site/about/guidelines.xhtml

41. Psychometric properties of Work Ability Index in the light of comparative survey study. International congress series; 2005. Elsevier.
42. De Zwart B, Frings-Dresen M, Van Duivenbooden J. Test–retest reliability of the Work Ability Index questionnaire. *Occupational medicine* 2002;52(4):177-81.
43. Hill JC, Whitehurst DG, Lewis M, et al. Comparison of stratified primary care management for low back pain with current best practice (STarT Back): a randomised controlled trial. *The Lancet* 2011;378(9802):1560-71.
44. Bier JD, Ostelo RW, van Hooff ML, et al. Validity and reproducibility of the STarT Back Tool (Dutch Version) in Patients With Low Back Pain in Primary Care Settings. *Physical Therapy* 2017;97(5):561-70.
45. Storm L, Rousing R, Andersen M, et al. Usefulness of the STarT Back Screening Tool to predict pain problems after lumbar spine surgery. *Dan Med J* 2018;65(12):1-4.
46. Backonja MM, Attal N, Baron R, et al. Value of quantitative sensory testing in neurological and pain disorders: NeuPSIG consensus. *Pain* 2013;154(9):1807-19. doi: 10.1016/j.pain.2013.05.047 [published Online First: 2013/06/08]
47. Weinstein S. Intensive and extensive aspects of tactile sensitivity as a function of body part, sex and laterality. *The skin senses* 1968
48. Fruhstorfer H, Gross W, Selbmann O. von Frey hairs: new materials for a new design. *European Journal of Pain* 2001;5(3):341-42.
49. Baumgärtner U, Magerl W, Klein T, et al. Neurogenic hyperalgesia versus painful hypoalgesia: two distinct mechanisms of neuropathic pain. *Pain* 2002;96(1-2):141-51.
50. LaMotte RH, Shain CN, Simone DA, et al. Neurogenic hyperalgesia: psychophysical studies of underlying mechanisms. *Journal of neurophysiology* 1991;66(1):190-211.
51. Ziegler E, Magerl W, Meyer R, et al. Secondary hyperalgesia to punctate mechanical stimuli: central sensitization to A-fibre nociceptor input. *Brain* 1999;122(12):2245-57.
52. Samuelsson M, Leffler A-S, Hansson P. Dynamic mechanical allodynia: on the relationship between temporo-spatial stimulus parameters and evoked pain in patients with peripheral neuropathy. *Pain* 2005;115(3):264-72.
53. Chan A, MacFarlane I, Bowsher D, et al. Weighted needle pinprick sensory thresholds: a simple test of sensory function in diabetic peripheral neuropathy. *Journal of Neurology, Neurosurgery & Psychiatry* 1992;55(1):56-59.
54. Greenspan JD, McGillis SL. Thresholds for the perception of pressure, sharpness, and mechanically evoked cutaneous pain: effects of laterality and repeated testing. *Somatosensory & motor research* 1994;11(4):311-17.
55. Hampf G, Bowsher D, Wells C, et al. Sensory and autonomic measurements in idiopathic trigeminal neuralgia before and after radiofrequency thermocoagulation: differentiation from some other causes of facial pain. *Pain* 1990;40(3):241-48.
56. Graven-Nielsen T, Wodehouse T, Langford RM, et al. Normalization of widespread hyperesthesia and facilitated spatial summation of deep-tissue pain in knee osteoarthritis patients after knee replacement. *Arthritis Rheum* 2012;64(9):2907-16. doi: 10.1002/art.34466 [published Online First: 2012/03/17]
57. Starkweather AR, Heineman A, Storey S, et al. Methods to measure peripheral and central sensitization using quantitative sensory testing: A focus on individuals with low back pain. *Appl Nurs Res* 2016;29:237-41. doi: 10.1016/j.apnr.2015.03.013 [published Online First: 2016/02/10]
58. Shanthanna H, Chan P, McChesney J, et al. Assessing the effectiveness of 'pulse radiofrequency treatment of dorsal root ganglion'in patients with chronic lumbar radicular pain: study protocol for a randomized control trial. *Trials* 2012;13(1):1-10.
59. Rolke R, Baron R, Maier Ca, et al. Quantitative sensory testing in the German Research Network on Neuropathic Pain (DFNS): standardized protocol and reference values. *Pain* 2006;123(3):231-43.

For peer review only - http://bmjopen.bmj.com/site/about/guidelines.xhtml

60. Starkweather AR, Heineman A, Storey S, et al. Methods to measure peripheral and central sensitization using quantitative sensory testing: A focus on individuals with low back pain. *Applied Nursing Research* 2016;29:237-41.

61. Neziri AY, Limacher A, Jüni P, et al. Ranking of tests for pain hypersensitivity according to their discriminative ability in chronic neck pain. *Regional Anesthesia & Pain Medicine* 2013;38(4):308-20.

For peer review only

For peer review only - http://bmjopen.bmj.com/site/about/guidelines.xhtml

TABLES

Table 1: Criteria for the inclusion and exclusion of patients

Inclusion criteria	Exclusion criteria
• Male and female patients<br>• Age 18 years or older<br>• Presence of chronic low back pain radiating in the leg<br>• Leg pain more or equal to back pain<br>• A physician must consider therapeutic sensory nerve root blocking or pulsed radiofrequency as an appropriate treatment intervention<br>• Agreement and signature of the informed consent	• Exclusion criteria for selective nerve blocks, according to local protocol<br>• Not or not sufficient understanding of the Dutch language<br>• Incapacity to follow instructions<br>• Mental incompetence to provide informed consent<br>• Chronic low back pain with radiation to both legs<br>• Pain in one (or more) sites where quantitative sensory testing will be applied except for the most painful point in the painful dermatome

For peer review only

For peer review only - http://bmjopen.bmj.com/site/about/guidelines.xhtml

Table 2: Criteria for the inclusion and exclusion of healthy **volunteers**

Inclusion criteria	Exclusion criteria
• Male and female healthy volunteers<br>• Age 18 years or older<br>• No history of low back pain<br>• Agreement and signature of the consent	• Not matching with one of the included patients based on sex and age (plus or minus three years of age)<br>• Not or not sufficient understanding of the Dutch language<br>• Incapacity to follow instructions<br>• Mental incompetence to provide informed consent

For peer review only

Table 3: Overview of the variables collected and interventions per visit

	Patients					Healthy volunteers
Demographics	Age, gender, weight, height, co-morbidities, pain medication, ethnic background and education.					
		V1a	V1b, V1c	V2	V3	HV
Time between visits		-	One week	One week	Four weeks	-
Questionnaires	CSI	✓	✓	✓	✓	✓
	SBST	✓	-	-	✓	✓
	RAND-36	✓	-	-	✓	✓
	PDI	✓	-	-	✓	✓
	WAI	✓	-	-	✓	✓
	PVAQ	✓	-	-	✓	✓
	PCS	✓	-	-	✓	✓
	Pain Drawing	✓	-	-	✓	-
	NRS pain	✓	✓	✓	✓	✓
QST	MDT	✓	✓	✓	✓	✓
	DMA	✓	✓	✓	✓	✓
	MPT	✓	✓	✓	✓	✓
	WUR	✓	✓	✓	✓	✓
	PPT	✓	✓	✓	✓	✓
	Cuff PPT	✓	✓	✓	✓	✓
	Cuff TS	✓	✓	✓	✓	✓
	CPM	✓	✓	✓	✓	✓
Intervention	dSNRB	✓	✓	-	-	-
	tSNRB or pRF	-	-	✓	-	-

Legend: V: visit, HV: healthy volunteers, QST: quantitative sensory testing; MDT: mechanical detection threshold; DMA: dynamic mechanical allodynia; MPT: mechanical pain threshold; WUR: Wind-up ratio; PPT: pressure pain threshold; Cuff PPT: pressure pain threshold by cuff algometer; Cuff TS: temporal summation by cuff algometer; CPM: conditioned pain

For peer review only

For peer review only - http://bmjopen.bmj.com/site/about/guidelines.xhtml

modulation; CSI: Central Sensitisation Inventory; SBST: STarT Back Screening Tool; RAND-36: RAND 36-Item Health Survey; PDI: Pain Disability Index; WAI: Work Ability Index; PVAQ: Pain Vigilance and Awareness Questionnaire; PCS: Pain Catastrophising Scale; Pain Drawing: Drawing in standard leg images. NRS-pain: Numeric Rating Scale for Pain; dSNRB: diagnostic selective nerve root block, tSNRB: therapeutic selective nerve root block, pRF: pulsed radiofrequency

For peer review only

For peer review only - http://bmjopen.bmj.com/site/about/guidelines.xhtml

Table 4: QST measurement location sequence

Visit	Sequence
Visit 1a	Location D-A-C-B
Visit 1b	Location D-C-A-B
Visit 1c	Location D-B-A-C
Visit 2	Location D-C-B-A
Visit 3	Location D-B-C-A

For peer review only

For peer review only - http://bmjopen.bmj.com/site/about/guidelines.xhtml

Table 5: QST measurement locations:

Location A	The most painful point in the painful dermatome
Location B	Contralateral point of location A
Location C	Control site 1 (contralateral of location A, distant from the painful area), between the scapula bones. On the pars medialis of the m. Trapezius at the height of the spina scapulae, 4cm lateral of the processus spinosus of the 3rd thoracic vertebra (Th3)
Location D	Control site 2 (distant from the painful area): contralateral to location A, at the deltoid muscle: On the pars medialis of the musculus Deltoideus, 3cm below the acromion
Location E	On the rectus femoris of the m. Quadriceps. Measures are performed on the musculus rectus Femoris 15 cm above the top edge of the patella.
Location F	On the lower leg. At the level of the largest circumference of gastrocnemius muscle.

For peer review only

For peer review only - http://bmjopen.bmj.com/site/about/guidelines.xhtml

Figure 1: Flowchart of the study procedure

Legend: QST: Quantitative sensory testing; dSNRB: diagnostic selective nerve root block; tSNRB: therapeutic selective nerve root block; pRF: Pulsed radiofrequency.

For peer review only

For peer review only - http://bmjopen.bmj.com/site/about/guidelines.xhtml

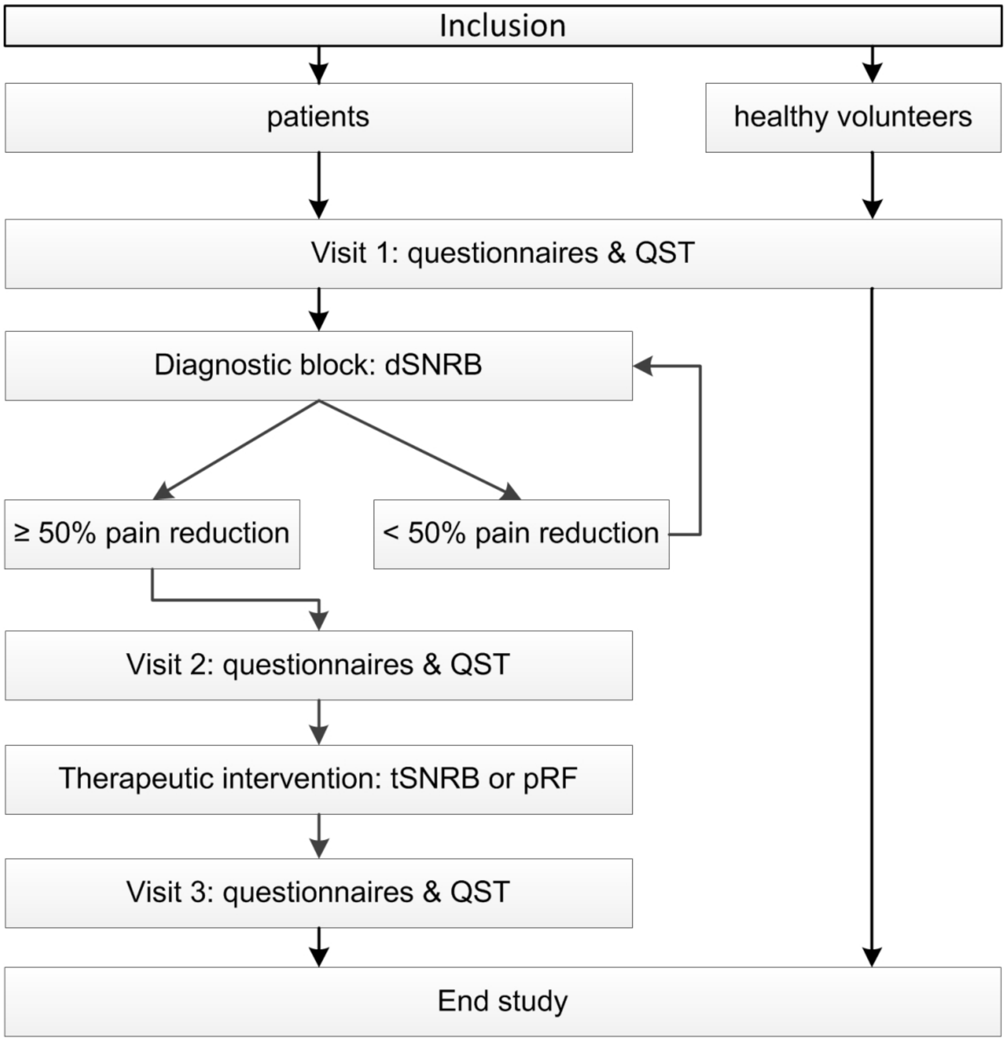

Figure 1: Flowchart of the study procedure / QST: Quantitative sensory testing; dSNRB: diagnostic selective nerve root block; tSNRB: therapeutic selective nerve root block; pRF: Pulsed radiofrequency.

119x124mm (300 x 300 DPI)

APPENDICES

Appendix A: Informed consent materials:

A model consent form and other related documentation is, in Dutch, given to participants and authorised surrogates

Bijlage B:

Toestemmingsformulier proefpersoon

Onderzoek naar de gevoeligheid van het centrale zenuwstelsel bij proefpersonen met chronische lage rugpijn uitstralend naar het been.

(Centrale sensitisatie in proefpersonen met chronische lage rugpijn uitstralend naar het been)

- Ik heb de informatiebrief gelezen. Ook kon ik vragen stellen. Mijn vragen zijn voldoende beantwoord. Ik had genoeg tijd om te beslissen of ik meedoe.
- Ik weet dat meedoen vrijwillig is. Ook weet ik dat ik op ieder moment kan beslissen om toch niet mee te doen of te stoppen met het onderzoek. Daarvoor hoef ik geen reden te geven.
- Ik weet dat sommige mensen mijn persoonsgegevens en/of gecodeerde onderzoeksgegevens kunnen inzien. Die mensen staan vermeld in deze informatiebrief.
- Ik geef toestemming om mijn gegevens nog 15 jaar na dit onderzoek te bewaren.
- Ik wil meedoen aan dit onderzoek.

Naam proefpersoon:

Handtekening: Datum : __ / __ / __

Ik verklaar dat ik deze proefpersoon volledig heb geïnformeerd over het genoemde onderzoek.

Als er tijdens het onderzoek informatie bekend wordt die de toestemming van de proefpersoon zou kunnen beïnvloeden, dan breng ik hem/haar daarvan tijdig op de hoogte.

Naam onderzoeker (of diens vertegenwoordiger):

Handtekening: Datum: __ / __ / __

<indien van toepassing>

Aanvullende informatie is gegeven door:

Naam:

Functie:

Handtekening: Datum: __ / __ / __

----

* Doorhalen wat niet van toepassing is.

De proefpersoon krijgt een volledige informatiebrief mee, samen met een kopie van het getekende toestemmingsformulier.

For peer review only - http://bmjopen.bmj.com/site/about/guidelines.xhtml

Appendix B: Intervention procedures

dSNRB

Diagnostic selective nerve root blocks (dSNRB) are performed in a fluoroscopy suite with monitoring equipment. At a minimum, pulse and blood pressure should be monitored every 1 to 5 minutes, depending on the procedure. Assisting personnel include nurses trained in advanced cardiac life support and a radiology technician. All procedures are performed with fluoroscopic guidance. Patient monitoring does not end after injecting medication. Post-procedure assessment is necessary because side effects or complications such as allergic reaction, vasovagal response, cardiac or respiratory depression, or high spinal block can occur. To ascertain whether the diagnostic selective nerve root block response was positive or negative, patients should perform activities or assume positions that invariably incite their pain. If a change in preprocedure and postprocedure verbal numeric rating scale rating of a minimum of 50% pain relief occurs, then the dSNRB result is considered positive.

For a lumbar dSNRB, the patient is placed in a prone position on the fluoroscopy table. The back is prepared and draped in a sterile manner. The level is located by establishing the lowest lumbar segment. The C-arm is rotated in the oblique position to allow access to the intervertebral foramen. Care must be maintained not to start too laterally to avoid renal or peritoneal puncture. A skin wheal is raised with 1% xylocaine. A 22-gauge, 6 or 10 cm with 5mm nonisolated tip needle is directed toward the foramen in the oblique position. Once the needle has penetrated the muscle adjacent to the spine, the needle then is advanced slowly in the AP plane to the 6-o'clock position of the pedicle and just inferior to the pedicle. The needle's position is in the safe triangle formed by the pedicle, the foramen's outer line, and the spinal nerve. Care is taken not to pierce the spinal nerve. Needle position is checked in the AP and lateral planes. One millilitre of non-ionic contrast agent is injected to outline the spinal nerve and dorsal root ganglion. The contrast pattern is evaluated to ensure that there is no vascular pattern, subarachnoid flow, or epidural spread to adjacent levels. One millilitre of 0.75% 0.3ml with Visipaque 320 mg I/ml 0.3ml (total 0.6ml) is infused.

Reference

Gauci, C. A. (2011). Manual of RF Techniques-a Practical Manual of Radiofrequency Procedures in Chronic Pain Management(3th Edition), CoMedical, 2011. Ridderkerk, the Netherlands.

Huston, C. W., & Slipman, C. W. (2002). Diagnostic selective nerve root blocks: indications and usefulness. *Physical medicine and rehabilitation clinics of North America*, 13(3), 545-565.

For peer review only - http://bmjopen.bmj.com/site/about/guidelines.xhtml

Perdok, J.M., Wolff, A.P., Stellema, R., Groen, G.J. (2017) 'Wortelblokkades'. Local protocol University Medical Center Groningen.

tSNRB

Therapeutic selective nerve root block (tSNRB) is similar to the dSNRB. The tSNRB is performed under sterile conditioned with an image intensifier. The target point was "safe triangle," i.e., above the exiting nerve root and below the corresponding pedicle. The skin is anaesthetised with 2-3 ml mepivacaine 2%. A spinal needle (22G) was inserted paramedian through the skin and muscles in a cranio-medial direction until a bony contact was encountered. This method allows advancing the needle in a safe triangle without contact with the nerve root. After verifying a correct needle positioning under biplanar image intensifier control, non-ionic contrast was injected until a radiculogram is obtained. Subsequently, 1ml 0.75% Bupivacaine and 1ml (40mg) Triamcinolon and 320mg I/ml Visipaque is injected. It should be stressed that this was a periradicular and not an intraneural injection. This technique is for the nerve roots L3-L5; for S1, a different technique is required. First, the image intensifier is positioned perpendicular to the foramen S1. A spinal needle is inserted perpendicular to the surface of the sacrum into the foramen. An image intensifier checks the correct needle positioning in two planes. After obtaining a correct periradiculargram, 1ml 0.75% Bupivacaine and 1ml (40mg) Triamcinolon and 320mg I/ml Visipaque is injected. The patients have clinical surveillance on the ward for about 30 minutes to account for unexpected side effects.

Reference

Bogduk N, Aprill C, Derby R, Selective nerve root blocks. In: Wilson DJ, ed. Interventional Radiology of the Muskuloskeletal System. London: Edward Arnold; 1995. p. 122–32.

Gauci, C. A. (2011). Manual of RF Techniques-a Practical Manual of Radiofrequency Procedures in Chronic Pain Management(3th Edition), CoMedical, 2011. Ridderkerk, the Netherlands.

Perdok, J.M., Wolff, A.P., Stellema, R., Groen, G.J. (2017) 'Wortelblokkades'. Local protocol University Medical Center Groningen.

Narozny, M., Zanetti, M., & Boos, N. (2001). Therapeutic efficacy of selective nerve root blocks in the treatment of lumbar radicular leg pain. *Swiss medical weekly*, *131*(0506).

pRF

For peer review only - http://bmjopen.bmj.com/site/about/guidelines.xhtml

Pulsed radiofrequency (pRF) is in a procedure similar to the tSNRB. The pRF is performed under sterile conditioned with an image intensifier. The target point was "safe triangle," i.e., above the exiting nerve root and below the corresponding pedicle. The skin is anaesthetised with 2-3 ml mepivacaine 2%. A spinal needle (22G) was inserted paramedian through the skin and muscles in a cranio-medial direction until a bony contact was encountered. This method allows advancing the needle in a safe triangle without contact with the nerve root. After verifying a correct needle positioning under biplanar image intensifier control, non-ionic contrast was injected until a radiculogram is obtained.

The tissue temperature is held at or below 42˚C on average. Radiofrequency is delivered in short, high-intensity bursts so that the radiofrequency electric field strength is increased without gross heating. The clinical objective is a neural modification by electric and thermal fields. This technique is for the nerve roots L3-L5; for S1, a different technique is required. First, the image intensifier is positioned perpendicular to the foramen S1. A spinal needle is inserted perpendicular to the surface of the sacrum into the foramen. An image intensifier checks the correct needle positioning in two planes. After obtaining a correct periradiculargram, the tissue temperature is held at or below 42˚C on average. Radiofrequency is delivered in short, high-intensity bursts so that the radiofrequency electric field strength is increased without gross heating. The patients have clinical surveillance on the ward for about 30 minutes to account for any unexpected side effects

Reference

Ewertowska, E., Mercadal, B., Muñoz, V., Ivorra, A., Trujillo, M., & Berjano, E. (2018). Effect of applied voltage, duration and repetition frequency of RF pulses for pain relief on temperature spikes and electrical field: a computer modelling study. *International Journal of Hyperthermia*, *34*(1), 112-121.

Gauci, C. A. (2011). Manual of RF Techniques-a Practical Manual of Radiofrequency Procedures in Chronic Pain Management(3th Edition), CoMedical, 2011. Ridderkerk, the Netherlands.

Perdok, J.M., Wolff, A.P., Stellema, R., Groen, G.J. (2017) ‘Wortelblokkades’. Local protocol University Medical Center Groningen.

For peer review only - http://bmjopen.bmj.com/site/about/guidelines.xhtml

Appendix C: Images drawing the most painful spot on the leg.

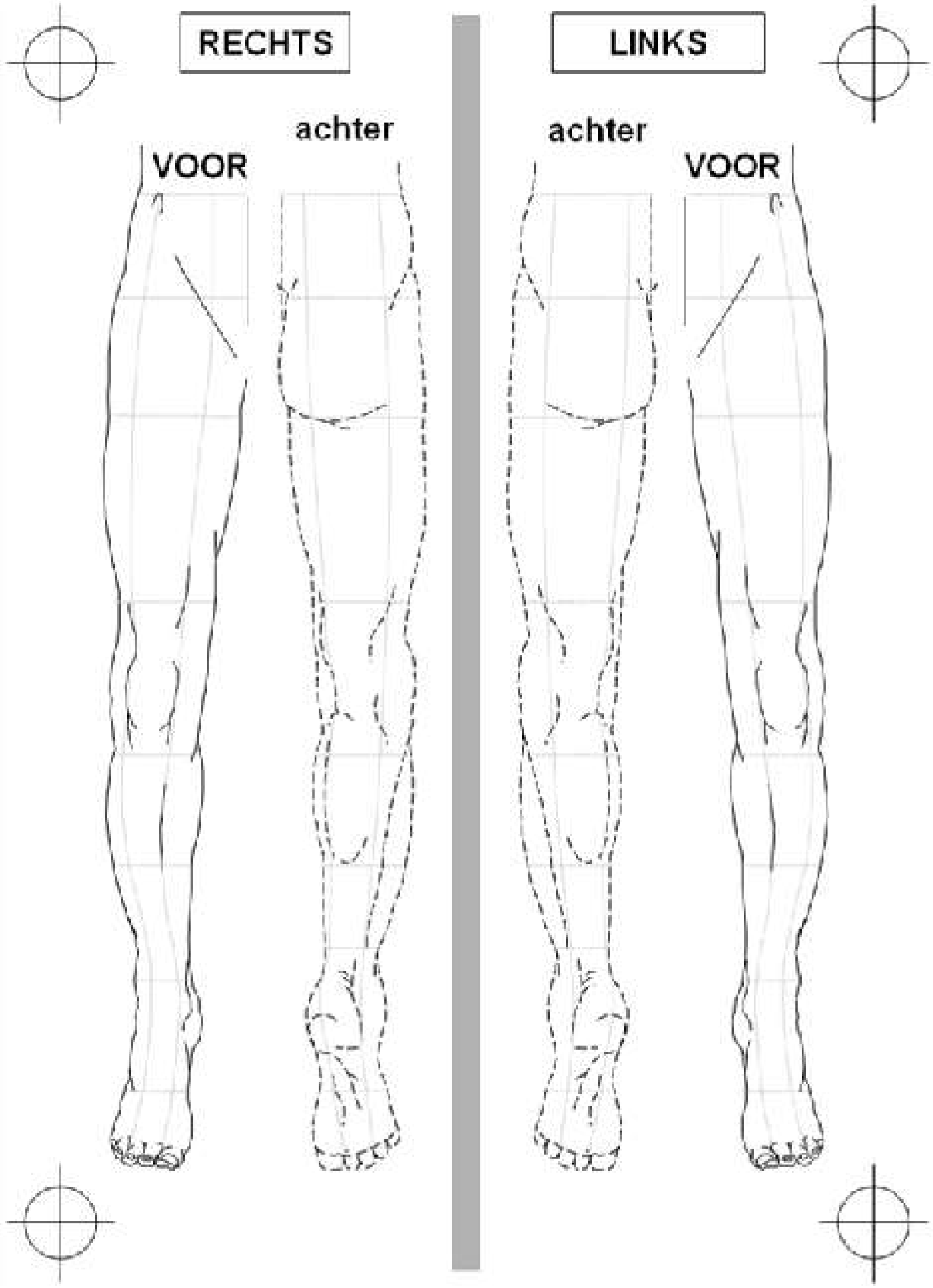

For peer review only - http://bmjopen.bmj.com/site/about/guidelines.xhtml

Reporting checklist for protocol of a clinical trial.

Based on the SPIRIT guidelines.

Instructions to authors

Complete this checklist by entering the page numbers from your manuscript where readers will find each of the items listed below.

Your article may not currently address all the items on the checklist. Please modify your text to include the missing information. If you are certain that an item does not apply, please write "n/a" and provide a short explanation.

Upload your completed checklist as an extra file when you submit to a journal.

In your methods section, say that you used the SPIRITreporting guidelines, and cite them as:

Chan A-W, Tetzlaff JM, Gøtzsche PC, Altman DG, Mann H, Berlin J, Dickersin K, Hróbjartsson A, Schulz KF, Parulekar WR, Krleža-Jerić K, Laupacis A, Moher D. SPIRIT 2013 Explanation and Elaboration: Guidance for protocols of clinical trials. BMJ. 2013;346:e7586

		Reporting Item	Page Number
**Administrative information**			
Title	#1	Descriptive title identifying the study design, population, interventions, and, if applicable, trial acronym	1
Trial registration	#2a	Trial identifier and registry name. If not yet registered, name of intended registry	10
Trial registration: data set	#2b	All items from the World Health Organization Trial Registration Data Set	10
Protocol version	#3	Date and version identifier	18
Funding	#4	Sources and types of financial, material, and other support	20
Roles and responsibilities: contributorship	#5a	Names, affiliations, and roles of protocol contributors	20

For peer review only

Roles and responsibilities: sponsor contact information	#5b	Name and contact information for the trial sponsor	n/a
Roles and responsibilities: sponsor and funder	#5c	Role of study sponsor and funders, if any, in study design; collection, management, analysis, and interpretation of data; writing of the report; and the decision to submit the report for publication, including whether they will have ultimate authority over any of these activities	20
Roles and responsibilities: committees	#5d	Composition, roles, and responsibilities of the coordinating centre, steering committee, endpoint adjudication committee, data management team, and other individuals or groups overseeing the trial, if applicable (see Item 21a for data monitoring committee)	n/a
**Introduction**			
Background and rationale	#6a	Description of research question and justification for undertaking the trial, including summary of relevant studies (published and unpublished) examining benefits and harms for each intervention	8,9
Background and rationale: choice of comparators	#6b	Explanation for choice of comparators	8
Objectives	#7	Specific objectives or hypotheses	9
Trial design	#8	Description of trial design including type of trial (eg, parallel group, crossover, factorial, single group), allocation ratio, and framework (eg, superiority, equivalence, non-inferiority, exploratory)	9
**Methods: Participants, interventions, and outcomes**			
Study setting	#9	Description of study settings (eg, community clinic, academic hospital) and list of countries where data will be collected. Reference to where list of study sites can be	10

For peer review only

		obtained	
Eligibility criteria	#10	Inclusion and exclusion criteria for participants. If applicable, eligibility criteria for study centres and individuals who will perform the interventions (eg, surgeons, psychotherapists)	10
Interventions: description	#11a	Interventions for each group with sufficient detail to allow replication, including how and when they will be administered	11
Interventions: modifications	#11b	Criteria for discontinuing or modifying allocated interventions for a given trial participant (eg, drug dose change in response to harms, participant request, or improving / worsening disease)	11,17
Interventions: adherance	#11c	Strategies to improve adherence to intervention protocols, and any procedures for monitoring adherence (eg, drug tablet return; laboratory tests)	11
Interventions: concomitant care	#11d	Relevant concomitant care and interventions that are permitted or prohibited during the trial	11
Outcomes	#12	Primary, secondary, and other outcomes, including the specific measurement variable (eg, systolic blood pressure), analysis metric (eg, change from baseline, final value, time to event), method of aggregation (eg, median, proportion), and time point for each outcome. Explanation of the clinical relevance of chosen efficacy and harm outcomes is strongly recommended	12-16
Participant timeline	#13	Time schedule of enrolment, interventions (including any run-ins and washouts), assessments, and visits for participants. A schematic diagram is highly recommended (see Figure)	11.12
Sample size	#14	Estimated number of participants needed to achieve study objectives and how it was determined, including clinical and statistical assumptions supporting any sample size calculations	16
Recruitment	#15	Strategies for achieving adequate participant enrolment to reach target sample size	11

For peer review only

**Methods: Assignment of interventions (for controlled trials)**			
Allocation: sequence generation	#16a	Method of generating the allocation sequence (eg, computer-generated random numbers), and list of any factors for stratification. To reduce predictability of a random sequence, details of any planned restriction (eg, blocking) should be provided in a separate document that is unavailable to those who enrol participants or assign interventions	n/a
Allocation concealment mechanism	#16b	Mechanism of implementing the allocation sequence (eg, central telephone; sequentially numbered, opaque, sealed envelopes), describing any steps to conceal the sequence until interventions are assigned	n/a
Allocation: implementation	#16c	Who will generate the allocation sequence, who will enrol participants, and who will assign participants to interventions	n/a
Blinding (masking)	#17a	Who will be blinded after assignment to interventions (eg, trial participants, care providers, outcome assessors, data analysts), and how	n/a
Blinding (masking): emergency unblinding	#17b	If blinded, circumstances under which unblinding is permissible, and procedure for revealing a participant's allocated intervention during the trial	n/a
**Methods: Data collection, management, and analysis**			
Data collection plan	#18a	Plans for assessment and collection of outcome, baseline, and other trial data, including any related processes to promote data quality (eg, duplicate measurements, training of assessors) and a description of study instruments (eg, questionnaires, laboratory tests) along with their reliability and validity, if known. Reference to where data collection forms can be found, if not in the	12-17

For peer review only

		protocol	
Data collection plan: retention	#18b	Plans to promote participant retention and complete follow-up, including list of any outcome data to be collected for participants who discontinue or deviate from intervention protocols	n/a
Data management	#19	Plans for data entry, coding, security, and storage, including any related processes to promote data quality (eg, double data entry; range checks for data values). Reference to where details of data management procedures can be found, if not in the protocol	16,17
Statistics: outcomes	#20a	Statistical methods for analysing primary and secondary outcomes. Reference to where other details of the statistical analysis plan can be found, if not in the protocol	17
Statistics: additional analyses	#20b	Methods for any additional analyses (eg, subgroup and adjusted analyses)	17
Statistics: analysis population and missing data	#20c	Definition of analysis population relating to protocol non-adherence (eg, as randomised analysis), and any statistical methods to handle missing data (eg, multiple imputation)	17
**Methods: Monitoring**			
Data monitoring: formal committee	#21a	Composition of data monitoring committee (DMC); summary of its role and reporting structure; statement of whether it is independent from the sponsor and competing interests; and reference to where further details about its charter can be found, if not in the protocol. Alternatively, an explanation of why a DMC is not needed	17
Data monitoring: interim analysis	#21b	Description of any interim analyses and stopping guidelines, including who will have access to these interim results and make the final decision to terminate the trial	17
Harms	#22	Plans for collecting, assessing, reporting, and managing solicited and spontaneously reported adverse events and other unintended effects of trial interventions or trial conduct	17
Auditing	#23	Frequency and procedures for auditing trial conduct, if	17

For peer review only

		any, and whether the process will be independent from investigators and the sponsor	
**Ethics and dissemination**			
Research ethics approval	#24	Plans for seeking research ethics committee / institutional review board (REC / IRB) approval	2
Protocol amendments	#25	Plans for communicating important protocol modifications (eg, changes to eligibility criteria, outcomes, analyses) to relevant parties (eg, investigators, REC / IRBs, trial participants, trial registries, journals, regulators)	18
Consent or assent	#26a	Who will obtain informed consent or assent from potential trial participants or authorised surrogates, and how (see Item 32)	11
Consent or assent: ancillary studies	#26b	Additional consent provisions for collection and use of participant data and biological specimens in ancillary studies, if applicable	2
Confidentiality	#27	How personal information about potential and enrolled participants will be collected, shared, and maintained in order to protect confidentiality before, during, and after the trial	2,16,18
Declaration of interests	#28	Financial and other competing interests for principal investigators for the overall trial and each study site	18
Data access	#29	Statement of who will have access to the final trial dataset, and disclosure of contractual agreements that limit such access for investigators	2,18
Ancillary and post trial care	#30	Provisions, if any, for ancillary and post-trial care, and for compensation to those who suffer harm from trial participation	n/a
Dissemination policy: trial results	#31a	Plans for investigators and sponsor to communicate trial results to participants, healthcare professionals, the public, and other relevant groups (eg, via publication, reporting in results databases, or other data sharing arrangements), including any publication restrictions	2,18
Dissemination policy:	#31b	Authorship eligibility guidelines and any intended use of	2,18

For peer review only

authorship		professional writers	
Dissemination policy: reproducible research	#31c	Plans, if any, for granting public access to the full protocol, participant-level dataset, and statistical code	2,18
**Appendices**			
Informed consent materials	#32	Model consent form and other related documentation given to participants and authorised surrogates	A
Biological specimens	#33	Plans for collection, laboratory evaluation, and storage of biological specimens for genetic or molecular analysis in the current trial and for future use in ancillary studies, if applicable	n/a

The SPIRIT Explanation and Elaboration paper is distributed under the terms of the Creative Commons Attribution License CC-BY-NC. This checklist was completed on 25. April 2021 using https://www.goodreports.org/, a tool made by the EQUATOR Network in collaboration with Penelope.ai

For peer review only

BMJ Open

Human Assumed Central Sensitisation (HACS) in Patients with Chronic Low Back Pain Radiating to the Leg (CLaSSICO study):Protocol for a Prospective Observational Study.

Journal:	*BMJ Open*
Manuscript ID	bmjopen-2021-052703.R1
Article Type:	Protocol
Date Submitted by the Author:	19-Oct-2021
Complete List of Authors:	Schuttert, Ingrid; UMCG, Department of Anesthesiology, Pain Center<br>Timmerman, Hans; UMCG, Department of Anesthesiology, Pain Center<br>Groen, Gerbrand; UMCG, Department of Anesthesiology, Pain Center<br>Petersen, Kristian; Aalborg Universitet, Department of Health Science and Technology<br>Arendt-Nielsen, Lars; Aalborg Universitet, Department of Health Science and Technology<br>Wolff, Andre; Universitair Medisch Centrum Groningen, Department of Anesthesiology, Pain Center
**Primary Subject Heading**:	Anaesthesia
Secondary Subject Heading:	Research methods
Keywords:	Pain management < ANAESTHETICS, Back pain < ORTHOPAEDIC & TRAUMA SURGERY, Neurological pain < NEUROLOGY, PAIN MANAGEMENT

SCHOLARONE™
Manuscripts

Human Assumed Central Sensitisation (HACS) in Patients with Chronic Low Back Pain Radiating to the Leg (CLaSSICO study):

Protocol for a Prospective Observational Study.

AUTHORS

Ingrid Schuttert*, MSc, Department of Anesthesiology, Pain Center, University of Groningen, University Medical Center Groningen (UMCG), Groningen, the Netherlands. OrcID: 0000-0003-2164-4721

Hans Timmerman, PhD, Department of Anesthesiology, Pain Center, University of Groningen, University Medical Center Groningen (UMCG), Groningen, the Netherlands. OrcID: 0000-0002-6082-9043

Gerbrand J. Groen, MD, PhD, Department of Anesthesiology, Pain Center, University of Groningen, University Medical Center Groningen (UMCG), Groningen, the Netherlands. OrcID: 0000-0002-7199-0107

Kristian K. Petersen, PhD, SMI, Center for Neuroplasticity and Pain, Department of Health Science and Technology, Faculty of Medicine, Aalborg University, Aalborg, Denmark. ORCID: 0000-0003-4506-000X

Lars Arendt-Nielsen, PhD, SMI, Center for Neuroplasticity and Pain, Department of Health Science and Technology, Faculty of Medicine, Aalborg University, Aalborg, Denmark. ORCID: https://orcid.org/0000-0003-0892-1579

André P. Wolff, MD, PhD, Department of Anesthesiology, Pain Center, University of Groningen, University Medical Center Groningen (UMCG), Groningen, the Netherlands. OrcID: 0000-0001-6240-3903

* Corresponding author for public and scientific queries:

Ingrid Schuttert
Department of Anesthesiology, UMCG Pain Center, University Medical Center Groningen (UMCG), PO Box 30002, 9751 ND Haren, the Netherlands.
Email: i.schuttert@umcg.nl
Tel: +31503612140

Word count: 4147(Word count, excluding title page, abstract, references, figures and tables)

For peer review only - http://bmjopen.bmj.com/site/about/guidelines.xhtml

DECLARATIONS

Ethics approval and consent to participate

The medical and ethical review board Committee on Research Involving Human Subjects of the University Medical Center Groningen, Groningen, the Netherlands, has approved the study "Central sensitisation in patients with chronic low back pain radiating to the leg", dossier number NL60439.042.17 on November 1, 2017. Due to COVID-19, an amendment was submitted and approved on August 11, 2020, to prolong the participants' inclusion date due to March 1, 2022. The participants will receive written and oral information about the study and all assessments. The participants will receive written and oral information about the study and assessments. Before the start of the study, written informed consent will be provided by all participants (see appendix A).

Consent for publication

Not applicable.

Availability of data and materials

Due to the data's sensitivity and the restrictions from informed consent, the data will not be stored in a public repository. The data and meta-data will be stored at a repository at the UMCG, ensuring the data's security and backup. UMCG pursues a FAIR data policy for research conducted in the UMCG. To make the data findable and accessible for others, we will include a description of the UMCG data catalogue data: https://www.groningendatacatalogus.nl/. The data's metadata will be made available for researchers inside and outside the institute via the catalogue. This catalogue is in sync with relevant (inter)national catalogues, such as Biobanking and Biomolecular Resources Research Infrastructure and National Academic Research and Collaborations Information System. A data access committee has been put in place to review requests and assure the accessibility of the data. This access committee can be reached via the corresponding author (Ingrid Schuttert, e-mail: i.schuttert@umcg.nl or André Wolff, e-mail: a.p.wolff@umcg.nl). The results will be published in peer-reviewed scientific journals and submitted for presentation at relevant national and international scientific meetings/conferences.

Acknowledgements

Not applicable.

Patient consent for publication

For peer review only

For peer review only - http://bmjopen.bmj.com/site/about/guidelines.xhtml

Not required.

Keywords

Chronic Low Back Pain; Sensitisation; Study protocol; Quantitative sensory testing (QST); Selective nerve root block (SNRB); Pulsed radiofrequency (PRF), Human Assumed Central Sensitisation (HACS).

For peer review only

For peer review only - http://bmjopen.bmj.com/site/about/guidelines.xhtml

ABBREVIATIONS

ANOVA:	ANalysis Of VAriance
CLBPr:	Chronic Low Back Pain radiating to the leg;
CSI:	Central Sensitisation Inventory;
dSNRB:	Diagnostic Selective Nerve Root Block
Dlv	Dutch language version
HACS	Human Assumed Central Sensitisation
IQR:	Interquartile range
NRS:	Numerical Rating Scale
pRF:	Pulsed Radiofrequency;
QST:	Quantitative Sensory Testing;
RAND-36:	Rand 36-Item Health Survey
SBST:	STarT Back Screening Tool
tSNRB:	Therapeutic Selective Nerve Root Block

For peer review only

For peer review only - http://bmjopen.bmj.com/site/about/guidelines.xhtml

ABSTRACT

Introduction: Patients with chronic low back pain radiating to the leg (CLBPr) are sometimes referred to a specialised pain clinic for a precise diagnosis based, for example, on a diagnostic selective nerve root block. Possible interventions are therapeutic selective nerve root block or pulsed radiofrequency. Central pain sensitisation is not directly assessable in humans and therefore the term 'human assumed central sensitisation' (HACS) is proposed. The possible existence and degree of sensitisation associated with pain mechanisms assumed present in the human central nervous system, its role in the chronification of pain and its interaction with diagnostic and therapeutic interventions are largely unknown in patients with CLBPr. The aim of quantitative sensory testing (QST) is to estimate quantitatively the presence of HACS and accumulating evidence suggest that a subset of patients with CLBPr have facilitated responses to a range of QST tests.
The aims of this study are to identify HACS in patients with CLBPr, to determine associations with the effect of selective nerve root blocks and compare outcomes of HACS in patients to healthy volunteers.

Methods and analysis: A prospective observational study including fifty patients with CLBPr. Measurements are performed before diagnostic and therapeutic nerve root block interventions and at four weeks follow-up. Data from patients will be compared with those of fifty sex- and age-matched healthy volunteers. The primary study parameters are the outcomes of QST and the central sensitisation inventory. Statistical analyses to be performed will be analysis of variance.

Ethics and dissemination: The Medical Research Ethics Committee of the University Medical Center Groningen, Groningen, the Netherlands, approved this study (dossier NL60439.042.17). The results will be disseminated via publications in peer-reviewed journals and at conferences.

Trial registration number: NTR NL6765

For peer review only - http://bmjopen.bmj.com/site/about/guidelines.xhtml

Article Summary

STRENGTHS AND LIMITATIONS OF THIS STUDY

- Strength: comparison with sex- and age-matched healthy controls.
- Strength: Multimodal assessment of human assumed central and peripheral sensitization via objective and subjective measures.
- Limitation: The order of the quantitative sensory testing measures is not randomised for each patient and each visit and is only randomised per visit.

For peer review only

For peer review only - http://bmjopen.bmj.com/site/about/guidelines.xhtml

INTRODUCTION

Background:

The mean incidence and prevalence of chronic low back pain radiating to the leg (CLBPr) are 9.4 and 17.2 per 1000 person-years respectively.[1] If the diagnosis of CLBPr is unclear despite extensive neurological, orthopaedic and radiological examination, patients are sometimes referred to a specialised multidisciplinary pain clinic, where a diagnostic selective nerve root block (dSNRB) might provide a more precise diagnosis.[2 3] This block, achieved by injection of a local anaesthetic, can help determine the predominant segmental level of the pain.[4-9] A positive dSNRB is generally followed by an injection of local anaesthetics and corticosteroids, i.e. a therapeutic SNRB (tSNRB) or a pulsed radiofrequency treatment (pRF) of the spinal nerve root, its dorsal ganglion or a combination thereof.

Growing evidence suggests that central pain mechanisms are facilitated in a subset of patients with CLBPr.[10 11] Facilitation of peripheral and central pain mechanisms have been suggested to be associated with pain progression, poor recovery, poor response to pharmacological interventions and inadequate response to low back surgery, indicating the clinical need of these assessments.[12-14] Preclinical data suggests that nerve fibres can malfunction and respond as nociceptors, resulting in pain from light stimuli, possibly caused by peripheral and central sensitisation.[15] Dysfunction of the somatosensory system may clinically lead to positive sensory symptoms (e.g. allodynia, aftersensations, enhanced temporal summation) and negative sensory symptoms (e.g. hypoesthesia).[11 16-19] Because mechanisms related to central sensitisation can currently only be assessed in animals,[11] proxies are used in humans to assess clinical signs that may be associated with the presence of central pain mechanisms. We use the term ‘human assumed central sensitisation’ (HACS) because there is no gold standard to estimate the presence of central sensitisation in humans at present. Moreover, there is also no clear definition, method or (clinical) guideline applicable to diagnose central sensitisation in humans.

The role of HACS in the development of CLBPr, in the chronification of pain, and the interaction with diagnostic and therapeutical interventions is not clear.[11] Several methods are thought to assist in the clinical diagnosis of HACS.[11] With Quantitative Sensory Testing (QST) we measure the responses to calibrated graded innocuous or noxious stimuli [20] and evaluate the presence of HACS in patients with CLBPr.[21] Mehta et al. showed that following pRF treatment patients with CLBPr have increased pressure pain thresholds and increased conditioned pain modulation, suggesting a normalization of the sensory pain profile.[21] The presence of HACS might also be evaluated with the Central Sensitisation Inventory (CSI),[22]

For peer review only - http://bmjopen.bmj.com/site/about/guidelines.xhtml

but there are no clinical associations established between the CSI and experimental measures.[11]

Objectives:

The primary objective of this study is to determine if therapeutic selective nerve root block or pulsed radiofrequency in patients with chronic low back pain radiating to one leg changes the outcome of the CSI and the QST measures as proxies for human assumed central sensitisation. Secondary objectives are (1) to identify human assumed central sensitisation based on the outcome of the CSI in patients with chronic low back pain radiating to one leg, (2) to determine if a diagnostic selective nerve root block changes the outcome of the CSI and the QST measures as proxies for human assumed central sensitisation in patients with chronic low back pain radiating to one leg, and (3) to compare the differences in the outcome of the CSI and QST measures as proxies for human assumed central sensitisation between patients (before and after selective nerve root blocks) and matched healthy volunteers.

Trial design:

A prospective longitudinal observational study about HACS in patients with CLBPr who undergo selective nerve root blocks compared to sex- and age-matched healthy volunteers.

For peer review only - http://bmjopen.bmj.com/site/about/guidelines.xhtml

METHODS

This study was registered prospectively in the Netherlands Trial Register: Trial NL6765 (first posted January 10, 2018). The trial information in this protocol is in line with the WHO Trial Registration Data Set [23] and written following the SPIRIT 2013 Guideline.[24]

Participants, Interventions and outcomes

Study setting:

Patient recruitment, treatment, and measurements take place in the multidisciplinary academic pain center, University Medical Center Groningen (UMCG), Groningen, the Netherlands.

Patient and public involvement:

Patients were not involved in the development of the research question and the determination of the outcome measures. During recruitment, patients are asked to suggest healthy volunteers whose age and gender match their own to participate in the study.

Eligibility criteria:

Fifty consecutive adult patients of both sexes with chronic low back pain radiating in the dermatomes L3 to S2 and fifty sex- and age-matched healthy volunteers will be included. The inclusion and exclusion criteria are summarised in table 1 (Table 1). The inclusion criteria for the healthy volunteers are shown in table 2 (Table 2).

Patients can retain their ongoing pain medication or discontinue it if they stop feeling pain during the study. Other changes in medication use are not allowed during participation. Moreover, all other pain interventions (e.g. physical therapy, transcutaneous electrical nerve stimulation) during their participation in this study are prohibited. Healthy volunteers are matched with the patients on sex and age (plus or minus three years of age). As mentioned, patients are asked to suggest suitable healthy volunteers. If necessary healthy volunteers are also recruited via flyers placed near to the UMCG facilities. Healthy volunteers do not undergo selective nerve root blocks. Patients and healthy volunteers may withdraw from the study at any time for any reason if they wish to do so and without any consequences. The investigator can decide to withdraw a participant from the study for urgent medical reasons.

For peer review only - http://bmjopen.bmj.com/site/about/guidelines.xhtml

Interventions:

The intervention provided in this study is performed in the same way as the standard intervention provided in the multidisciplinary academic pain center and follows the guideline for SNRB (see appendix B for the detailed procedure). Table 3 shows which intervention is used in each visit (Table 3).

Participant timeline:

Patients and healthy volunteers receive a verbal and written explanation of the study procedures and are given sufficient time to consider their participation. After written informed consent is provided, a meeting is scheduled. The flowchart of the study is shown in figure 1 (Figure 1). The schedule for the patient and the healthy volunteer assessments are shown in table 3 (Table 3). Visit 1b, and 1c are only performed for the patients when more than one dSNRB is necessary. After the dSNRB, numerical rating scale (NRS) for pain in the back as well as in the leg are assessed. In case of a pain reduction of ≥ 50%, the patient is considered to have a "positive result", and the therapeutic intervention is planned. The responsible physician will decide which intervention (tSNRB or pRF treatment) each patient will receive (which is part of care as usual). If a pain reduction is < 50%, the patient is considered to have a "negative result". With a negative result, the patient will undergo another dSNRB at a different spinal level (3 dSNRBs are the maximum within this study). dSRBs, tSNRBs and pRF will be performed according to the standard procedures of the multidisciplinary academic pain center. Healthy volunteers are not asked to draw their pain area and do not receive any diagnostic or therapeutic intervention. For healthy volunteers, only baseline assessment is performed.

Recruitment:

At the multidisciplinary academic pain center, on average, two new patients with CLBPr are seen per week. If two patients per month agree to join the study, the sample will be completed within four years. Because patients must visit the multidisciplinary academic pain center once more than during care as usual (visit 3), they will be compensated for this visit when it is not possible to schedule it with another regular hospital visit. Patients will receive compensation for extra travel costs (€0.19/km). Healthy volunteers will receive 10 euros for their visit plus compensation for travel costs (€0.19/km).

Duration

Each visit will last no longer than two and a half hours, consisting of 1 hour measurements (patients and healthy volunteers), 0.5 hour for the treatment and 0.5-1 hour for the

For peer review only - http://bmjopen.bmj.com/site/about/guidelines.xhtml

assessment of the effect of the treatment (patients). Interventions are performed one week apart, and visit 3 will be four weeks after the therapeutic intervention. Depending on the number of dSNRBs performed, the total duration of this study for the patient will take between five to seven weeks (3-5 visits). Healthy volunteers will undergo only one visit with an assessment of 1 hour.

The first patient was included on June 5, 2018. the planned end date for the inclusion of participants is March 1, 2022.

Outcomes:

All measurements are performed for study-specific reasons. The primary outcomes are QST measures and the CSI. Secondary outcomes are Pain Catastrophising Scale, Pain Vigilance and Awareness Questionnaire, 36-Item Short-Form Health Survey (SF-36), Pain Disability Index, Work Ability Index, STarT Back Screening Tool (SBST), Numeric Rating Scale (NRS) and drawing in standard leg images (pain drawing) (Table 3).

Descriptive data:

Descriptive items include: age (years), sex (male /female), weight (kg), height (cm), education, comorbidities, medication and ethnic background.

Questionnaires:

One or two days before each visit, the patient receives an e-mail with a link and is asked to fill out the questionnaires (Table 3). The questionnaires (provided in Dutch) are sent through RoQua (UMCG, Groningen, The Netherlands), a questionnaire program built into the electronic patient's file. The whole set of questionnaires is sent for visit 1a and 3, and it takes about 20 minutes to complete. For visit 1b-1c and 2, only the CSI is sent, and take about 5 minutes to complete. Patients who cannot fill out the questionnaires online will receive them on paper at the beginning of the visit.

Central Sensitisation Inventory (CSI):[22 25] The CSI contains 25 statements related to current health symptoms. Each item is measured on a 5-point Likert scale from 0 (never) to 4 (always). A cut-off score of 40 is being used.[22 25] The original CSI (Cronbach's alpha = 0.879, test-retest reliability = 0.817)[25] as well as the CSI-Dutch language version (Dlv) (Cronbach's alpha = 0.91, test-retest reliability = 0.88 and 0.91)[22] shows a good internal consistency.

Pain Catastrophizing Scale:[26] It assesses thoughts and feelings about pain. For each of 13 statements the participant is asked to answer on a scale from 0 (totally not) to 4 (always). The original pain catastrophizing scale (Cronbach's alpha = 0.93,[27] test-retest reliability =

For peer review only - http://bmjopen.bmj.com/site/about/guidelines.xhtml

0.75)[26] as well as the Pain catastrophizing scale-Dlv (Cronbach's alpha between 0.85 and 0.91)[28-30] show good internal consistency.

Pain Vigilance and Awareness Questionnaire.[31 32] This questionnaire evaluates the awareness of pain. The participant can assign a number from 0 (never) to 5 (continuously) for 16 statements. The original pain vigilance and awareness questionnaire shows good internal consistency (Cronbach's alpha=0.86)[31] and adequate test-retest reliability (r=0.80).[31] The Pain vigilance and awareness questionnaire-Dlv also shows good internal consistency (Cronbach's alpha = 0.87).[32]

RAND 36-item Health Survey (RAND-36):[33 34] The RAND-36 is a participant-reported survey of health-related quality of life. It consists of 8 sections: vitality, physical functioning, bodily pain, general health perceptions, physical role functioning, emotional role functioning, social role functioning and mental health. The RAND-36-Dlv has shown to be valid and reliable.[33]

Pain Disability Index:[35 36] This questionnaire is used to measure the degree to which aspects of the patient's life is disrupted by chronic pain. It lists seven life activity categories (family/home responsibilities, recreation, social activity, occupation, sexual behaviour, self-care and life-support activities). The original pain disability index shows good internal consistency (Cronbach's alpha=0.871),[37] and the test-retest reliability is good (ICC=0.91).[38] The pain disability index-Dlv showed good internal consistency with patients with chronic low back pain (Cronbach's alpha= 0.85)[36] and sufficient test-retest reliability (ICC=0.78).[36]

Work Ability Index:[39] It is used to assess workability during health examinations. The index is determined based on the answers to a series of questions that consider the demands of work, the worker's health status and resources. The original work ability index has been validated (Cronbach's alpha = 0.72).[40 41] The work ability index-Dlv showed sufficient internal consistency (Cronbach's alpha = 0.72)[41] and an acceptable test-retest reliability.[42]

STarT Back Screening Tool (SBST):[43 44] The SBST is a 9-item patient self-reporting questionnaire validated for triage of patients with non-specific low back pain in primary care. It has shown to be helpful in tertiary care.[45] The SBST identifies modifiable prognostic factors from the health domains of pain, activity limitation and psychosocial factors, which are risk factors for persistent non-specific low back pain. The SBST classifies patients into three groups: low, medium or high risk of poor prognosis based on the symptom complexity. When assessed by general practitioners, this classification can assist in making decisions towards appropriate evidence-based treatment pathways. The SBST-Dlv showed good validity and an excellent to fair reproducibility.[44]

For peer review only - http://bmjopen.bmj.com/site/about/guidelines.xhtml

Numeric Rating Scale (NRS) for Pain: A pain rating scale where patients are asked to rate their pain at two locations (leg and back), giving a number between 0 and 10 (0 = no pain/10 = maximum pain imaginable). In the QST measurements, an NRS is also used with a scale from 0 to 100 10 (0 = no pain/100 = maximum pain imaginable).

Drawing in standard leg images (Pain Drawing): The patient is asked to draw, on an image with standard legs (see appendix C), the area where they experience the most pain. When more locations are drawn, an arrow will indicate the most painful spot.

Quantitative Sensory Testing (QST):

QST is a psychophysical method that measures responses to calibrated graded innocuous or noxious stimuli in addition to bedside clinical examination of the somatosensory system.[19 46] The measurements are based on the QST battery developed by the German Research Network on Neuropathic Pain.[10 15] This protocol is extended with cuff algometry (NociTech, Aalborg, Denmark). QST is used for the analyses of HACS. The participants will be comfortable sitting and lying on a bed during all assessments when evaluating the stimuli. The sequences' order is counterbalanced and documented in the case report form (Table 4). The measurements take about 45-60 minutes.

Mechanical Detection Threshold: Mechanical detection threshold is assessed via von Frey-filaments (OptiHair2, MRC Systems GmbH, Heidelberg, Germany). When the 16mN von Frey filament's touch is felt, the von Frey filament's intensity is reduced until no touch is felt. The intensity then will be increased until the touch is felt again. This test will be repeated at least three times to detect the mechanical detection threshold. The first von Frey filament that cannot be felt is documented.[47-49]

Dynamic Mechanical Allodynia: Dynamic mechanical allodynia is tested using dynamic innocuous stimulus (soft brush; Sense Lab Brush no. 5, Somedic, Sösdala, Sweden). The innocuous stimulus is administered once and after that three times in a 1-2 cm wiping motion on the skin. The participant is asked to say if something was felt and to rate the NRS-pain from 0 to 100.[50-52]

Mechanical Pain Threshold: This test is performed with weighted pinprick stimuli (MRC Systems GmbH, Heidelberg, Germany) using the method of limits. Five threshold determinations are made, each with a series of ascending and descending stimulus intensities. The first pinprick that is considered painful is documented.[51 53-55]

Wind-up Ratio: In this test, the NRS-pain score (0-100) experienced for a single pinprick stimuli (256N) is compared to an applied series of repetitive (ten times with intervals of 1

For peer review only - http://bmjopen.bmj.com/site/about/guidelines.xhtml

second) pinprick stimuli of the same intensity (256N). The wind-up ratio is calculated by dividing the pain intensity rating for the series of stimuli by the pain intensity rating for the single stimulus.

Pressure Pain Threshold: Using a handheld pressure algometer (Wagner FDX 10, Greenwich, USA), the threshold for pressure-induced pain is assessed by slowly increasing stimulus intensities (5 N/s). The threshold is determined when the pressure becomes painful/more than just the feeling of pressure when the participants say 'now'. The NRS-pain (0-100) and the applied force (N) at that moment are documented.

Cuff algometry (Cuff): During cuff algometry, the participants will be instructed to continuously evaluate the pain using an electronic visual analogue scale (0 = no pain, 10 = worst imaginable pain).

Cuff pain tolerance threshold: The cuff pressure will be increased by one kPa/s on the dominant leg. The participant will be instructed to rate the pain intensity continuously on the electronic visual analogue scale until the tolerance level is reached, and the participant pushes the stop button. When the participant pushes this button, the pressure is released immediately. The pain tolerance threshold (PTT) is defined as when the participant presses the stop button. A similar assessment will be performed on the non-dominant leg after the cuff temporal summation.

Cuff temporal summation: A total of 10 repeated mechanical cuff pressure stimuli at the intensity of PTT will be delivered at 0.5 Hz (1-second stimulus duration and 1-second interval between stimuli) to the lower leg. A constant pressure of 1 kPa will be applied between the individual pressure stimuli sets to avoid movement of the cuff. The participants will continuously rate the pain intensity on the electronic visual analogue scale during the 10 repeated stimuli.

Conditioned Pain Modulation: Pressure pain threshold will be repeated on the deltoid muscle and the rectus femoris, both on the painful dermatome's contralateral side. The participant will be asked to put the dominant hand up to the wrist with spread fingers in a bucket filled with ice and cold water (conditioning stimulus). The amount of time (at a maximum of three minutes for safety reasons) that it takes until the participant removes the immersed hand out of the bucket will be noted. Immediately after, pressure pain threshold in the deltoid muscle and the rectus femoris both on the contralateral side of the painful dermatome will be repeated.[17 18 20]

For peer review only - http://bmjopen.bmj.com/site/about/guidelines.xhtml

Measurement sites: Measures will be taken at six different sites in total (Table 5). The QST measures will be performed on sites A-D. The conditioned pain modulation will be assessed at sites D and E. The cuff measures will be performed at site F.

Sample size:

The total sample size is 100 participants (50 patients and 50 healthy volunteers for comparison), which is consistent with the sample sizes of several studies that have used QST. For example, Graven-Nielsen et al.[56] applied a QST battery in 48 patients and 21 pain-free healthy controls to determine the importance of ongoing tissue pathology in maintaining enhanced central pain processing.[56] Starkweather et al.[57] included 44 patients and 20 healthy controls and used QST to identify HACS in patients with low back pain.[57] With a power of 80% and a type I error of 5%, the number of participants was calculated (G*power for Windows, Version 3.1.9.7, Heinrich Heine Universität, Dusseldorf, Germany) to be 50 per group, resulting in a medium effect size of 0.57. This medium effect size is hypothesised because multiple QST measurements are tested with independent variables such as different interventions and demographic variables.

Data collection, management, and analysis

Data collection and management:

The primary data (raw) will be collected on paper and entered into an electronic data capture: OpenClinica (OpenClinica, LLC, Waltham, USA, version 3.14). The questionnaires will be assessed via RoQua (RoQua, UMCG, Groningen, The Netherlands) as implemented in the electronic patient files (EPIC, Epic Systems Corporation, Verona, USA, version August 2020). For the healthy volunteers' dummy numbers (for the hospital information system) will be created to enable them to access and fill in the questionnaires. The data in Open Clinica and RoQua will be combined into SPSS (version 27, IBM, Armonk, USA) database.

The raw data on paper, in OpenClinica and the data of RoQua, will be anonymised and coded. The data will be stored at the UMCG research drive.

Data will be handled confidentially. A participant identification code list will be used to link the data to the participant. The principal investigator will safeguard the key to the code.

Data will be accessible for the team of investigators, the Medical Ethical Review Board and the health care Inspection. Data will be saved for 15 years. Data will be used for publication, but no participant will be traceable. The handling of personal data will comply with the Dutch Personal Data Protection Act.

For peer review only - http://bmjopen.bmj.com/site/about/guidelines.xhtml

Statistical methods:

Data records from the different tests and questionnaires will be collected and merged into one database. SPSS software version 27.0 or higher (IBM, Armonk, USA) will be used to perform the analyses. Descriptive statistics data will be presented as means ± standard deviation (SD) when normally distributed and as median and interquartile range (IQR) (25-75) when not normally distributed. A significance level of 0.05 is used in all analyses.

For the primary objective, to determine treatment effect of the tSNRB and pRF the difference between visit 1a and visit 3 will be analysed for nine primary measures (CSI and eight QST measures). The results will be checked for possible covariates. This analysis will be performed using an One-way ANOVA..

For secondary objective 1, to identify the presence of HACS in patients with CLBPr data from visit 1a will be used. The CSI cut-off value of 40 will be used[58] to identify the presence of HACS.

For secondary objective 2, the difference between the latest visit 1 and visit 2 will be used to determine treatment effect of the dSNRB. The nine primary measures (CSI and eight QST measures) will be used and checked for possible covariates for this analysis. The difference will be assessed using an One-way ANOVA.

For secondary objective 3, the differences between patients' visit 1a vs visit 3 and healthy volunteers are calculated for the questionnaires and QST measurements. The calculated differences will be assessed using an one-way ANOVA.

Monitoring

Data monitoring:

The monitoring will be carried out by research support staff based on GCP monitoring principles. The departmental research office will monitor the trial regularly. This typically includes the following checks:

1) The data collected are consistent with adherence to the trial protocol; 2) Case report files are completed by authorised persons; 3) No key data are missing; 4) The data appears to be valid (i.e. range and outlier checks); 5) Review of recruitment rates, withdrawals and losses to follow-up.

Harms:

For peer review only - http://bmjopen.bmj.com/site/about/guidelines.xhtml

Experienced specialised pain staff members perform all interventions in the care as usual setting. Study-related additional harms may consist of QST-related pain during and a short time after the measurements. No other harm is expected.

Protocol amendments:

The following amendments were made before the inclusion of the fifth patient. Regarding the Inclusion criteria, the age limit of 65 was excluded. The visual analogue scale was changed to NRS because NRS is used in care as usual. Before the first healthy volunteer's inclusion, the number of visits was reduced from three to one. The latest protocol, version 5, is from 06-07-2020. Due to the COVID-19 pandemic, the study was put on hold, and an amendment (version 5) was submitted and approved on August 11, 2020, to prolong the initial inclusion date for the participants to March 1, 2022.

Declaration of interest:

Not applicable

IPD sharing statement

The de-identified individual clinical trial participant-level data (IPD) will be shared. All individual participant data that underlie the results reported in this article will be shared after de-identification (text, tables, figures, and appendices). The study protocol will also be available. The data will be available beginning nine months following article publication and for a maximum period of 15 years. To make the data findable and accessible for others, we will include a description of the UMCG data catalogue data: https://www.groningendatacatalogus.nl/. Researchers who provide a methodologically sound proposal can access the data with a signed data access agreement.

For peer review only - http://bmjopen.bmj.com/site/about/guidelines.xhtml

TRIAL STATUS

This trial is ongoing. The planned end date for the recruitment of patients and healthy volunteers is March 1, 2022

Authors Contributions

HT, GG, KP, LAN and AW designed the trial protocol. GG and AW secured funding for the study. IS, HT and AW drafted the manuscript. GG, LAN and KP contributed to the manuscript. All authors read and approved the final manuscript. IS, HT and AW are involved in data collection during the study. All authors are involved in the data analysis and manuscript preparations after the study is completed.

Competing interests statement

The authors declare that they have no competing interests.

Funding

The University of Groningen provided funding, University Medical Center Groningen, the Graduate Sçhool of Medicine and Department of Anesthesiology, Pain Center, Groningen, the Netherlands and the Ministry of Education of Brazil - Science without Borders programme (PhD scholarship to Ms Tharcila Chaves – project number 9140/13-1), Rio de Janeiro, Brazil. The funding bodies had no role in the study design and collection, analysis and interpretation of data, and writing of the manuscript.

For peer review only

For peer review only - http://bmjopen.bmj.com/site/about/guidelines.xhtml

REFERENCES

1. Spijker-Huiges A, Groenhof F, Winters JC, et al. Radiating low back pain in general practice: incidence, prevalence, diagnosis, and long-term clinical course of illness. *Scand J Prim Health Care* 2015;33(1):27-32. doi: 10.3109/02813432.2015.1006462 [published Online First: 2015/02/20]
2. Bogduk N, McGuirk B. Medical management of acute and chronic low back pain: an evidence-based approach. Amsterdam2002.
3. Wolff AP, Groen GJ, Wilder-Smith OH. Diagnosis of chronic radiating lower back pain without overt focal neurologic deficits: what is the value of segmental nerve blocks? *Therapy* 2005;2(4):577-85.
4. Wolff AP, Groen GJ, Crul BJ. Diagnostic lumbosacral segmental nerve blocks with local anesthetics: a prospective double-blind study on the variability and interpretation of segmental effects. *Reg Anesth Pain Med* 2001;26(2):147-55. doi: 10.1053/rapm.2001.21436 [published Online First: 2001/03/17]
5. Stolker RJ, Vervest ACM, Groen GJ. The management of chronic spinal pain by blockades: a review. *Pain* 1994;58(1):1-20.
6. Tajima T, Furamochi K, Kuramochi E. Selective Lumbosacral Radiculography and Block. *Spine* 1980;5(1):68-77.
7. Krempen JF, Smith BS. Nerve-root injection: a method for evaluating the etiology of sciatica. *JBJS* 1974;56(7):1435-44.
8. Herron LD. Selective nerve root block in patient selection for lumbar surgery: surgical results. *Journal of spinal disorders* 1989;2(2):75-79.
9. Dooley J, McBroom R, Taguchi T, et al. Nerve root infiltration in the diagnosis of radicular pain. *Spine* 1988;13(1):79-83.
10. Pelletier R, Higgins J, Bourbonnais D. Is neuroplasticity in the central nervous system the missing link to our understanding of chronic musculoskeletal disorders? *BMC Musculoskelet Disord* 2015;16:25. doi: 10.1186/s12891-015-0480-y [published Online First: 2015/04/19]
11. Arendt-Nielsen L, Morlion B, Perrot S, et al. Assessment and manifestation of central sensitisation across different chronic pain conditions. *Eur J Pain* 2018;22(2):216-41. doi: 10.1002/ejp.1140 [published Online First: 2017/11/07]
12. Müller M, Curatolo M, Limacher A, et al. Predicting transition from acute to chronic low back pain with quantitative sensory tests—a prospective cohort study in the primary care setting. *European journal of pain* 2019;23(5):894-907.
13. van Helmond N, Aarts HM, Timmerman H, et al. Is preoperative quantitative sensory testing related to persistent postsurgical pain? A systematic literature review. *Anesthesia & Analgesia* 2020;131(4):1146-55.
14. Petersen KK, Vaegter HB, Stubhaug A, et al. The predictive value of quantitative sensory testing: a systematic review on chronic postoperative pain and the analgesic effect of pharmacological therapies in patients with chronic pain. *Pain* 2021;162(1):31-44.
15. Bridges D, Thompson SW, Rice AS. Mechanisms of neuropathic pain. *Br J Anaesth* 2001;87(1):12-26. doi: 10.1093/bja/87.1.12 [published Online First: 2001/07/20]
16. Finco G, Evangelista M, Sardo S. Basic guide to chronic pain assessment: from neurophysiology to bedside. *Minerva Anestesiologica* 2020;86(12):1321-30.
17. Woolf CJ. Central sensitization: implications for the diagnosis and treatment of pain. *Pain* 2011;152(3 Suppl):S2-15. doi: 10.1016/j.pain.2010.09.030 [published Online First: 2010/10/22]
18. von Hehn CA, Baron R, Woolf CJ. Deconstructing the neuropathic pain phenotype to reveal neural mechanisms. *Neuron* 2012;73(4):638-52. doi: 10.1016/j.neuron.2012.02.008 [published Online First: 2012/03/01]

For peer review only - http://bmjopen.bmj.com/site/about/guidelines.xhtml

19. Konopka KH, Harbers M, Houghton A, et al. Bilateral sensory abnormalities in patients with unilateral neuropathic pain; a quantitative sensory testing (QST) study. *PLoS One* 2012;7(5):e37524. doi: 10.1371/journal.pone.0037524 [published Online First: 2012/05/26]
20. Rolke R, Magerl W, Campbell KA, et al. Quantitative sensory testing: a comprehensive protocol for clinical trials. *Eur J Pain* 2006;10(1):77-88. doi: 10.1016/j.ejpain.2005.02.003 [published Online First: 2005/11/18]
21. Mehta V, Snidvongs S, Ghai B, et al. Characterization of peripheral and central sensitization after dorsal root ganglion intervention in patients with unilateral lumbosacral radicular pain: a prospective pilot study. *Br J Anaesth* 2017;118(6):924-31. doi: 10.1093/bja/aex089 [published Online First: 2017/06/03]
22. Kregel J, Vuijk PJ, Descheemaeker F, et al. The Dutch Central Sensitization Inventory (CSI): Factor Analysis, Discriminative Power, and Test-Retest Reliability. *Clin J Pain* 2016;32(7):624-30. doi: 10.1097/AJP.0000000000000306 [published Online First: 2015/09/30]
23. Platform WHOICTR. WHO Trial Registration Data Set (Version 1.3) [Available from: http://awareness.who.int/ictrp/network/trds_v1.3/en/ accessed 5 January 2021.
24. Chan A-W, Tetzlaff JM, Altman DG, et al. SPIRIT 2013 statement: defining standard protocol items for clinical trials. *Annals of internal medicine* 2013;158(3):200-07.
25. Mayer TG, Neblett R, Cohen H, et al. The development and psychometric validation of the central sensitization inventory. *Pain Practice* 2012;12(4):276-85.
26. Sullivan MJ, Bishop SR, Pivik J. The pain catastrophizing scale: development and validation. *Psychological assessment* 1995;7(4):524.
27. Osman A, Barrios FX, Kopper BA, et al. Factor structure, reliability, and validity of the Pain Catastrophizing Scale. *Journal of behavioral medicine* 1997;20(6):589-605.
28. Crombez G, Eccleston C, Baeyens F, et al. When somatic information threatens, catastrophic thinking enhances attentional interference. *Pain* 1998;75(2-3):187-98.
29. Crombez G, Vlaeyen JW, Heuts PH, et al. Pain-related fear is more disabling than pain itself: evidence on the role of pain-related fear in chronic back pain disability. *Pain* 1999;80(1-2):329-39.
30. Van Damme S, Crombez G, Bijttebier P, et al. A confirmatory factor analysis of the Pain Catastrophizing Scale: invariant factor structure across clinical and non-clinical populations. *Pain* 2002;96(3):319-24.
31. McCracken LM. “Attention” to pain in persons with chronic pain: a behavioral approach. *Behavior therapy* 1997;28(2):271-84.
32. Roelofs J, Peters ML, McCracken L, et al. The pain vigilance and awareness questionnaire (PVAQ): further psychometric evaluation in fibromyalgia and other chronic pain syndromes. *Pain* 2003;101(3):299-306.
33. Van der Zee K, Sanderman R. RAND-36. *Groningen: Northern Centre for Health Care Research, University of Groningen, the Netherlands* 1993;28(6)
34. Hays RD, Sherbourne CD, Mazel RM. The rand 36-item health survey 1.0. *Health economics* 1993;2(3):217-27.
35. Pollard CA. Preliminary validity study of the pain disability index. *Perceptual and motor skills* 1984
36. Soer R, Köke AJ, Vroomen PC, et al. Extensive validation of the pain disability index in 3 groups of patients with musculoskeletal pain. *Spine* 2013;38(9):E562-E68.
37. Tait RC, Pollard CA, Margolis RB, et al. The Pain Disability Index: psychometric and validity data. *Arch Phys Med Rehabil* 1987;68(7):438-41.
38. Grönblad M, Hupli M, Wennerstrand P, et al. Intercorrelation and test-retest reliability of the Pain Disability Index (PDI) and the Oswestry Disability Questionnaire (ODQ) and their correlation with pain intensity in low back pain patients. *The Clinical journal of pain* 1993;9(3):189-95.
39. Ilmarinen J. The work ability index (WAI). *Occupational medicine* 2007;57(2):160-60.
40. Tuomi K, Ilmarinen J, Jahkola A, et al. Work ability index: Finnish Institute of Occupational Health Helsinki 1998.

For peer review only - http://bmjopen.bmj.com/site/about/guidelines.xhtml

41. Psychometric properties of Work Ability Index in the light of comparative survey study. International congress series; 2005. Elsevier.
42. De Zwart B, Frings-Dresen M, Van Duivenbooden J. Test–retest reliability of the Work Ability Index questionnaire. *Occupational medicine* 2002;52(4):177-81.
43. Hill JC, Whitehurst DG, Lewis M, et al. Comparison of stratified primary care management for low back pain with current best practice (STarT Back): a randomised controlled trial. *The Lancet* 2011;378(9802):1560-71.
44. Bier JD, Ostelo RW, van Hooff ML, et al. Validity and reproducibility of the STarT Back Tool (Dutch Version) in Patients With Low Back Pain in Primary Care Settings. *Physical Therapy* 2017;97(5):561-70.
45. Storm L, Rousing R, Andersen M, et al. Usefulness of the STarT Back Screening Tool to predict pain problems after lumbar spine surgery. *Dan Med J* 2018;65(12):1-4.
46. Backonja MM, Attal N, Baron R, et al. Value of quantitative sensory testing in neurological and pain disorders: NeuPSIG consensus. *Pain* 2013;154(9):1807-19. doi: 10.1016/j.pain.2013.05.047 [published Online First: 2013/06/08]
47. Weinstein S. Intensive and extensive aspects of tactile sensitivity as a function of body part, sex and laterality. *The skin senses* 1968
48. Fruhstorfer H, Gross W, Selbmann O. von Frey hairs: new materials for a new design. *European Journal of Pain* 2001;5(3):341-42.
49. Baumgärtner U, Magerl W, Klein T, et al. Neurogenic hyperalgesia versus painful hypoalgesia: two distinct mechanisms of neuropathic pain. *Pain* 2002;96(1-2):141-51.
50. LaMotte RH, Shain CN, Simone DA, et al. Neurogenic hyperalgesia: psychophysical studies of underlying mechanisms. *Journal of neurophysiology* 1991;66(1):190-211.
51. Ziegler E, Magerl W, Meyer R, et al. Secondary hyperalgesia to punctate mechanical stimuli: central sensitization to A-fibre nociceptor input. *Brain* 1999;122(12):2245-57.
52. Samuelsson M, Leffler A-S, Hansson P. Dynamic mechanical allodynia: on the relationship between temporo-spatial stimulus parameters and evoked pain in patients with peripheral neuropathy. *Pain* 2005;115(3):264-72.
53. Chan A, MacFarlane I, Bowsher D, et al. Weighted needle pinprick sensory thresholds: a simple test of sensory function in diabetic peripheral neuropathy. *Journal of Neurology, Neurosurgery & Psychiatry* 1992;55(1):56-59.
54. Greenspan JD, McGillis SL. Thresholds for the perception of pressure, sharpness, and mechanically evoked cutaneous pain: effects of laterality and repeated testing. *Somatosensory & motor research* 1994;11(4):311-17.
55. Hampf G, Bowsher D, Wells C, et al. Sensory and autonomic measurements in idiopathic trigeminal neuralgia before and after radiofrequency thermocoagulation: differentiation from some other causes of facial pain. *Pain* 1990;40(3):241-48.
56. Graven-Nielsen T, Wodehouse T, Langford RM, et al. Normalization of widespread hyperesthesia and facilitated spatial summation of deep-tissue pain in knee osteoarthritis patients after knee replacement. *Arthritis Rheum* 2012;64(9):2907-16. doi: 10.1002/art.34466 [published Online First: 2012/03/17]
57. Starkweather AR, Heineman A, Storey S, et al. Methods to measure peripheral and central sensitization using quantitative sensory testing: A focus on individuals with low back pain. *Appl Nurs Res* 2016;29:237-41. doi: 10.1016/j.apnr.2015.03.013 [published Online First: 2016/02/10]
58. Neblett R, Cohen H, Choi Y, et al. The Central Sensitization Inventory (CSI): establishing clinically significant values for identifying central sensitivity syndromes in an outpatient chronic pain sample. *The Journal of Pain* 2013;14(5):438-45.

For peer review only - http://bmjopen.bmj.com/site/about/guidelines.xhtml

TABLES

Table 1: Criteria for the inclusion and exclusion of patients

Inclusion criteria	Exclusion criteria
• Male and female patients<br>• Age 18 years or older<br>• Presence of chronic low back pain radiating in the leg<br>• Leg pain more or equal to back pain<br>• A physician must consider therapeutic sensory nerve root blocking or pulsed radiofrequency as an appropriate treatment intervention<br>• Agreement and signature of the informed consent	• Exclusion criteria for selective nerve blocks, according to local protocol<br>• Not or not sufficient understanding of the Dutch language<br>• Incapacity to follow instructions<br>• Mental incompetence to provide informed consent<br>• Chronic low back pain with radiation to both legs<br>• Pain in one (or more) sites where quantitative sensory testing will be applied except for the most painful point in the painful dermatome

For peer review only

For peer review only - http://bmjopen.bmj.com/site/about/guidelines.xhtml

Table 2: Criteria for the inclusion and exclusion of healthy **volunteers**

Inclusion criteria	Exclusion criteria
• Male and female healthy volunteers<br>• Age 18 years or older<br>• No history of low back pain<br>• Agreement and signature of the consent	• Not matching with one of the included patients based on sex and age (plus or minus three years of age)<br>• Not or not sufficient understanding of the Dutch language<br>• Incapacity to follow instructions<br>• Mental incompetence to provide informed consent

For peer review only

Table 3: Overview of the variables collected and interventions per visit

	Patients					Healthy volunteers
Demographics	Age, gender, weight, height, co-morbidities, pain medication, ethnic background and education.					
		V1a	V1b, V1c	V2	V3	HV
Time between visits		-	One week	One week	Four weeks	-
Questionnaires	CSI	✓	✓	✓	✓	✓
	SBST	✓	-	-	✓	✓
	RAND-36	✓	-	-	✓	✓
	PDI	✓	-	-	✓	✓
	WAI	✓	-	-	✓	✓
	PVAQ	✓	-	-	✓	✓
	PCS	✓	-	-	✓	✓
	Pain Drawing	✓	-	-	✓	-
	NRS pain	✓	✓	✓	✓	✓
QST	MDT	✓	✓	✓	✓	✓
	DMA	✓	✓	✓	✓	✓
	MPT	✓	✓	✓	✓	✓
	WUR	✓	✓	✓	✓	✓
	PPT	✓	✓	✓	✓	✓
	Cuff PPT	✓	✓	✓	✓	✓
	Cuff TS	✓	✓	✓	✓	✓
	CPM	✓	✓	✓	✓	✓
Intervention	dSNRB	✓	✓	-	-	-
	tSNRB or pRF	-	-	✓	-	-

Legend: V: visit, HV: healthy volunteers, QST: quantitative sensory testing; MDT: mechanical detection threshold; DMA: dynamic mechanical allodynia; MPT: mechanical pain threshold; WUR: Wind-up ratio; PPT: pressure pain threshold; Cuff PPT: pressure pain threshold by cuff algometer; Cuff TS: temporal summation by cuff algometer; CPM: conditioned pain

For peer review only - http://bmjopen.bmj.com/site/about/guidelines.xhtml

modulation; CSI: Central Sensitisation Inventory; SBST: STarT Back Screening Tool; RAND-36: RAND 36-Item Health Survey; PDI: Pain Disability Index; WAI: Work Ability Index; PVAQ: Pain Vigilance and Awareness Questionnaire; PCS: Pain Catastrophising Scale; Pain Drawing: Drawing in standard leg images. NRS-pain: Numeric Rating Scale for Pain; dSNRB: diagnostic selective nerve root block, tSNRB: therapeutic selective nerve root block, pRF: pulsed radiofrequency

For peer review only

For peer review only - http://bmjopen.bmj.com/site/about/guidelines.xhtml

Table 4: Sequence of QST measurement sites

Visit	Sequence
Visit 1a	Site D-A-C-B
Visit 1b	Site D-C-A-B
Visit 1c	Site D-B-A-C
Visit 2	Site D-C-B-A
Visit 3	Site D-B-C-A

For peer review only

For peer review only - http://bmjopen.bmj.com/site/about/guidelines.xhtml

Table 5: QST measurement sites:

Site A	The most painful point in the painful dermatome
Site B	Contralateral point of location A
Site C	Control site 1 (contralateral of location A, distant from the painful area), between the scapulae. On the medial part of trapezius muscle at the height of the spina scapulae, 4cm lateral to the spinous process of the 3rd thoracic vertebra (Th3)
Site D	Control site 2 (distant from the painful area): contralateral to location A, at the deltoid muscle: On the medial part of the deltoid muscle, 3cm below the acromion
Site E	On the rectus femoris muscle 15 cm proximal to the base of the patella
Site F	On the lower leg. At the level of the largest circumference of gastrocnemius muscle.

For peer review only

For peer review only - http://bmjopen.bmj.com/site/about/guidelines.xhtml

Figure 1: Flowchart of the study procedure

Legend: QST: Quantitative sensory testing; dSNRB: diagnostic selective nerve root block; tSNRB: therapeutic selective nerve root block; pRF: Pulsed radiofrequency.

For peer review only

For peer review only - http://bmjopen.bmj.com/site/about/guidelines.xhtml

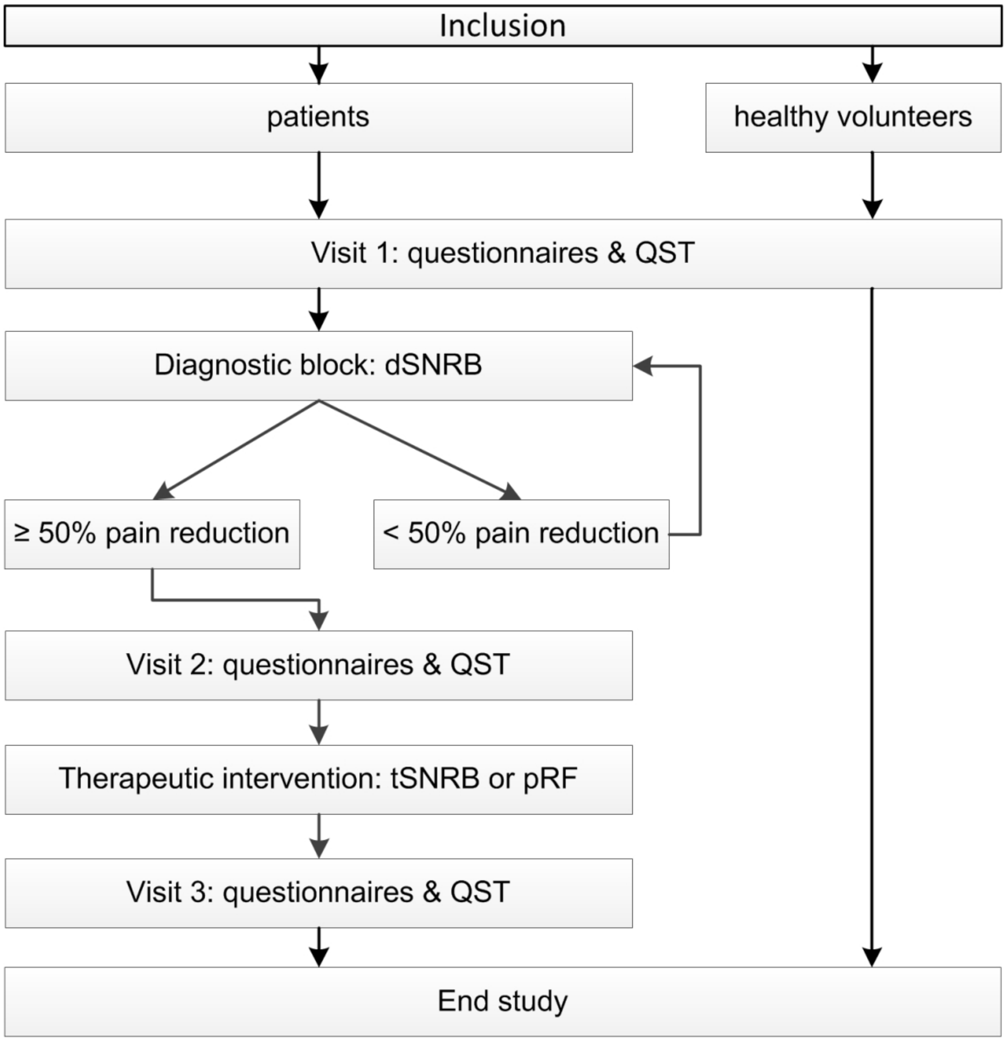

Figure 1: Flowchart of the study procedure / QST: Quantitative sensory testing; dSNRB: diagnostic selective nerve root block; tSNRB: therapeutic selective nerve root block; pRF: Pulsed radiofrequency.

119x124mm (300 x 300 DPI)

APPENDICES

Appendix A: Informed consent materials:

A model consent form and other related documentation is, in Dutch, given to participants and authorised surrogates

Bijlage B:

Toestemmingsformulier proefpersoon

Onderzoek naar de gevoeligheid van het centrale zenuwstelsel bij proefpersonen met chronische lage rugpijn uitstralend naar het been.

(Centrale sensitisatie in proefpersonen met chronische lage rugpijn uitstralend naar het been)

- Ik heb de informatiebrief gelezen. Ook kon ik vragen stellen. Mijn vragen zijn voldoende beantwoord. Ik had genoeg tijd om te beslissen of ik meedoe.
- Ik weet dat meedoen vrijwillig is. Ook weet ik dat ik op ieder moment kan beslissen om toch niet mee te doen of te stoppen met het onderzoek. Daarvoor hoef ik geen reden te geven.
- Ik weet dat sommige mensen mijn persoonsgegevens en/of gecodeerde onderzoeksgegevens kunnen inzien. Die mensen staan vermeld in deze informatiebrief.
- Ik geef toestemming om mijn gegevens nog 15 jaar na dit onderzoek te bewaren.
- Ik wil meedoen aan dit onderzoek.

Naam proefpersoon:

Handtekening: Datum : __ / __ / __

Ik verklaar dat ik deze proefpersoon volledig heb geïnformeerd over het genoemde onderzoek.

Als er tijdens het onderzoek informatie bekend wordt die de toestemming van de proefpersoon zou kunnen beïnvloeden, dan breng ik hem/haar daarvan tijdig op de hoogte.

Naam onderzoeker (of diens vertegenwoordiger):

Handtekening: Datum: __ / __ / __

<indien van toepassing>

Aanvullende informatie is gegeven door:

Naam:

Functie:

Handtekening: Datum: __ / __ / __

----

* Doorhalen wat niet van toepassing is.

De proefpersoon krijgt een volledige informatiebrief mee, samen met een kopie van het getekende toestemmingsformulier.

For peer review only - http://bmjopen.bmj.com/site/about/guidelines.xhtml

Appendix B: Intervention procedures

dSNRB

Diagnostic selective nerve root blocks (dSNRB) are performed in a fluoroscopy suite with monitoring equipment. At a minimum, pulse and blood pressure should be monitored every 1 to 5 minutes, depending on the procedure. Assisting personnel include nurses trained in advanced cardiac life support and a radiology technician. All procedures are performed with fluoroscopic guidance. Patient monitoring does not end after injecting medication. Post-procedure assessment is necessary because side effects or complications such as allergic reaction, vasovagal response, cardiac or respiratory depression, or high spinal block can occur. To ascertain whether the diagnostic selective nerve root block response was positive or negative, patients should perform activities or assume positions that invariably incite their pain. If a change in preprocedure and postprocedure verbal numeric rating scale rating of a minimum of 50% pain relief occurs, then the dSNRB result is considered positive.
For a lumbar dSNRB, the patient is placed in a prone position on the fluoroscopy table. The back is prepared and draped in a sterile manner. The level is located by establishing the lowest lumbar segment. The C-arm is rotated in the oblique position to allow access to the intervertebral foramen. Care must be maintained not to start too laterally to avoid renal or peritoneal puncture. A skin wheal is raised with 1% xylocaine. A 22-gauge, 6 or 10 cm with 5mm nonisolated tip needle is directed toward the foramen in the oblique position. Once the needle has penetrated the muscle adjacent to the spine, the needle then is advanced slowly in the AP plane to the 6-o'clock position of the pedicle and just inferior to the pedicle. The needle's position is in the safe triangle formed by the pedicle, the foramen's outer line, and the spinal nerve. Care is taken not to pierce the spinal nerve. Needle position is checked in the AP and lateral planes. One millilitre of non-ionic contrast agent is injected to outline the spinal nerve and dorsal root ganglion. The contrast pattern is evaluated to ensure that there is no vascular pattern, subarachnoid flow, or epidural spread to adjacent levels. One millilitre of 0.75% 0.3ml with Visipaque 320 mg I/ml 0.3ml (total 0.6ml) is infused.

Reference

Gauci, C. A. (2011). Manual of RF Techniques-a Practical Manual of Radiofrequency Procedures in Chronic Pain Management(3th Edition), CoMedical, 2011. Ridderkerk, the Netherlands.

Huston, C. W., & Slipman, C. W. (2002). Diagnostic selective nerve root blocks: indications and usefulness. *Physical medicine and rehabilitation clinics of North America*, 13(3), 545-565.

For peer review only - http://bmjopen.bmj.com/site/about/guidelines.xhtml

Perdok, J.M., Wolff, A.P., Stellema, R., Groen, G.J. (2017) 'Wortelblokkades'. Local protocol University Medical Center Groningen.

tSNRB

Therapeutic selective nerve root block (tSNRB) is similar to the dSNRB. The tSNRB is performed under sterile conditioned with an image intensifier. The target point was "safe triangle," i.e., above the exiting nerve root and below the corresponding pedicle. The skin is anaesthetised with 2-3 ml mepivacaine 2%. A spinal needle (22G) was inserted paramedian through the skin and muscles in a cranio-medial direction until a bony contact was encountered. This method allows advancing the needle in a safe triangle without contact with the nerve root. After verifying a correct needle positioning under biplanar image intensifier control, non-ionic contrast was injected until a radiculogram is obtained. Subsequently, 1ml 0.75% Bupivacaine and 1ml (40mg) Triamcinolon and 320mg I/ml Visipaque is injected. It should be stressed that this was a periradicular and not an intraneural injection. This technique is for the nerve roots L3-L5; for S1, a different technique is required. First, the image intensifier is positioned perpendicular to the foramen S1. A spinal needle is inserted perpendicular to the surface of the sacrum into the foramen. An image intensifier checks the correct needle positioning in two planes. After obtaining a correct periradiculargram, 1ml 0.75% Bupivacaine and 1ml (40mg) Triamcinolon and 320mg I/ml Visipaque is injected. The patients have clinical surveillance on the ward for about 30 minutes to account for unexpected side effects.

Reference

Bogduk N, Aprill C, Derby R, Selective nerve root blocks. In: Wilson DJ, ed. Interventional Radiology of the Muskuloskeletal System. London: Edward Arnold; 1995. p. 122–32.

Gauci, C. A. (2011). Manual of RF Techniques-a Practical Manual of Radiofrequency Procedures in Chronic Pain Management(3th Edition), CoMedical, 2011. Ridderkerk, the Netherlands.

Perdok, J.M., Wolff, A.P., Stellema, R., Groen, G.J. (2017) 'Wortelblokkades'. Local protocol University Medical Center Groningen.

Narozny, M., Zanetti, M., & Boos, N. (2001). Therapeutic efficacy of selective nerve root blocks in the treatment of lumbar radicular leg pain. *Swiss medical weekly*, *131*(0506).

pRF

For peer review only - http://bmjopen.bmj.com/site/about/guidelines.xhtml

Pulsed radiofrequency (pRF) is in a procedure similar to the tSNRB. The pRF is performed under sterile conditioned with an image intensifier. The target point was "safe triangle," i.e., above the exiting nerve root and below the corresponding pedicle. The skin is anaesthetised with 2-3 ml mepivacaine 2%. A spinal needle (22G) was inserted paramedian through the skin and muscles in a cranio-medial direction until a bony contact was encountered. This method allows advancing the needle in a safe triangle without contact with the nerve root. After verifying a correct needle positioning under biplanar image intensifier control, non-ionic contrast was injected until a radiculogram is obtained.

The tissue temperature is held at or below 42˚C on average. Radiofrequency is delivered in short, high-intensity bursts so that the radiofrequency electric field strength is increased without gross heating. The clinical objective is a neural modification by electric and thermal fields. This technique is for the nerve roots L3-L5; for S1, a different technique is required. First, the image intensifier is positioned perpendicular to the foramen S1. A spinal needle is inserted perpendicular to the surface of the sacrum into the foramen. An image intensifier checks the correct needle positioning in two planes. After obtaining a correct periradiculargram, the tissue temperature is held at or below 42˚C on average. Radiofrequency is delivered in short, high-intensity bursts so that the radiofrequency electric field strength is increased without gross heating. The patients have clinical surveillance on the ward for about 30 minutes to account for any unexpected side effects

Reference

Ewertowska, E., Mercadal, B., Muñoz, V., Ivorra, A., Trujillo, M., & Berjano, E. (2018). Effect of applied voltage, duration and repetition frequency of RF pulses for pain relief on temperature spikes and electrical field: a computer modelling study. *International Journal of Hyperthermia*, *34*(1), 112-121.

Gauci, C. A. (2011). Manual of RF Techniques-a Practical Manual of Radiofrequency Procedures in Chronic Pain Management(3th Edition), CoMedical, 2011. Ridderkerk, the Netherlands.

Perdok, J.M., Wolff, A.P., Stellema, R., Groen, G.J. (2017) ‘Wortelblokkades’. Local protocol University Medical Center Groningen.

For peer review only - http://bmjopen.bmj.com/site/about/guidelines.xhtml

Appendix C: Images drawing the most painful spot on the leg.

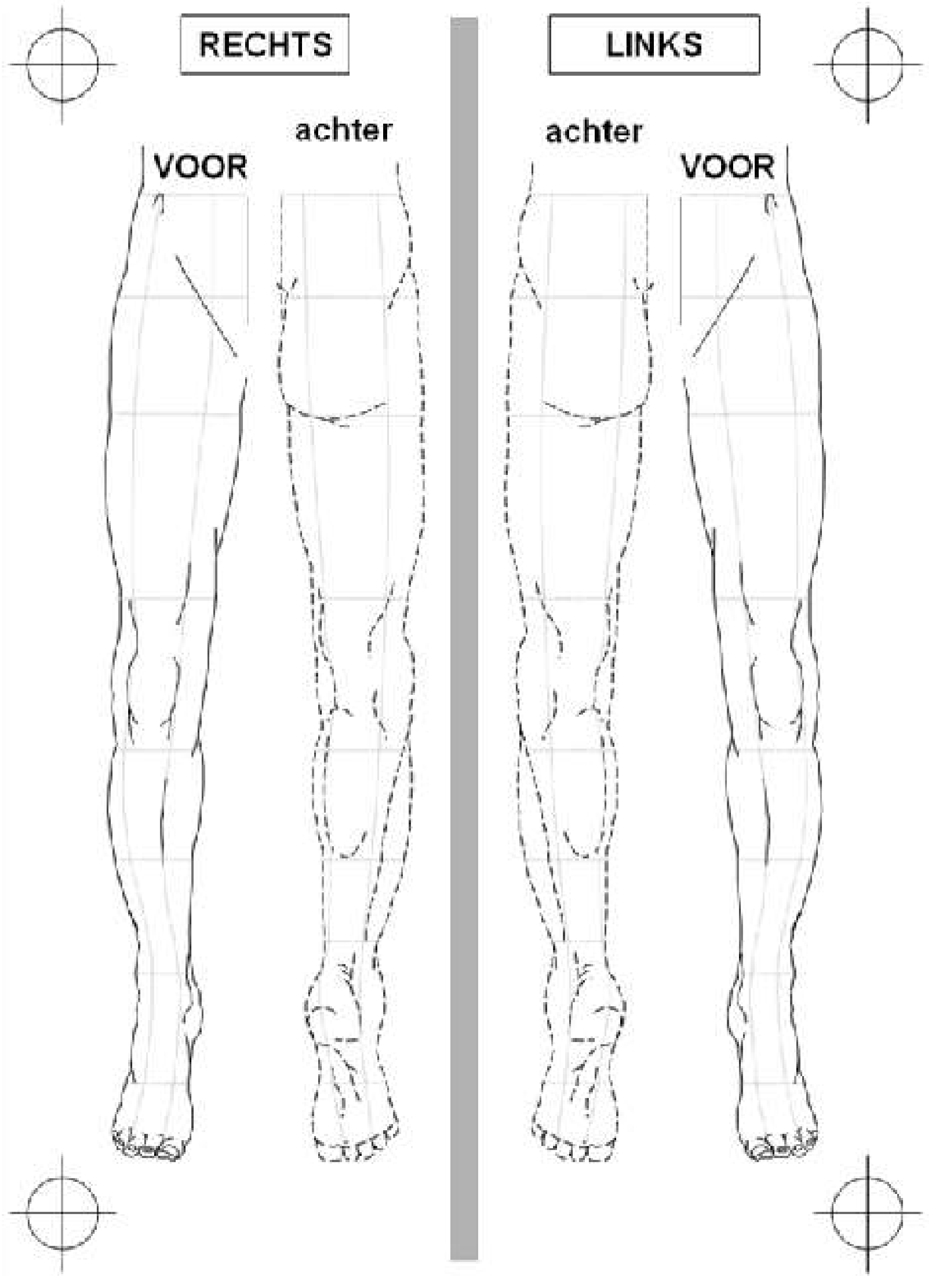

BMJ Open

Human Assumed Central Sensitisation (HACS) in Patients with Chronic Low Back Pain Radiating to the Leg (CLaSSICO study):Protocol for a Prospective Observational Study.

Journal:	*BMJ Open*
Manuscript ID	bmjopen-2021-052703.R2
Article Type:	Protocol
Date Submitted by the Author:	29-Nov-2021
Complete List of Authors:	Schuttert, Ingrid; UMCG, Department of Anesthesiology, Pain Center<br>Timmerman, Hans; University Medical Centre Groningen, Department of Anesthesiology, Pain Center<br>Groen, Gerbrand; UMCG, Department of Anesthesiology, Pain Center<br>Petersen, Kristian; Aalborg Universitet, Department of Health Science and Technology<br>Arendt-Nielsen, Lars; Aalborg Universitet, Department of Health Science and Technology<br>Wolff, Andre; Universitair Medisch Centrum Groningen, Department of Anesthesiology, Pain Center
**Primary Subject Heading**:	Anaesthesia
Secondary Subject Heading:	Research methods
Keywords:	Pain management < ANAESTHETICS, Back pain < ORTHOPAEDIC & TRAUMA SURGERY, Neurological pain < NEUROLOGY, PAIN MANAGEMENT

SCHOLARONE™
Manuscripts

Human Assumed Central Sensitisation (HACS) in Patients with Chronic Low Back Pain Radiating to the Leg (CLaSSICO study): Protocol for a Prospective Observational Study.

AUTHORS

Ingrid Schuttert*, MSc, Department of Anesthesiology, Pain Center, University of Groningen, University Medical Center Groningen (UMCG), Groningen, the Netherlands.
OrcID: 0000-0003-2164-4721

Hans Timmerman, PhD, Department of Anesthesiology, Pain Center, University of Groningen, University Medical Center Groningen (UMCG), Groningen, the Netherlands.
OrcID: 0000-0002-6082-9043

Gerbrand J. Groen, MD, PhD, Department of Anesthesiology, Pain Center, University of Groningen, University Medical Center Groningen (UMCG), Groningen, the Netherlands.
OrcID: 0000-0002-7199-0107

Kristian K. Petersen, PhD, SMI, Center for Neuroplasticity and Pain, Department of Health Science and Technology, Faculty of Medicine, Aalborg University, Aalborg, Denmark.
ORCID: 0000-0003-4506-000X

Lars Arendt-Nielsen, PhD, SMI, Center for Neuroplasticity and Pain, Department of Health Science and Technology, Faculty of Medicine, Aalborg University, Aalborg, Denmark.
ORCID: https://orcid.org/0000-0003-0892-1579

André P. Wolff, MD, PhD, Department of Anesthesiology, Pain Center, University of Groningen, University Medical Center Groningen (UMCG), Groningen, the Netherlands.
OrcID: 0000-0001-6240-3903

* Corresponding author for public and scientific queries:

Ingrid Schuttert
Department of Anesthesiology, UMCG Pain Center, University Medical Center Groningen (UMCG), PO Box 30002, 9751 ND Haren, the Netherlands.
Email: i.schuttert@umcg.nl
Tel: +31503612140

Word count: 4147(Word count, excluding title page, abstract, references, figures and tables)

For peer review only

For peer review only - http://bmjopen.bmj.com/site/about/guidelines.xhtml

DECLARATIONS

Ethics approval and consent to participate

The medical and ethical review board Committee on Research Involving Human Subjects of the University Medical Center Groningen, Groningen, the Netherlands, has approved the study "Central sensitisation in patients with chronic low back pain radiating to the leg", dossier number NL60439.042.17 on November 1, 2017. Due to COVID-19, an amendment was submitted and approved on August 11, 2020, to prolong the participants' inclusion date due to March 1, 2022. The participants will receive written and oral information about the study and all assessments. The participants will receive written and oral information about the study and assessments. Before the start of the study, written informed consent will be provided by all participants (see appendix A).

Consent for publication

Not applicable.

Availability of data and materials

Due to the data's sensitivity and the restrictions from informed consent, the data will not be stored in a public repository. The data and meta-data will be stored at a repository at the UMCG, ensuring the data's security and backup. UMCG pursues a FAIR data policy for research conducted in the UMCG. To make the data findable and accessible for others, we will include a description of the UMCG data catalogue data: https://www.groningendatacatalogus.nl/. The data's metadata will be made available for researchers inside and outside the institute via the catalogue. This catalogue is in sync with relevant (inter)national catalogues, such as Biobanking and Biomolecular Resources Research Infrastructure and National Academic Research and Collaborations Information System. A data access committee has been put in place to review requests and assure the accessibility of the data. This access committee can be reached via the corresponding author (Ingrid Schuttert, e-mail: i.schuttert@umcg.nl or André Wolff, e-mail: a.p.wolff@umcg.nl). The results will be published in peer-reviewed scientific journals and submitted for presentation at relevant national and international scientific meetings/conferences.

Acknowledgements

Not applicable.

Patient consent for publication

For peer review only

For peer review only - http://bmjopen.bmj.com/site/about/guidelines.xhtml

Not required.

Keywords

Chronic Low Back Pain; Sensitisation; Study protocol; Quantitative sensory testing (QST); Selective nerve root block (SNRB); Pulsed radiofrequency (PRF), Human Assumed Central Sensitisation (HACS).

For peer review only

For peer review only - http://bmjopen.bmj.com/site/about/guidelines.xhtml

ABBREVIATIONS

ANOVA:	ANalysis Of VAriance
CLBPr:	Chronic Low Back Pain radiating to the leg;
CSI:	Central Sensitisation Inventory;
dSNRB:	Diagnostic Selective Nerve Root Block
Dlv	Dutch language version
HACS	Human Assumed Central Sensitisation
IQR:	Interquartile range
NRS:	Numerical Rating Scale
pRF:	Pulsed Radiofrequency;
QST:	Quantitative Sensory Testing;
RAND-36:	Rand 36-Item Health Survey
SBST:	STarT Back Screening Tool
tSNRB:	Therapeutic Selective Nerve Root Block

For peer review only

For peer review only - http://bmjopen.bmj.com/site/about/guidelines.xhtml

ABSTRACT

Introduction: Patients with chronic low back pain radiating to the leg (CLBPr) are sometimes referred to a specialised pain clinic for a precise diagnosis based, for example, on a diagnostic selective nerve root block. Possible interventions are therapeutic selective nerve root block or pulsed radiofrequency. Central pain sensitisation is not directly assessable in humans and therefore the term 'human assumed central sensitisation' (HACS) is proposed. The possible existence and degree of sensitisation associated with pain mechanisms assumed present in the human central nervous system, its role in the chronification of pain and its interaction with diagnostic and therapeutic interventions are largely unknown in patients with CLBPr. The aim of quantitative sensory testing (QST) is to estimate quantitatively the presence of HACS and accumulating evidence suggest that a subset of patients with CLBPr have facilitated responses to a range of QST tests.
The aims of this study are to identify HACS in patients with CLBPr, to determine associations with the effect of selective nerve root blocks and compare outcomes of HACS in patients to healthy volunteers.

Methods and analysis: A prospective observational study including fifty patients with CLBPr. Measurements are performed before diagnostic and therapeutic nerve root block interventions and at four weeks follow-up. Data from patients will be compared with those of fifty sex- and age-matched healthy volunteers. The primary study parameters are the outcomes of QST and the central sensitisation inventory. Statistical analyses to be performed will be analysis of variance.

Ethics and dissemination: The Medical Research Ethics Committee of the University Medical Center Groningen, Groningen, the Netherlands, approved this study (dossier NL60439.042.17). The results will be disseminated via publications in peer-reviewed journals and at conferences.

Trial registration number: NTR NL6765

For peer review only

For peer review only - http://bmjopen.bmj.com/site/about/guidelines.xhtml

Article Summary

STRENGTHS AND LIMITATIONS OF THIS STUDY

- Strength: comparison with sex- and age-matched healthy controls.
- Strength: Multimodal assessment of human assumed central and peripheral sensitization via objective and subjective measures.
- Limitation: The order of the quantitative sensory testing measures is not randomised for each patient and each visit and is only randomised per visit.

For peer review only

For peer review only - http://bmjopen.bmj.com/site/about/guidelines.xhtml

INTRODUCTION

Background:

The mean incidence and prevalence of chronic low back pain radiating to the leg (CLBPr) are 9.4 and 17.2 per 1000 person-years respectively.[1] If the diagnosis of CLBPr is unclear despite extensive neurological, orthopaedic and radiological examination, patients are sometimes referred to a specialised multidisciplinary pain clinic, where a diagnostic selective nerve root block (dSNRB) might provide a more precise diagnosis.[2 3] This block, achieved by injection of a local anaesthetic, can help determine the predominant segmental level of the pain.[4-9] A positive dSNRB is generally followed by an injection of local anaesthetics and corticosteroids, i.e. a therapeutic SNRB (tSNRB) or a pulsed radiofrequency treatment (pRF) of the spinal nerve root, its dorsal ganglion or a combination thereof.

Growing evidence suggests that central pain mechanisms are facilitated in a subset of patients with CLBPr.[10 11] Facilitation of peripheral and central pain mechanisms have been suggested to be associated with pain progression, poor recovery, poor response to pharmacological interventions and inadequate response to low back surgery, indicating the clinical need of these assessments.[12-14] Preclinical data suggests that nerve fibres can malfunction and respond as nociceptors, resulting in pain from light stimuli, possibly caused by peripheral and central sensitisation.[15] Dysfunction of the somatosensory system may clinically lead to positive sensory symptoms (e.g. allodynia, aftersensations, enhanced temporal summation) and negative sensory symptoms (e.g. hypoesthesia).[11 16-19] Because mechanisms related to central sensitisation can currently only be assessed in animals,[11] proxies are used in humans to assess clinical signs that may be associated with the presence of central pain mechanisms. We use the term 'human assumed central sensitisation' (HACS) because there is no gold standard to estimate the presence of central sensitisation in humans at present. Moreover, there is also no clear definition, method or (clinical) guideline applicable to diagnose central sensitisation in humans.

The role of HACS in the development of CLBPr, in the chronification of pain, and the interaction with diagnostic and therapeutical interventions is not clear.[11] Several methods are thought to assist in the clinical diagnosis of HACS.[11] With Quantitative Sensory Testing (QST) we measure the responses to calibrated graded innocuous or noxious stimuli [20] and evaluate the presence of HACS in patients with CLBPr.[21] Mehta et al. showed that following pRF treatment patients with CLBPr have increased pressure pain thresholds and increased conditioned pain modulation, suggesting a normalization of the sensory pain profile.[21] The presence of HACS might also be evaluated with the Central Sensitisation Inventory (CSI),[22]

For peer review only

For peer review only - http://bmjopen.bmj.com/site/about/guidelines.xhtml

but there are no clinical associations established between the CSI and experimental measures.[11]

Objectives:

The primary objective of this study is to determine if therapeutic selective nerve root block or pulsed radiofrequency in patients with chronic low back pain radiating to one leg changes the outcome of the CSI and the QST measures as proxies for human assumed central sensitisation. Secondary objectives are (1) to determine if human assumed central sensitisation can be assessed using the outcome of the CSI in patients with chronic low back pain radiating to one leg, (2) to determine if a diagnostic selective nerve root block changes the outcome of the CSI and the QST measures as proxies for human assumed central sensitisation in patients with chronic low back pain radiating to one leg, and (3) to determine whether CSI and QST measures as proxies for human assumed central sensitisation (both pre-treatment and post-treatment) among patients differ from those among sex and aged matched healthy volunteers.

Trial design:

A prospective longitudinal observational study about HACS in patients with CLBPr who undergo selective nerve root blocks compared to sex- and age-matched healthy volunteers.

For peer review only - http://bmjopen.bmj.com/site/about/guidelines.xhtml

METHODS

This study was registered prospectively in the Netherlands Trial Register: Trial NL6765 (first posted January 10, 2018). The trial information in this protocol is in line with the WHO Trial Registration Data Set [23] and written following the SPIRIT 2013 Guideline.[24]

Participants, Interventions and outcomes

Study setting:

Patient recruitment, treatment, and measurements take place in the multidisciplinary academic pain center, University Medical Center Groningen (UMCG), Groningen, the Netherlands.

For peer review only

Patient and public involvement:

Patients were not involved in the development of the research question and the determination of the outcome measures. During recruitment, patients are asked to suggest healthy volunteers whose age and gender match their own to participate in the study.

Eligibility criteria:

Fifty consecutive adult patients of both sexes with chronic low back pain radiating in the dermatomes L3 to S2 and fifty sex- and age-matched healthy volunteers will be included. The inclusion and exclusion criteria are summarised in table 1 (Table 1). The inclusion criteria for the healthy volunteers are shown in table 2 (Table 2).

Patients can retain their ongoing pain medication or discontinue it if they stop feeling pain during the study. Other changes in medication use are not allowed during participation. Moreover, all other pain interventions (e.g. physical therapy, transcutaneous electrical nerve stimulation) during their participation in this study are prohibited. Healthy volunteers are matched with the patients on sex and age (plus or minus three years of age). As mentioned, patients are asked to suggest suitable healthy volunteers. If necessary healthy volunteers are also recruited via flyers placed near to the UMCG facilities. Healthy volunteers do not undergo selective nerve root blocks. Patients and healthy volunteers may withdraw from the study at any time for any reason if they wish to do so and without any consequences. The investigator can decide to withdraw a participant from the study for urgent medical reasons.

For peer review only - http://bmjopen.bmj.com/site/about/guidelines.xhtml

Interventions:

The intervention provided in this study is performed in the same way as the standard intervention provided in the multidisciplinary academic pain center and follows the guideline for SNRB (see appendix B for the detailed procedure). Table 3 shows which intervention is used in each visit (Table 3).

Participant timeline:

Patients and healthy volunteers receive a verbal and written explanation of the study procedures and are given sufficient time to consider their participation. After written informed consent is provided, a meeting is scheduled. The flowchart of the study is shown in figure 1 (Figure 1). The schedule for the patient and the healthy volunteer assessments are shown in table 3 (Table 3). Visit 1b, and 1c are only performed for the patients when more than one dSNRB is necessary. After the dSNRB, numerical rating scale (NRS) for pain in the back as well as in the leg are assessed. In case of a pain reduction of ≥ 50%, the patient is considered to have a "positive result", and the therapeutic intervention is planned. The responsible physician will decide which intervention (tSNRB or pRF treatment) each patient will receive (which is part of care as usual). If a pain reduction is < 50%, the patient is considered to have a "negative result". With a negative result, the patient will undergo another dSNRB at a different spinal level (3 dSNRBs are the maximum within this study). dSRBs, tSNRBs and pRF will be performed according to the standard procedures of the multidisciplinary academic pain center. Healthy volunteers are not asked to draw their pain area and do not receive any diagnostic or therapeutic intervention. For healthy volunteers, only baseline assessment is performed.

Recruitment:

At the multidisciplinary academic pain center, on average, two new patients with CLBPr are seen per week. If two patients per month agree to join the study, the sample will be completed within four years. Because patients must visit the multidisciplinary academic pain center once more than during care as usual (visit 3), they will be compensated for this visit when it is not possible to schedule it with another regular hospital visit. Patients will receive compensation for extra travel costs (€0.19/km). Healthy volunteers will receive 10 euros for their visit plus compensation for travel costs (€0.19/km).

Duration

Each visit will last no longer than two and a half hours, consisting of 1 hour measurements (patients and healthy volunteers), 0.5 hour for the treatment and 0.5-1 hour for the

For peer review only

For peer review only - http://bmjopen.bmj.com/site/about/guidelines.xhtml

assessment of the effect of the treatment (patients). Interventions are performed one week apart, and visit 3 will be four weeks after the therapeutic intervention. Depending on the number of dSNRBs performed, the total duration of this study for the patient will take between five to seven weeks (3-5 visits). Healthy volunteers will undergo only one visit with an assessment of 1 hour.

The first patient was included on June 5, 2018. the planned end date for the inclusion of participants is March 1, 2022.

Outcomes:

All measurements are performed for study-specific reasons. The primary outcomes are QST measures and the CSI. Secondary outcomes are Pain Catastrophising Scale, Pain Vigilance and Awareness Questionnaire, 36-Item Short-Form Health Survey (SF-36), Pain Disability Index, Work Ability Index, STarT Back Screening Tool (SBST), Numeric Rating Scale (NRS) and drawing in standard leg images (pain drawing) (Table 3).

Descriptive data:

Descriptive items include: age (years), sex (male /female), weight (kg), height (cm), education, comorbidities, medication and ethnic background.

Questionnaires:

One or two days before each visit, the patient receives an e-mail with a link and is asked to fill out the questionnaires (Table 3). The questionnaires (provided in Dutch) are sent through RoQua (UMCG, Groningen, The Netherlands), a questionnaire program built into the electronic patient's file. The whole set of questionnaires is sent for visit 1a and 3, and it takes about 20 minutes to complete. For visit 1b-1c and 2, only the CSI is sent, and take about 5 minutes to complete. Patients who cannot fill out the questionnaires online will receive them on paper at the beginning of the visit.

Central Sensitisation Inventory (CSI):[22 25] The CSI contains 25 statements related to current health symptoms. Each item is measured on a 5-point Likert scale from 0 (never) to 4 (always). A cut-off score of 40 is being used.[22 25] The original CSI (Cronbach's alpha = 0.879, test-retest reliability = 0.817)[25] as well as the CSI-Dutch language version (Dlv) (Cronbach's alpha = 0.91, test-retest reliability = 0.88 and 0.91)[22] shows a good internal consistency.

Pain Catastrophizing Scale:[26] It assesses thoughts and feelings about pain. For each of 13 statements the participant is asked to answer on a scale from 0 (totally not) to 4 (always). The original pain catastrophizing scale (Cronbach's alpha = 0.93,[27] test-retest reliability =

For peer review only - http://bmjopen.bmj.com/site/about/guidelines.xhtml

0.75)[26] as well as the Pain catastrophizing scale-Dlv (Cronbach's alpha between 0.85 and 0.91)[28-30] show good internal consistency.

Pain Vigilance and Awareness Questionnaire.[31 32] This questionnaire evaluates the awareness of pain. The participant can assign a number from 0 (never) to 5 (continuously) for 16 statements. The original pain vigilance and awareness questionnaire shows good internal consistency (Cronbach's alpha=0.86)[31] and adequate test-retest reliability (r=0.80).[31] The Pain vigilance and awareness questionnaire-Dlv also shows good internal consistency (Cronbach's alpha = 0.87).[32]

RAND 36-item Health Survey (RAND-36):[33 34] The RAND-36 is a participant-reported survey of health-related quality of life. It consists of 8 sections: vitality, physical functioning, bodily pain, general health perceptions, physical role functioning, emotional role functioning, social role functioning and mental health. The RAND-36-Dlv has shown to be valid and reliable.[33]

Pain Disability Index:[35 36] This questionnaire is used to measure the degree to which aspects of the patient's life is disrupted by chronic pain. It lists seven life activity categories (family/home responsibilities, recreation, social activity, occupation, sexual behaviour, self-care and life-support activities). The original pain disability index shows good internal consistency (Cronbach's alpha=0.871),[37] and the test-retest reliability is good (ICC=0.91).[38] The pain disability index-Dlv showed good internal consistency with patients with chronic low back pain (Cronbach's alpha= 0.85)[36] and sufficient test-retest reliability (ICC=0.78).[36]

Work Ability Index:[39] It is used to assess workability during health examinations. The index is determined based on the answers to a series of questions that consider the demands of work, the worker's health status and resources. The original work ability index has been validated (Cronbach's alpha = 0.72).[40 41] The work ability index-Dlv showed sufficient internal consistency (Cronbach's alpha = 0.72)[41] and an acceptable test-retest reliability.[42]

STarT Back Screening Tool (SBST):[43 44] The SBST is a 9-item patient self-reporting questionnaire validated for triage of patients with non-specific low back pain in primary care. It has shown to be helpful in tertiary care.[45] The SBST identifies modifiable prognostic factors from the health domains of pain, activity limitation and psychosocial factors, which are risk factors for persistent non-specific low back pain. The SBST classifies patients into three groups: low, medium or high risk of poor prognosis based on the symptom complexity. When assessed by general practitioners, this classification can assist in making decisions towards appropriate evidence-based treatment pathways. The SBST-Dlv showed good validity and an excellent to fair reproducibility.[44]

For peer review only

For peer review only - http://bmjopen.bmj.com/site/about/guidelines.xhtml

Numeric Rating Scale (NRS) for Pain: A pain rating scale where patients are asked to rate their pain at two locations (leg and back), giving a number between 0 and 10 (0 = no pain/10 = maximum pain imaginable). In the QST measurements, an NRS is also used with a scale from 0 to 100 10 (0 = no pain/100 = maximum pain imaginable).

Drawing in standard leg images (Pain Drawing): The patient is asked to draw, on an image with standard legs (see appendix C), the area where they experience the most pain. When more locations are drawn, an arrow will indicate the most painful spot.

Quantitative Sensory Testing (QST):

QST is a psychophysical method that measures responses to calibrated graded innocuous or noxious stimuli in addition to bedside clinical examination of the somatosensory system.[19 46] The measurements are based on the QST battery developed by the German Research Network on Neuropathic Pain.[10 15] This protocol is extended with cuff algometry (NociTech, Aalborg, Denmark). QST is used for the analyses of HACS. The participants will be comfortable sitting and lying on a bed during all assessments when evaluating the stimuli. The sequences' order is counterbalanced and documented in the case report form (Table 4). The measurements take about 45-60 minutes.

Mechanical Detection Threshold: Mechanical detection threshold is assessed via von Frey-filaments (OptiHair2, MRC Systems GmbH, Heidelberg, Germany). When the 16mN von Frey filament's touch is felt, the von Frey filament's intensity is reduced until no touch is felt. The intensity then will be increased until the touch is felt again. This test will be repeated at least three times to detect the mechanical detection threshold. The first von Frey filament that cannot be felt is documented.[47-49]

Dynamic Mechanical Allodynia: Dynamic mechanical allodynia is tested using dynamic innocuous stimulus (soft brush; Sense Lab Brush no. 5, Somedic, Sösdala, Sweden). The innocuous stimulus is administered once and after that three times in a 1-2 cm wiping motion on the skin. The participant is asked to say if something was felt and to rate the NRS-pain from 0 to 100.[50-52]

Mechanical Pain Threshold: This test is performed with weighted pinprick stimuli (MRC Systems GmbH, Heidelberg, Germany) using the method of limits. Five threshold determinations are made, each with a series of ascending and descending stimulus intensities. The first pinprick that is considered painful is documented.[51 53-55]

Wind-up Ratio: In this test, the NRS-pain score (0-100) experienced for a single pinprick stimuli (256N) is compared to an applied series of repetitive (ten times with intervals of 1

For peer review only - http://bmjopen.bmj.com/site/about/guidelines.xhtml

second) pinprick stimuli of the same intensity (256N). The wind-up ratio is calculated by dividing the pain intensity rating for the series of stimuli by the pain intensity rating for the single stimulus.

Pressure Pain Threshold: Using a handheld pressure algometer (Wagner FDX 10, Greenwich, USA), the threshold for pressure-induced pain is assessed by slowly increasing stimulus intensities (5 N/s). The threshold is determined when the pressure becomes painful/more than just the feeling of pressure when the participants say 'now'. The NRS-pain (0-100) and the applied force (N) at that moment are documented.

Cuff algometry (Cuff): During cuff algometry, the participants will be instructed to continuously evaluate the pain using an electronic visual analogue scale (0 = no pain, 10 = worst imaginable pain).

Cuff pain tolerance threshold: The cuff pressure will be increased by one kPa/s on the dominant leg. The participant will be instructed to rate the pain intensity continuously on the electronic visual analogue scale until the tolerance level is reached, and the participant pushes the stop button. When the participant pushes this button, the pressure is released immediately. The pain tolerance threshold (PTT) is defined as when the participant presses the stop button. A similar assessment will be performed on the non-dominant leg after the cuff temporal summation.

Cuff temporal summation: A total of 10 repeated mechanical cuff pressure stimuli at the intensity of PTT will be delivered at 0.5 Hz (1-second stimulus duration and 1-second interval between stimuli) to the lower leg. A constant pressure of 1 kPa will be applied between the individual pressure stimuli sets to avoid movement of the cuff. The participants will continuously rate the pain intensity on the electronic visual analogue scale during the 10 repeated stimuli.

Conditioned Pain Modulation: Pressure pain threshold will be repeated on the deltoid muscle and the rectus femoris, both on the painful dermatome's contralateral side. The participant will be asked to put the dominant hand up to the wrist with spread fingers in a bucket filled with ice and cold water (conditioning stimulus). The amount of time (at a maximum of three minutes for safety reasons) that it takes until the participant removes the immersed hand out of the bucket will be noted. Immediately after, pressure pain threshold in the deltoid muscle and the rectus femoris both on the contralateral side of the painful dermatome will be repeated.[17 18 20]

For peer review only - http://bmjopen.bmj.com/site/about/guidelines.xhtml

Measurement sites: Measures will be taken at six different sites in total (Table 5). The QST measures will be performed on sites A-D. The conditioned pain modulation will be assessed at sites D and E. The cuff measures will be performed at site F.

Sample size:

The sample size was calculated via G*power for Windows (Version 3.1.9.7, Heinrich Heine Universität, Dusseldorf, Germany) and was calculated to be 41 individuals per group. As a safety margin for possible correction for dropout or missing data we added 20% to result in 50 patients and 50 healthy volunteers.

The required sample size to answer the first objective was based on the difference between two dependent means (matched pairs). Mehta et al [21] described an increase in PPT from 310 ± 90 towards 375 ± 90. The calculated effect size (d) was 0.72 and with a power of 90% and a type I error of 5% the calculated number of patients to be included was 22.

For objective two the sample size calculation was based on the Wilcoxon-Mann-Whitney test for two groups (patients and healthy volunteers). The number of participants to compare the patients with the healthy volunteers was calculated based on the paper by Blumenstiel et al.[56]. The PPT threshold in 23 patients with chronic back pain (mean 239.3 kPa; 95%CI 200-287) compared to 20 healthy controls (mean 352 kpa; 95%CI 286-432). The calculated effect size (d) was 0.75, and with a power of 90% and a type I error of 5% the calculated number of patients and volunteers was in total 82 (41 individuals per group).

Data collection, management, and analysis

Data collection and management:

The primary data (raw) will be collected on paper and entered into an electronic data capture: OpenClinica (OpenClinica, LLC, Waltham, USA, version 3.14). The questionnaires will be assessed via RoQua (RoQua, UMCG, Groningen, The Netherlands) as implemented in the electronic patient files (EPIC, Epic Systems Corporation, Verona, USA, version August 2020). For the healthy volunteers' dummy numbers (for the hospital information system) will be created to enable them to access and fill in the questionnaires. The data in Open Clinica and RoQua will be combined into SPSS (version 27, IBM, Armonk, USA) database.

For peer review only - http://bmjopen.bmj.com/site/about/guidelines.xhtml

The raw data on paper, in OpenClinica and the data of RoQua, will be anonymised and coded. The data will be stored at the UMCG research drive.

Data will be handled confidentially. A participant identification code list will be used to link the data to the participant. The principal investigator will safeguard the key to the code.

Data will be accessible for the team of investigators, the Medical Ethical Review Board and the health care Inspection. Data will be saved for 15 years. Data will be used for publication, but no participant will be traceable. The handling of personal data will comply with the Dutch Personal Data Protection Act.

Statistical methods:

Data records from the different tests and questionnaires will be collected and merged into one database. SPSS software version 27.0 or higher (IBM, Armonk, USA) will be used to perform the analyses. Descriptive statistics data will be presented as means ± standard deviation (SD) when normally distributed and as median and interquartile range (IQR) (25-75) when not normally distributed. A significance level of 0.05 is used in all analyses.

For the primary objective, to determine treatment effect of the tSNRB and pRF the difference between visit 1a and visit 3 will be analysed for nine primary measures (CSI and eight QST measures). This analysis will be performed using a student’s paired t-test in normally distributed data or a Wilcoxon test in non-normally distributed data.

For secondary objective 1, to determine if the presence of HACS in patients with CLBPr can be assessed using the CSI, the data from visit 1a will be used.

For secondary objective 2, the difference between the latest visit 1 and visit 2 will be used to determine treatment effect of the dSNRB. The nine primary measures (CSI and eight QST measures) will be used. The difference will be assessed using a student’s paired t-test or a Wilcoxon test where appropriate.

For secondary objective 3, the differences between patients’ visit 1a, vs visit 3 and healthy volunteers are calculated for the questionnaires and QST measurements. The calculated differences (patients’ visit 1a vs healthy volunteers and patients’ visit 3 vs healthy volunteers) will be assessed using an a student t-test for normally distributed data and a Mann-Whitney-U test for non-normally distributed data

Monitoring

Data monitoring:

For peer review only

For peer review only - http://bmjopen.bmj.com/site/about/guidelines.xhtml

The monitoring will be carried out by research support staff based on GCP monitoring principles. The departmental research office will monitor the trial regularly. This typically includes the following checks:

1) The data collected are consistent with adherence to the trial protocol; 2) Case report files are completed by authorised persons; 3) No key data are missing; 4) The data appears to be valid (i.e. range and outlier checks); 5) Review of recruitment rates, withdrawals and losses to follow-up.

Harms:

Experienced specialised pain staff members perform all interventions in the care as usual setting. Study-related additional harms may consist of QST-related pain during and a short time after the measurements. No other harm is expected.

Protocol amendments:

The following amendments were made before the inclusion of the fifth patient. Regarding the Inclusion criteria, the age limit of 65 was excluded. The visual analogue scale was changed to NRS because NRS is used in care as usual. Before the first healthy volunteer's inclusion, the number of visits was reduced from three to one. The latest protocol, version 5, is from 06-07-2020. Due to the COVID-19 pandemic, the study was put on hold, and an amendment (version 5) was submitted and approved on August 11, 2020, to prolong the initial inclusion date for the participants to March 1, 2022.

Declaration of interest:

Not applicable

IPD sharing statement

The de-identified individual clinical trial participant-level data (IPD) will be shared. All individual participant data that underlie the results reported in this article will be shared after de-identification (text, tables, figures, and appendices). The study protocol will also be available. The data will be available beginning nine months following article publication and for a maximum period of 15 years. To make the data findable and accessible for others, we will include a description of the UMCG data catalogue data: https://www.groningendatacatalogus.nl/. Researchers who provide a methodologically sound proposal can access the data with a signed data access agreement.

For peer review only

For peer review only - http://bmjopen.bmj.com/site/about/guidelines.xhtml

TRIAL STATUS

This trial is ongoing. The planned end date for the recruitment of patients and healthy volunteers is March 1, 2022

Authors Contributions

HT, GG, KP, LAN and AW designed the trial protocol. GG and AW secured funding for the study. IS, HT and AW drafted the manuscript. GG, LAN and KP contributed to the manuscript. All authors read and approved the final manuscript. IS, HT and AW are involved in data collection during the study. All authors are involved in the data analysis and manuscript preparations after the study is completed.

Competing interests statement

The authors declare that they have no competing interests.

Funding

The University of Groningen provided funding, University Medical Center Groningen, the Graduate Sçhool of Medicine and Department of Anesthesiology, Pain Center, Groningen, the Netherlands and the Ministry of Education of Brazil - Science without Borders programme (PhD scholarship to Ms Tharcila Chaves – project number 9140/13-1), Rio de Janeiro, Brazil. The funding bodies had no role in the study design and collection, analysis and interpretation of data, and writing of the manuscript.

For peer review only

For peer review only - http://bmjopen.bmj.com/site/about/guidelines.xhtml

REFERENCES

1. Spijker-Huiges A, Groenhof F, Winters JC, et al. Radiating low back pain in general practice: incidence, prevalence, diagnosis, and long-term clinical course of illness. *Scand J Prim Health Care* 2015;33(1):27-32. doi: 10.3109/02813432.2015.1006462 [published Online First: 2015/02/20]
2. Bogduk N, McGuirk B. Medical management of acute and chronic low back pain: an evidence-based approach. Amsterdam2002.
3. Wolff AP, Groen GJ, Wilder-Smith OH. Diagnosis of chronic radiating lower back pain without overt focal neurologic deficits: what is the value of segmental nerve blocks? *Therapy* 2005;2(4):577-85.
4. Wolff AP, Groen GJ, Crul BJ. Diagnostic lumbosacral segmental nerve blocks with local anesthetics: a prospective double-blind study on the variability and interpretation of segmental effects. *Reg Anesth Pain Med* 2001;26(2):147-55. doi: 10.1053/rapm.2001.21436 [published Online First: 2001/03/17]
5. Stolker RJ, Vervest ACM, Groen GJ. The management of chronic spinal pain by blockades: a review. *Pain* 1994;58(1):1-20.
6. Tajima T, Furamochi K, Kuramochi E. Selective Lumbosacral Radiculography and Block. *Spine* 1980;5(1):68-77.
7. Krempen JF, Smith BS. Nerve-root injection: a method for evaluating the etiology of sciatica. *JBJS* 1974;56(7):1435-44.
8. Herron LD. Selective nerve root block in patient selection for lumbar surgery: surgical results. *Journal of spinal disorders* 1989;2(2):75-79.
9. Dooley J, McBroom R, Taguchi T, et al. Nerve root infiltration in the diagnosis of radicular pain. *Spine* 1988;13(1):79-83.
10. Pelletier R, Higgins J, Bourbonnais D. Is neuroplasticity in the central nervous system the missing link to our understanding of chronic musculoskeletal disorders? *BMC Musculoskelet Disord* 2015;16:25. doi: 10.1186/s12891-015-0480-y [published Online First: 2015/04/19]
11. Arendt-Nielsen L, Morlion B, Perrot S, et al. Assessment and manifestation of central sensitisation across different chronic pain conditions. *Eur J Pain* 2018;22(2):216-41. doi: 10.1002/ejp.1140 [published Online First: 2017/11/07]
12. Müller M, Curatolo M, Limacher A, et al. Predicting transition from acute to chronic low back pain with quantitative sensory tests—a prospective cohort study in the primary care setting. *European journal of pain* 2019;23(5):894-907.
13. van Helmond N, Aarts HM, Timmerman H, et al. Is preoperative quantitative sensory testing related to persistent postsurgical pain? A systematic literature review. *Anesthesia & Analgesia* 2020;131(4):1146-55.
14. Petersen KK, Vaegter HB, Stubhaug A, et al. The predictive value of quantitative sensory testing: a systematic review on chronic postoperative pain and the analgesic effect of pharmacological therapies in patients with chronic pain. *Pain* 2021;162(1):31-44.
15. Bridges D, Thompson SW, Rice AS. Mechanisms of neuropathic pain. *Br J Anaesth* 2001;87(1):12-26. doi: 10.1093/bja/87.1.12 [published Online First: 2001/07/20]
16. Finco G, Evangelista M, Sardo S. Basic guide to chronic pain assessment: from neurophysiology to bedside. *Minerva Anestesiologica* 2020;86(12):1321-30.
17. Woolf CJ. Central sensitization: implications for the diagnosis and treatment of pain. *Pain* 2011;152(3 Suppl):S2-15. doi: 10.1016/j.pain.2010.09.030 [published Online First: 2010/10/22]
18. von Hehn CA, Baron R, Woolf CJ. Deconstructing the neuropathic pain phenotype to reveal neural mechanisms. *Neuron* 2012;73(4):638-52. doi: 10.1016/j.neuron.2012.02.008 [published Online First: 2012/03/01]

For peer review only

For peer review only - http://bmjopen.bmj.com/site/about/guidelines.xhtml

19. Konopka KH, Harbers M, Houghton A, et al. Bilateral sensory abnormalities in patients with unilateral neuropathic pain; a quantitative sensory testing (QST) study. *PLoS One* 2012;7(5):e37524. doi: 10.1371/journal.pone.0037524 [published Online First: 2012/05/26]
20. Rolke R, Magerl W, Campbell KA, et al. Quantitative sensory testing: a comprehensive protocol for clinical trials. *Eur J Pain* 2006;10(1):77-88. doi: 10.1016/j.ejpain.2005.02.003 [published Online First: 2005/11/18]
21. Mehta V, Snidvongs S, Ghai B, et al. Characterization of peripheral and central sensitization after dorsal root ganglion intervention in patients with unilateral lumbosacral radicular pain: a prospective pilot study. *Br J Anaesth* 2017;118(6):924-31. doi: 10.1093/bja/aex089 [published Online First: 2017/06/03]
22. Kregel J, Vuijk PJ, Descheemaeker F, et al. The Dutch Central Sensitization Inventory (CSI): Factor Analysis, Discriminative Power, and Test-Retest Reliability. *Clin J Pain* 2016;32(7):624-30. doi: 10.1097/AJP.0000000000000306 [published Online First: 2015/09/30]
23. Platform WHOICTR. WHO Trial Registration Data Set (Version 1.3) [Available from: http://awareness.who.int/ictrp/network/trds_v1.3/en/ accessed 5 January 2021.
24. Chan A-W, Tetzlaff JM, Altman DG, et al. SPIRIT 2013 statement: defining standard protocol items for clinical trials. *Annals of internal medicine* 2013;158(3):200-07.
25. Mayer TG, Neblett R, Cohen H, et al. The development and psychometric validation of the central sensitization inventory. *Pain Practice* 2012;12(4):276-85.
26. Sullivan MJ, Bishop SR, Pivik J. The pain catastrophizing scale: development and validation. *Psychological assessment* 1995;7(4):524.
27. Osman A, Barrios FX, Kopper BA, et al. Factor structure, reliability, and validity of the Pain Catastrophizing Scale. *Journal of behavioral medicine* 1997;20(6):589-605.
28. Crombez G, Eccleston C, Baeyens F, et al. When somatic information threatens, catastrophic thinking enhances attentional interference. *Pain* 1998;75(2-3):187-98.
29. Crombez G, Vlaeyen JW, Heuts PH, et al. Pain-related fear is more disabling than pain itself: evidence on the role of pain-related fear in chronic back pain disability. *Pain* 1999;80(1-2):329-39.
30. Van Damme S, Crombez G, Bijttebier P, et al. A confirmatory factor analysis of the Pain Catastrophizing Scale: invariant factor structure across clinical and non-clinical populations. *Pain* 2002;96(3):319-24.
31. McCracken LM. “Attention” to pain in persons with chronic pain: a behavioral approach. *Behavior therapy* 1997;28(2):271-84.
32. Roelofs J, Peters ML, McCracken L, et al. The pain vigilance and awareness questionnaire (PVAQ): further psychometric evaluation in fibromyalgia and other chronic pain syndromes. *Pain* 2003;101(3):299-306.
33. Van der Zee K, Sanderman R. RAND-36. *Groningen: Northern Centre for Health Care Research, University of Groningen, the Netherlands* 1993;28(6)
34. Hays RD, Sherbourne CD, Mazel RM. The rand 36-item health survey 1.0. *Health economics* 1993;2(3):217-27.
35. Pollard CA. Preliminary validity study of the pain disability index. *Perceptual and motor skills* 1984
36. Soer R, Köke AJ, Vroomen PC, et al. Extensive validation of the pain disability index in 3 groups of patients with musculoskeletal pain. *Spine* 2013;38(9):E562-E68.
37. Tait RC, Pollard CA, Margolis RB, et al. The Pain Disability Index: psychometric and validity data. *Arch Phys Med Rehabil* 1987;68(7):438-41.
38. Grönblad M, Hupli M, Wennerstrand P, et al. Intercorrelation and test-retest reliability of the Pain Disability Index (PDI) and the Oswestry Disability Questionnaire (ODQ) and their correlation with pain intensity in low back pain patients. *The Clinical journal of pain* 1993;9(3):189-95.
39. Ilmarinen J. The work ability index (WAI). *Occupational medicine* 2007;57(2):160-60.
40. Tuomi K, Ilmarinen J, Jahkola A, et al. Work ability index: Finnish Institute of Occupational Health Helsinki 1998.

For peer review only

For peer review only - http://bmjopen.bmj.com/site/about/guidelines.xhtml

41. Psychometric properties of Work Ability Index in the light of comparative survey study. International congress series; 2005. Elsevier.

42. De Zwart B, Frings-Dresen M, Van Duivenbooden J. Test–retest reliability of the Work Ability Index questionnaire. *Occupational medicine* 2002;52(4):177-81.

43. Hill JC, Whitehurst DG, Lewis M, et al. Comparison of stratified primary care management for low back pain with current best practice (STarT Back): a randomised controlled trial. *The Lancet* 2011;378(9802):1560-71.

44. Bier JD, Ostelo RW, van Hooff ML, et al. Validity and reproducibility of the STarT Back Tool (Dutch Version) in Patients With Low Back Pain in Primary Care Settings. *Physical Therapy* 2017;97(5):561-70.

45. Storm L, Rousing R, Andersen M, et al. Usefulness of the STarT Back Screening Tool to predict pain problems after lumbar spine surgery. *Dan Med J* 2018;65(12):1-4.

46. Backonja MM, Attal N, Baron R, et al. Value of quantitative sensory testing in neurological and pain disorders: NeuPSIG consensus. *Pain* 2013;154(9):1807-19. doi: 10.1016/j.pain.2013.05.047 [published Online First: 2013/06/08]

47. Weinstein S. Intensive and extensive aspects of tactile sensitivity as a function of body part, sex and laterality. *The skin senses* 1968

48. Fruhstorfer H, Gross W, Selbmann O. von Frey hairs: new materials for a new design. *European Journal of Pain* 2001;5(3):341-42.

49. Baumgärtner U, Magerl W, Klein T, et al. Neurogenic hyperalgesia versus painful hypoalgesia: two distinct mechanisms of neuropathic pain. *Pain* 2002;96(1-2):141-51.

50. LaMotte RH, Shain CN, Simone DA, et al. Neurogenic hyperalgesia: psychophysical studies of underlying mechanisms. *Journal of neurophysiology* 1991;66(1):190-211.

51. Ziegler E, Magerl W, Meyer R, et al. Secondary hyperalgesia to punctate mechanical stimuli: central sensitization to A-fibre nociceptor input. *Brain* 1999;122(12):2245-57.

52. Samuelsson M, Leffler A-S, Hansson P. Dynamic mechanical allodynia: on the relationship between temporo-spatial stimulus parameters and evoked pain in patients with peripheral neuropathy. *Pain* 2005;115(3):264-72.

53. Chan A, MacFarlane I, Bowsher D, et al. Weighted needle pinprick sensory thresholds: a simple test of sensory function in diabetic peripheral neuropathy. *Journal of Neurology, Neurosurgery & Psychiatry* 1992;55(1):56-59.

54. Greenspan JD, McGillis SL. Thresholds for the perception of pressure, sharpness, and mechanically evoked cutaneous pain: effects of laterality and repeated testing. *Somatosensory & motor research* 1994;11(4):311-17.

55. Hampf G, Bowsher D, Wells C, et al. Sensory and autonomic measurements in idiopathic trigeminal neuralgia before and after radiofrequency thermocoagulation: differentiation from some other causes of facial pain. *Pain* 1990;40(3):241-48.

56. Blumenstiel K, Gerhardt A, Rolke R, et al. Quantitative sensory testing profiles in chronic back pain are distinct from those in fibromyalgia. *The Clinical journal of pain* 2011;27(8):682-90.

For peer review only

TABLES

Table 1: Criteria for the inclusion and exclusion of patients

Inclusion criteria	Exclusion criteria
• Male and female patients<br>• Age 18 years or older<br>• Presence of chronic low back pain radiating in the leg<br>• Leg pain more or equal to back pain<br>• A physician must consider therapeutic sensory nerve root blocking or pulsed radiofrequency as an appropriate treatment intervention<br>• Agreement and signature of the informed consent	• Exclusion criteria for selective nerve blocks, according to local protocol<br>• Not or not sufficient understanding of the Dutch language<br>• Incapacity to follow instructions<br>• Mental incompetence to provide informed consent<br>• Chronic low back pain with radiation to both legs<br>• Pain in one (or more) sites where quantitative sensory testing will be applied except for the most painful point in the painful dermatome

For peer review only

For peer review only - http://bmjopen.bmj.com/site/about/guidelines.xhtml

Table 2: Criteria for the inclusion and exclusion of healthy **volunteers**

Inclusion criteria	Exclusion criteria
• Male and female healthy volunteers<br>• Age 18 years or older<br>• No history of low back pain<br>• Agreement and signature of the consent	• Not matching with one of the included patients based on sex and age (plus or minus three years of age)<br>• Not or not sufficient understanding of the Dutch language<br>• Incapacity to follow instructions<br>• Mental incompetence to provide informed consent

For peer review only

For peer review only - http://bmjopen.bmj.com/site/about/guidelines.xhtml

Table 3: Overview of the variables collected and interventions per visit

	Patients					Healthy volunteers
Demographics	Age, gender, weight, height, co-morbidities, pain medication, ethnic background and education.					
		V1a	V1b, V1c	V2	V3	HV
Time between visits		-	One week	One week	Four weeks	-
Questionnaires	CSI	✓	✓	✓	✓	✓
	SBST	✓	-	-	✓	✓
	RAND-36	✓	-	-	✓	✓
	PDI	✓	-	-	✓	✓
	WAI	✓	-	-	✓	✓
	PVAQ	✓	-	-	✓	✓
	PCS	✓	-	-	✓	✓
	Pain Drawing	✓	-	-	✓	-
	NRS pain	✓	✓	✓	✓	✓
QST	MDT	✓	✓	✓	✓	✓
	DMA	✓	✓	✓	✓	✓
	MPT	✓	✓	✓	✓	✓
	WUR	✓	✓	✓	✓	✓
	PPT	✓	✓	✓	✓	✓
	Cuff PPT	✓	✓	✓	✓	✓
	Cuff TS	✓	✓	✓	✓	✓
	CPM	✓	✓	✓	✓	✓
Intervention	dSNRB	✓	✓	-	-	-
	tSNRB or pRF	-	-	✓	-	-

Legend: V: visit, HV: healthy volunteers, QST: quantitative sensory testing; MDT: mechanical detection threshold; DMA: dynamic mechanical allodynia; MPT: mechanical pain threshold; WUR: Wind-up ratio; PPT: pressure pain threshold; Cuff PPT: pressure pain threshold by cuff algometer; Cuff TS: temporal summation by cuff algometer; CPM: conditioned pain

For peer review only - http://bmjopen.bmj.com/site/about/guidelines.xhtml

modulation; CSI: Central Sensitisation Inventory; SBST: STarT Back Screening Tool; RAND-36: RAND 36-Item Health Survey; PDI: Pain Disability Index; WAI: Work Ability Index; PVAQ: Pain Vigilance and Awareness Questionnaire; PCS: Pain Catastrophising Scale; Pain Drawing: Drawing in standard leg images. NRS-pain: Numeric Rating Scale for Pain; dSNRB: diagnostic selective nerve root block, tSNRB: therapeutic selective nerve root block, pRF: pulsed radiofrequency

For peer review only

For peer review only - http://bmjopen.bmj.com/site/about/guidelines.xhtml

Table 4: Sequence of QST measurement sites

Visit	Sequence
Visit 1a	Site D-A-C-B
Visit 1b	Site D-C-A-B
Visit 1c	Site D-B-A-C
Visit 2	Site D-C-B-A
Visit 3	Site D-B-C-A

For peer review only

For peer review only - http://bmjopen.bmj.com/site/about/guidelines.xhtml

Table 5: QST measurement sites:

Site A	The most painful point in the painful dermatome
Site B	Contralateral point of location A
Site C	Control site 1 (contralateral of location A, distant from the painful area), between the scapulae. On the medial part of trapezius muscle at the height of the spina scapulae, 4cm lateral to the spinous process of the 3rd thoracic vertebra (Th3)
Site D	Control site 2 (distant from the painful area): contralateral to location A, at the deltoid muscle: On the medial part of the deltoid muscle, 3cm below the acromion
Site E	On the rectus femoris muscle 15 cm proximal to the base of the patella
Site F	On the lower leg. At the level of the largest circumference of gastrocnemius muscle.

For peer review only

For peer review only - http://bmjopen.bmj.com/site/about/guidelines.xhtml

Figure 1: Flowchart of the study procedure

Legend: QST: Quantitative sensory testing; dSNRB: diagnostic selective nerve root block; tSNRB: therapeutic selective nerve root block; pRF: Pulsed radiofrequency.

For peer review only

For peer review only - http://bmjopen.bmj.com/site/about/guidelines.xhtml

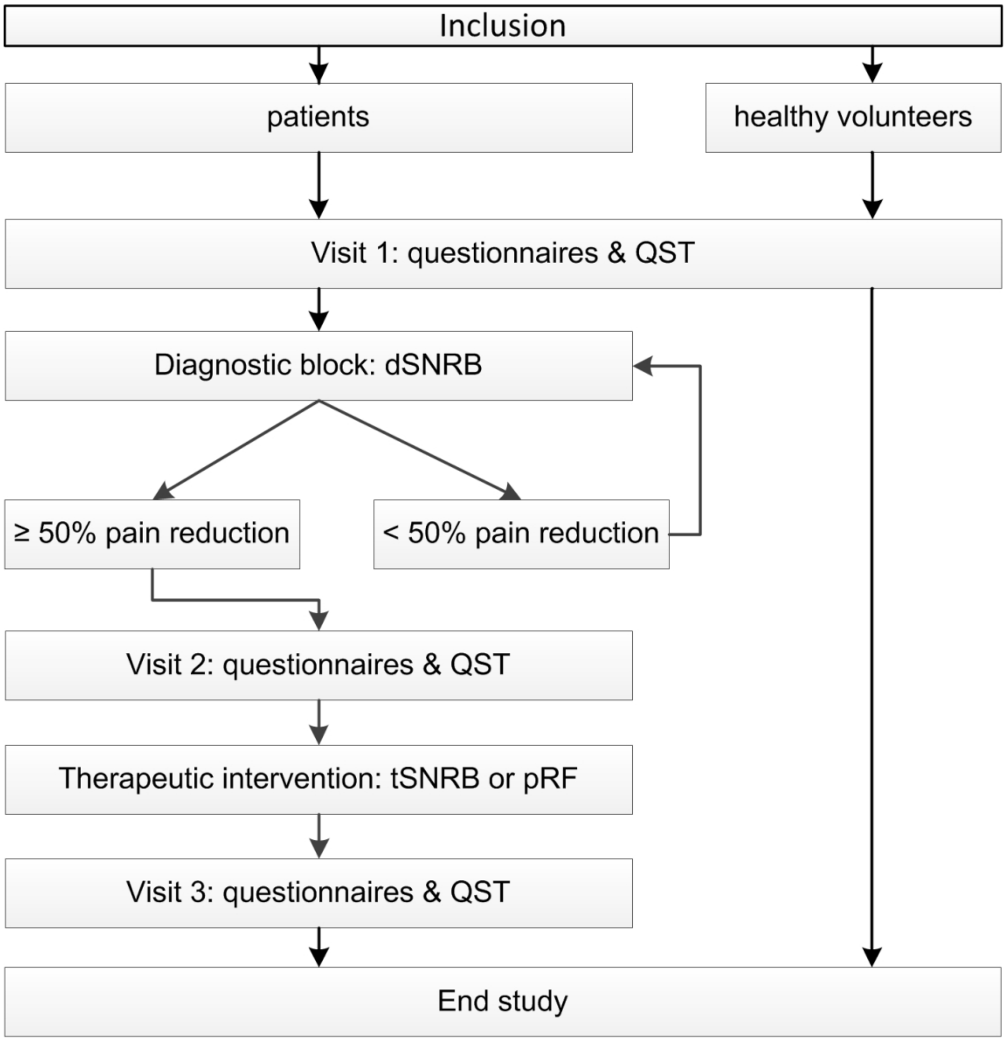

Figure 1: Flowchart of the study procedure / QST: Quantitative sensory testing; dSNRB: diagnostic selective nerve root block; tSNRB: therapeutic selective nerve root block; pRF: Pulsed radiofrequency.

119x124mm (300 x 300 DPI)

APPENDICES

Appendix A: Informed consent materials:

A model consent form and other related documentation is, in Dutch, given to participants and authorised surrogates

Bijlage B:

Toestemmingsformulier proefpersoon

Onderzoek naar de gevoeligheid van het centrale zenuwstelsel bij proefpersonen met chronische lage rugpijn uitstralend naar het been.

(Centrale sensitisatie in proefpersonen met chronische lage rugpijn uitstralend naar het been)

- Ik heb de informatiebrief gelezen. Ook kon ik vragen stellen. Mijn vragen zijn voldoende beantwoord. Ik had genoeg tijd om te beslissen of ik meedoe.
- Ik weet dat meedoen vrijwillig is. Ook weet ik dat ik op ieder moment kan beslissen om toch niet mee te doen of te stoppen met het onderzoek. Daarvoor hoef ik geen reden te geven.
- Ik weet dat sommige mensen mijn persoonsgegevens en/of gecodeerde onderzoeksgegevens kunnen inzien. Die mensen staan vermeld in deze informatiebrief.
- Ik geef toestemming om mijn gegevens nog 15 jaar na dit onderzoek te bewaren.
- Ik wil meedoen aan dit onderzoek.

Naam proefpersoon:

Handtekening: Datum : __ / __ / __

Ik verklaar dat ik deze proefpersoon volledig heb geïnformeerd over het genoemde onderzoek.

Als er tijdens het onderzoek informatie bekend wordt die de toestemming van de proefpersoon zou kunnen beïnvloeden, dan breng ik hem/haar daarvan tijdig op de hoogte.

Naam onderzoeker (of diens vertegenwoordiger):

Handtekening: Datum: __ / __ / __

<indien van toepassing>

Aanvullende informatie is gegeven door:

Naam:

Functie:

Handtekening: Datum: __ / __ / __

----

* Doorhalen wat niet van toepassing is.

De proefpersoon krijgt een volledige informatiebrief mee, samen met een kopie van het getekende toestemmingsformulier.

For peer review only - http://bmjopen.bmj.com/site/about/guidelines.xhtml

Appendix B: Intervention procedures

dSNRB

Diagnostic selective nerve root blocks (dSNRB) are performed in a fluoroscopy suite with monitoring equipment. At a minimum, pulse and blood pressure should be monitored every 1 to 5 minutes, depending on the procedure. Assisting personnel include nurses trained in advanced cardiac life support and a radiology technician. All procedures are performed with fluoroscopic guidance. Patient monitoring does not end after injecting medication. Post-procedure assessment is necessary because side effects or complications such as allergic reaction, vasovagal response, cardiac or respiratory depression, or high spinal block can occur. To ascertain whether the diagnostic selective nerve root block response was positive or negative, patients should perform activities or assume positions that invariably incite their pain. If a change in preprocedure and postprocedure verbal numeric rating scale rating of a minimum of 50% pain relief occurs, then the dSNRB result is considered positive.

For a lumbar dSNRB, the patient is placed in a prone position on the fluoroscopy table. The back is prepared and draped in a sterile manner. The level is located by establishing the lowest lumbar segment. The C-arm is rotated in the oblique position to allow access to the intervertebral foramen. Care must be maintained not to start too laterally to avoid renal or peritoneal puncture. A skin wheal is raised with 1% xylocaine. A 22-gauge, 6 or 10 cm with 5mm nonisolated tip needle is directed toward the foramen in the oblique position. Once the needle has penetrated the muscle adjacent to the spine, the needle then is advanced slowly in the AP plane to the 6-o'clock position of the pedicle and just inferior to the pedicle. The needle's position is in the safe triangle formed by the pedicle, the foramen's outer line, and the spinal nerve. Care is taken not to pierce the spinal nerve. Needle position is checked in the AP and lateral planes. One millilitre of non-ionic contrast agent is injected to outline the spinal nerve and dorsal root ganglion. The contrast pattern is evaluated to ensure that there is no vascular pattern, subarachnoid flow, or epidural spread to adjacent levels. One millilitre of 0.75% 0.3ml with Visipaque 320 mg I/ml 0.3ml (total 0.6ml) is infused.

Reference

Gauci, C. A. (2011). Manual of RF Techniques-a Practical Manual of Radiofrequency Procedures in Chronic Pain Management(3th Edition), CoMedical, 2011. Ridderkerk, the Netherlands.

Huston, C. W., & Slipman, C. W. (2002). Diagnostic selective nerve root blocks: indications and usefulness. *Physical medicine and rehabilitation clinics of North America*, 13(3), 545-565.

For peer review only - http://bmjopen.bmj.com/site/about/guidelines.xhtml

Perdok, J.M., Wolff, A.P., Stellema, R., Groen, G.J. (2017) 'Wortelblokkades'. Local protocol University Medical Center Groningen.

tSNRB

Therapeutic selective nerve root block (tSNRB) is similar to the dSNRB. The tSNRB is performed under sterile conditioned with an image intensifier. The target point was "safe triangle," i.e., above the exiting nerve root and below the corresponding pedicle. The skin is anaesthetised with 2-3 ml mepivacaine 2%. A spinal needle (22G) was inserted paramedian through the skin and muscles in a cranio-medial direction until a bony contact was encountered. This method allows advancing the needle in a safe triangle without contact with the nerve root. After verifying a correct needle positioning under biplanar image intensifier control, non-ionic contrast was injected until a radiculogram is obtained. Subsequently, 1ml 0.75% Bupivacaine and 1ml (40mg) Triamcinolon and 320mg I/ml Visipaque is injected. It should be stressed that this was a periradicular and not an intraneural injection. This technique is for the nerve roots L3-L5; for S1, a different technique is required. First, the image intensifier is positioned perpendicular to the foramen S1. A spinal needle is inserted perpendicular to the surface of the sacrum into the foramen. An image intensifier checks the correct needle positioning in two planes. After obtaining a correct periradiculargram, 1ml 0.75% Bupivacaine and 1ml (40mg) Triamcinolon and 320mg I/ml Visipaque is injected. The patients have clinical surveillance on the ward for about 30 minutes to account for unexpected side effects.

Reference

Bogduk N, Aprill C, Derby R, Selective nerve root blocks. In: Wilson DJ, ed. Interventional Radiology of the Muskuloskeletal System. London: Edward Arnold; 1995. p. 122–32.

Gauci, C. A. (2011). Manual of RF Techniques-a Practical Manual of Radiofrequency Procedures in Chronic Pain Management(3th Edition), CoMedical, 2011. Ridderkerk, the Netherlands.

Perdok, J.M., Wolff, A.P., Stellema, R., Groen, G.J. (2017) 'Wortelblokkades'. Local protocol University Medical Center Groningen.

Narozny, M., Zanetti, M., & Boos, N. (2001). Therapeutic efficacy of selective nerve root blocks in the treatment of lumbar radicular leg pain. *Swiss medical weekly*, *131*(0506).

pRF

For peer review only - http://bmjopen.bmj.com/site/about/guidelines.xhtml

Pulsed radiofrequency (pRF) is in a procedure similar to the tSNRB. The pRF is performed under sterile conditioned with an image intensifier. The target point was "safe triangle," i.e., above the exiting nerve root and below the corresponding pedicle. The skin is anaesthetised with 2-3 ml mepivacaine 2%. A spinal needle (22G) was inserted paramedian through the skin and muscles in a cranio-medial direction until a bony contact was encountered. This method allows advancing the needle in a safe triangle without contact with the nerve root. After verifying a correct needle positioning under biplanar image intensifier control, non-ionic contrast was injected until a radiculogram is obtained.

The tissue temperature is held at or below 42˚C on average. Radiofrequency is delivered in short, high-intensity bursts so that the radiofrequency electric field strength is increased without gross heating. The clinical objective is a neural modification by electric and thermal fields. This technique is for the nerve roots L3-L5; for S1, a different technique is required. First, the image intensifier is positioned perpendicular to the foramen S1. A spinal needle is inserted perpendicular to the surface of the sacrum into the foramen. An image intensifier checks the correct needle positioning in two planes. After obtaining a correct periradiculargram, the tissue temperature is held at or below 42˚C on average. Radiofrequency is delivered in short, high-intensity bursts so that the radiofrequency electric field strength is increased without gross heating. The patients have clinical surveillance on the ward for about 30 minutes to account for any unexpected side effects

Reference

Ewertowska, E., Mercadal, B., Muñoz, V., Ivorra, A., Trujillo, M., & Berjano, E. (2018). Effect of applied voltage, duration and repetition frequency of RF pulses for pain relief on temperature spikes and electrical field: a computer modelling study. *International Journal of Hyperthermia*, *34*(1), 112-121.

Gauci, C. A. (2011). Manual of RF Techniques-a Practical Manual of Radiofrequency Procedures in Chronic Pain Management(3th Edition), CoMedical, 2011. Ridderkerk, the Netherlands.

Perdok, J.M., Wolff, A.P., Stellema, R., Groen, G.J. (2017) 'Wortelblokkades'. Local protocol University Medical Center Groningen.

For peer review only - http://bmjopen.bmj.com/site/about/guidelines.xhtml

Appendix C: Images drawing the most painful spot on the leg.

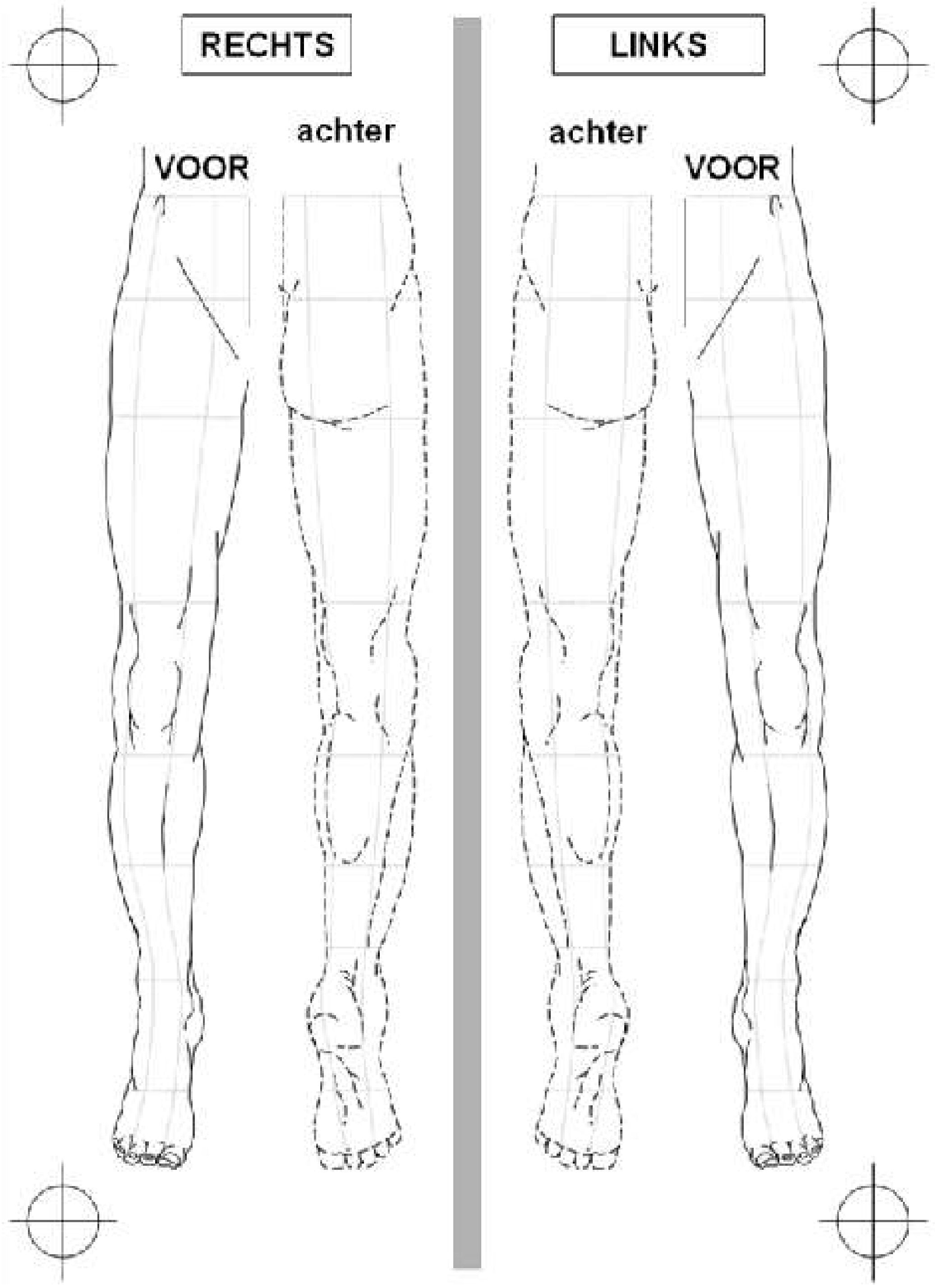

For peer review only - http://bmjopen.bmj.com/site/about/guidelines.xhtml
